# Mapping Aldehyde Dehydrogenase 1A1 Activity using an [^18^F]Substrate‐Based Approach

**DOI:** 10.1002/chem.201805473

**Published:** 2019-01-14

**Authors:** Raul Pereira, Thibault Gendron, Chandan Sanghera, Hannah E. Greenwood, Joseph Newcombe, Patrick N. McCormick, Kerstin Sander, Maya Topf, Erik Årstad, Timothy H. Witney

**Affiliations:** ^1^ Centre for Advanced Biomedical Imaging University College London Paul O'Gorman Building, 72 Huntley Street London WC1E 6DD UK; ^2^ Current address: Department of Imaging Chemistry & Biology King's College London, St. Thomas' Hospital London SE1 7EH UK; ^3^ Department of Chemistry University College London 20 Gordon Street London WC1H 0AJ UK; ^4^ Department of Biological Sciences, Birkbeck University of London Malet Street London WC1E 7HX UK

**Keywords:** [^18^F]fluorination, aldehyde dehydrogenase, cancer, radiochemistry, radiolabeling

## Abstract

Aldehyde dehydrogenases (ALDHs) catalyze the oxidation of aldehydes to carboxylic acids. Elevated ALDH expression in human cancers is linked to metastases and poor overall survival. Despite ALDH being a poor prognostic factor, the non‐invasive assessment of ALDH activity in vivo has not been possible due to a lack of sensitive and translational imaging agents. Presented in this report are the synthesis and biological evaluation of ALDH1A1‐selective chemical probes composed of an aromatic aldehyde derived from *N*,*N*‐diethylamino benzaldehyde (DEAB) linked to a fluorinated pyridine ring either via an amide or amine linkage. Of the focused library of compounds evaluated, *N*‐ethyl‐6‐(fluoro)‐*N‐*(4‐formylbenzyl)nicotinamide **4 b** was found to have excellent affinity and isozyme selectivity for ALDH1A1 in vitro. Following ^18^F‐fluorination, [^18^F]**4 b** was taken up by colorectal tumor cells and trapped through the conversion to its ^18^F‐labeled carboxylate product under the action of ALDH. In vivo positron emission tomography revealed high uptake of [^18^F]**4 b** in the lungs and liver, with radioactivity cleared through the urinary tract. Oxidation of [^18^F]**4 b**, however, was observed in vivo, which may limit the tissue penetration of this first‐in‐class radiotracer.

## Introduction

Aldehyde dehydrogenases (ALDHs) are a family of enzymes that catalyze the NAD(P)^+^‐dependent oxidation of a wide variety of aldehydes to their corresponding carboxylic acids.^1^ There are currently 20 known functional human ALDHs^2^ that mediate the metabolism of aldehydes generated during oxidative stress,^3^ amino acid and biogenic amine metabolism,^4^ retinoic acid biosynthesis,^5^ and ethanol metabolism.3a In addition, ALDHs control the detoxification of exogenous reactive aldehydes and therapeutic drugs such as cyclophosphamide.^6^ Aberrant expression of ALDH is associated with many diseases, including cancer, with increased expression and activity of ALDH shown to be a predictor of metastatic potential and poor overall survival.^7^ In particular, the ALDH1A1 isozyme is a well‐characterized marker of cancer stem cells, which are known for their tumor‐initiating properties and resistance to conventional therapy.^8^ Studies have shown resistance to chemotherapy and poor prognosis is associated with high ALDH1A1 activity in breast,^9^ ovarian,^10^ prostate,^11^ colon^12^ and lung^13^ cancer. As a consequence, ALDH1A1 has been selected as a target for anti‐cancer therapy, with ALDH inhibitors shown to reverse chemoresistance in a range of preclinical tumor models.^14^

Given the causal link between ALDH expression and cancer drug resistance, the non‐invasive identification of ALDH‐expressing tumors is of great clinical importance. The measurement of chemoresistance through ALDH imaging could potentially enable the clinician to select the most suitable therapeutic intervention for the individual patient (e.g. chemotherapy versus immunotherapy) with the possibility to improve outcomes and reduce unnecessary treatment. Currently, the in vitro assessment of ALDH activity has been restricted to fluorescence‐based assays.^15^ Despite these commercially available imaging agents being widely‐adopted for the isolation of ALDH‐positive cells in cell culture, the poor tissue penetration of the fluorescent signal currently limits their in vivo utility. In order to circumvent these inherent limitations, we propose the use of positron emission tomography (PET) as an alternative to fluorescence‐based assays.^16^ Previous attempts to develop ALDH1A1‐specific radiotracers have so far failed due to the poor cellular retention of the carboxylate product, presumed to be a consequence of its high hydrophobicity.^17^ Here, we report the synthesis and biological evaluation of ^18^F‐fluorinated aldehyde‐based probes for the non‐invasive detection of ALDH1A1 activity in tumor cell models.

## Results and Discussion

**Figure 1:**
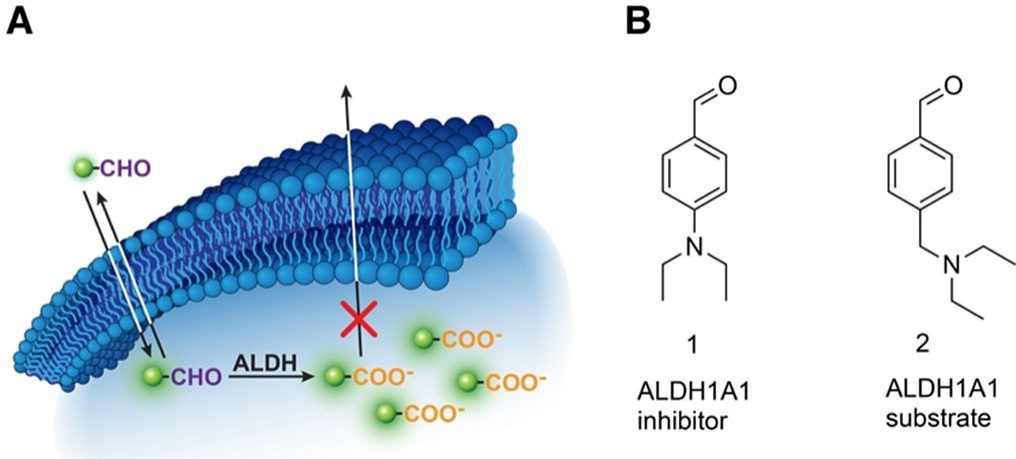
A) Schematic illustrating ALDH‐mediated trapping of ^18^F‐labeled aldehydes by conversion to their corresponding acid. B) Chemical structures of DEAB (**1**), an ALDH1A1 inhibitor, and **2**, an ALDH1A1 substrate.

ALDH1A1 chemical probes were designed to have a) an aldehyde that can serve as a substrate for ALDH1A1; b) contain a (radio)fluorine atom that would allow for detection via gamma counting/PET imaging; c) a suitable hydrophobic‐hydrophilic balance which would allow for passive diffusion in and out of cells, and importantly; d) subsequent trapping of the in situ generated carboxylic acid product within the cytosol as a result of the acquired negative charge (Figure 1 A). We took a substrate‐based approach for the imaging of ALDH1A1 to provide a functional readout of enzymatic activity. Substrate‐based radiotracers provide an advantage over radiolabeled inhibitors which only report on enzyme expression. Moreover, multiple substrate molecules can be turned over by a single enzyme, thereby increasing the sensitivity of detection when compared to radiolabeled inhibitors.

The starting point for our small molecule probe development was *N*,*N*‐diethylaminobenzaldehyde (DEAB) **1**, which is a well‐known inhibitor of ALDH1A1 (*K*_i_ values of 10–40 nm).^18^ Given the advantages of substrate‐based radiotracers, our initial goal was to convert **1** in to a substrate whilst maintaining suitable ALDH1A1 selectivity over other commonly expressed ALDH isozymes. **1** is thought to form a stalled acyl‐enzyme intermediate as a result of the delocalization of the electron lone pair of the *para*‐substituted amine into the aromatic ring.18a,18c To convert **1** from an inhibitor to a substrate, we uncoupled the amine nitrogen from the aromatic π‐system through the introduction of a methylene linker to give **2** (Figure 1 B), which was rapidly converted to the carboxylate product under the action of ALDH1A1 (Figure S1). In order to assess the enzyme kinetics of the compounds in this study, we examined the effect of substrate concentration on the initial enzyme velocity using recombinant human ALDH1A1, ALDH2 and ALDH3A1 enzymes, which are commonly expressed in human cancer. ALDH2 is expressed in the mitochondrial matrix and plays a critical role in alcohol metabolism,^19^ whereas ALDH3A1 is localized in both the nucleus and cytosol and functions to detoxify aldehydes formed during UV‐induced lipid peroxidation.^20^ Furthermore, these three isoforms exhibit three different rate‐limiting steps: ALDH1A1′s being the cofactor dissociation,^21^ the deacylation‐step for ALDH2,^22^ and hydride transfer for ALDH3A1.^23^

**Scheme 1:**
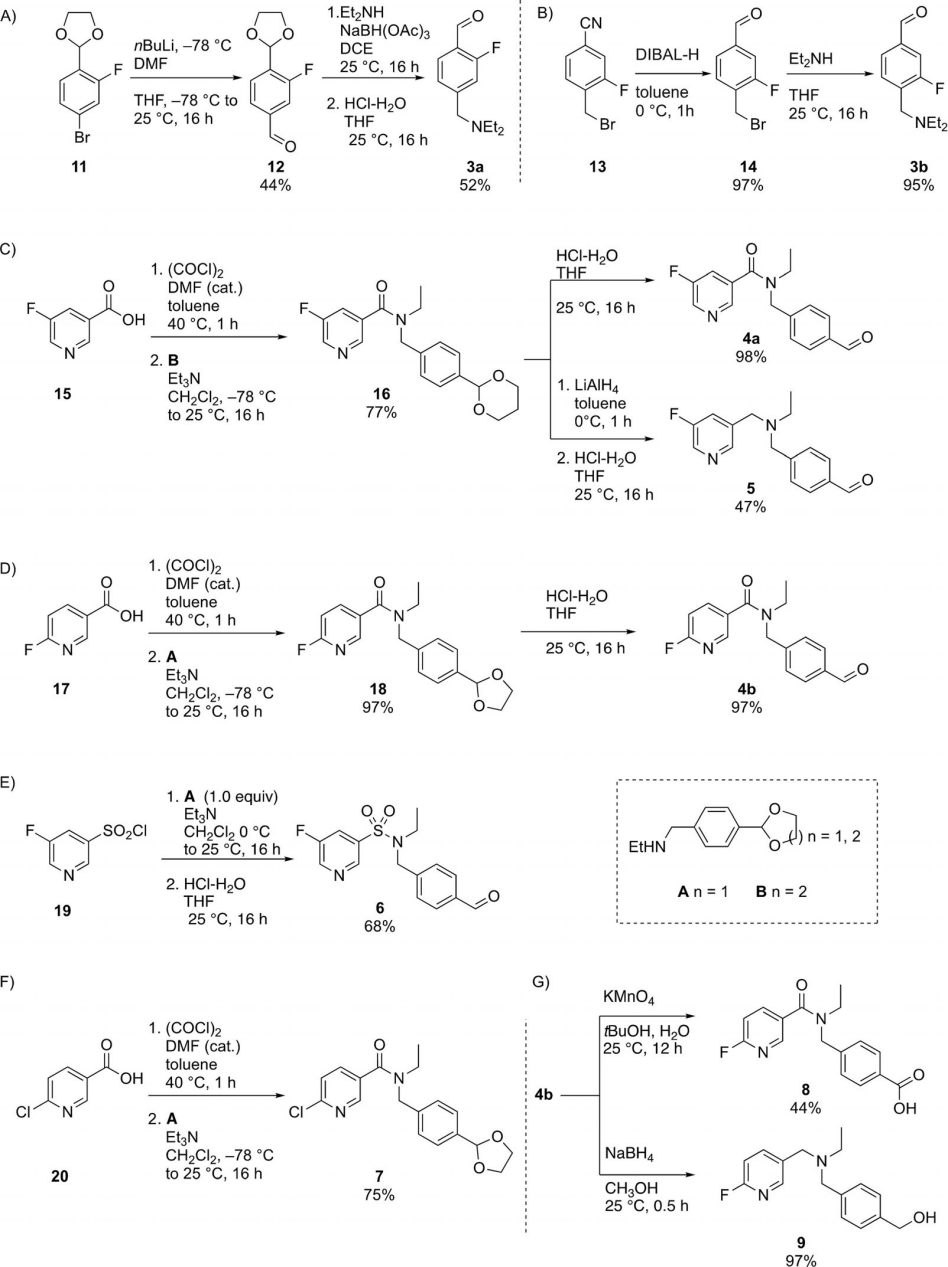
Synthetic route to **3 a**‐**b**,**4 a**‐**b**, **5**, **6**, **7**, **8** and **9**. All yields are isolated yields. See Supporting Information for further details and for the synthesis of **A** and **B**.

We made a single‐point modification to **2**, with the addition of a fluorine to the aromatic ring—an essential requirement for ^18^F‐radiofluorine‐based radiotracers—to give compounds **3 a** and **3 b**. The lithium/bromine exchange on **11** followed by quenching with DMF afforded aldehyde **12**. Stirring **12** with NaBH(OAc)_3_ and diethylamine in DCE afforded the crude reductive amination product which was directly treated with aqueous hydrochloric acid in THF to furnish **3 a** (Scheme 1 A). Reduction of nitrile **13** with diisobutylaluminium hydride cleanly produced aldehyde **14** which was thereafter stirred with excess diethylamine in THF to yield aldehyde **3 b** (Scheme 1 B). Amide **16** was prepared by reacting the acid chloride of 5‐fluoronicotinic acid **15** with amine **B** (Scheme 1 C). The acid‐catalyzed cleavage of the acetal furnished compound **4 a**. LiAlH_4_ reduction of the amide bond in **16** followed by the acid‐catalyzed acetal cleavage afforded amine **5** (Scheme 1 C). In a similar manner, the acid chloride of 6‐fluronicotinic acid **16** was reacted with amine **A** to yield amide **18** which following an acid‐catalyzed acetal cleavage furnished aldehyde **4 b**. The sulfonamide **6** was accessed by reacting the commercially available 5‐fluoropyridine‐3‐sulfonyl chloride **19** with amine **A** following an acid‐catalyzed acetal cleavage (Scheme 1 E).

**Figure 2:**
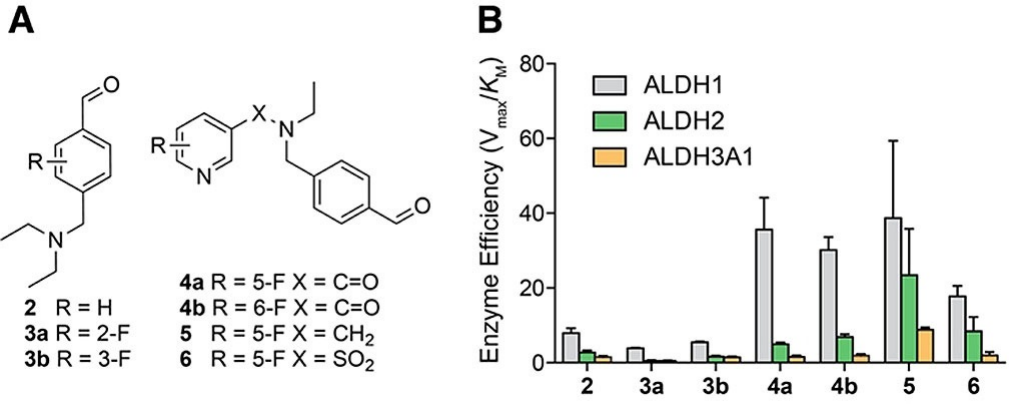
Structure‐activity relationship. A) Chemical structures for the compounds in this study. B) Enzyme efficiency of human recombinant ALDH1A1, ALDH2 and ALDH3A1 for compounds **2**–**6**. The “enzyme efficiency” (V_max_/*K*_M_) provides a measure of how efficiently an enzyme converts a substrate into product. Note: see Scheme 1 and Supporting Information for chemical synthesis of compounds **2**–**6** and Figure S2 for Michaelis Menten plots used to derive enzyme kinetic data.

The benzylic amine **2** showed a higher affinity (lower *K*_M_) and catalytic efficiency (V_max_/*K*_M_) for ALDH1A1 over both ALDH2 and ALDH3A1 (Table 1, entry 1 and Figure 2 B, respectively), indicating that the isozyme selectivity of DEAB was maintained. Interestingly, **3 a** and **3 b** exhibited lower affinity for ALDH1A1 than the non‐fluorinated analogue 2, with a *K*_M_ of 0.28±0.12 mm, 0.26±0.08 mm and 0.16±0.03 mm, respectively (Table 1, entries 1–3).

**Table 1:** Kinetic properties of human ALDH1A1, ALDH2 and ALDH3A1 towards oxidation of aldehydes in this study.

Entry	Com‐	ALDH1A1			ALDH2			ALDH3A1		
	pound	*V*_max_ [nmol min^−1^ μg^−1^]	*K*_M_ [mm]	*V*_max_/*K*_M_	*V*_max_ [nmol min^−1^ μg^−1^]	*K*_M_ [mm]	*V*_max_/*K*_M_	*V*_max_ [nmol min^−1^ μg^−1^]	*K*_M_ [mm]	*V*_max_/*K*_M_
**1**	**2**	1.22±0.09	0.16±0.03	7.9	0.09±0.01	0.03±0.01	2.7	2.81±0.03	1.87±0.06	1.5
**2**	**3 a**	1.10±0.18	0.28±0.12	3.9	0.30±0.02	0.64±0.12	0.47	0.89±0.03	1.78±0.11	0.5
**3**	**3 b**	1.42±0.17	0.26±0.08	5.5	0.19±0.01	0.12±0.01	1.5	1.51±0.10	0.99±0.13	1.5
**4**	**4 a**	1.08±0.07	0.03±0.01	33.9	0.67±0.01	0.13±0.01	4.9	1.87±0.03	1.19±0.06	1.5
**5**	**4 b**	0.98±0.02	0.03±0.01	28.7	0.53±0.01	0.07±0.01	6.8	2.22±0.18	1.10±0.18	2.0
**6**	**5**	1.36±0.08	0.04±0.01	33.3	0.32±0.02	0.02±0.01	19.6	1.6±0.13	0.18±0.03	8.7
**7**	**6**	1.5±2.68	0.10±0.24	14.6	0.27±0.01	0.03±0.01	7.4	0.03±0.01	0.02±0.01	1.7

Given that fluorination proximal to the aldehyde moiety decreased affinity for ALDH1A1, we next explored compounds with fluorine atoms that were remote from the aldehyde. Compounds **4 a** and **4 b** exhibited a five‐fold increase in ALDH1A1 affinity over **2** (Table 1, entries 1, 4 and 5). Furthermore, the enzyme efficiency for **4 a** was 7‐fold higher for ALDH1A1 than ALDH2, with the enzyme efficiency for ALDH1A1 over 20‐fold higher with respect to ALDH3A1; that is, the linking of the pyridine via an amide bond resulted in improved selectivity for ALDH1A1 (Figure 2 B; Table 1, entry 4). The position of the fluorine on the pyridine ring crucially did not play a key role in substrate kinetics, as seen with amide **4 b** which exhibited analogous behavior to **4 a**, albeit with marginally decreased ALDH1A1 enzyme selectivity (Table 1, entry 5). In the absence of the amide linkage, the tertiary amine **5**, whilst exhibiting a similar ALDH1A1 binding profile as compounds **4 a** and **4 b**, was readily oxidized by ALDH2 when compared to the other compounds tested (Figure 2 B; Table 1, entry 6). Replacing the amide of compound **4 a** with a sulfonamide gave us compound **6** with diminished ALDH1A1 enzyme efficiency when compared to **4 a** (Figure 2 B; Table 1, entries 5 and 7). In summary, we have shown **4 a**, **4 b** and **5** to be excellent substrates for ALDH1A1, with **4 a** and **4 b** to have good ALDH1A1 selectivity over the other isoforms tested (Figure 2 B).

**Figure 3:**
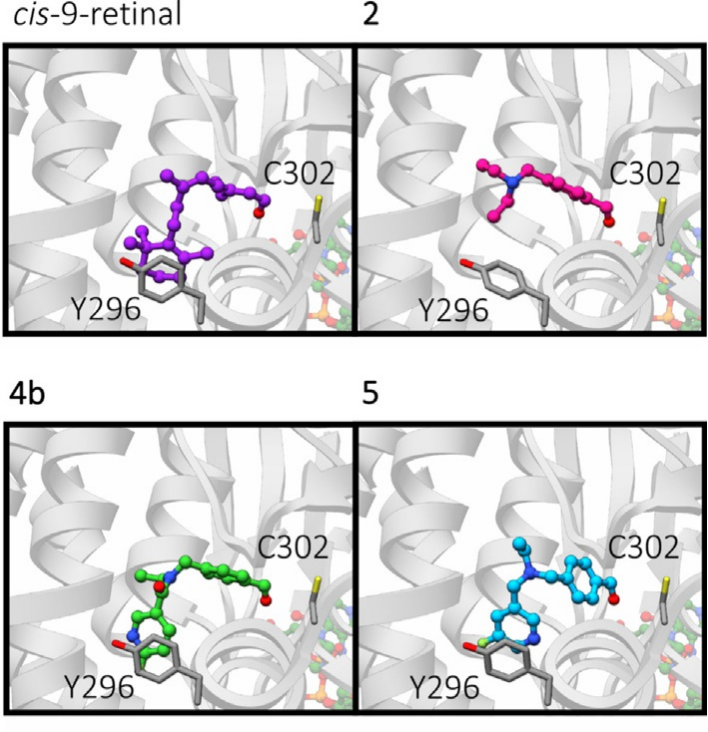
In silico modeling. Predicted binding modes of ALDH1A1 substrates from in silico docking studies. Predicted binding modes in ALDH1A1 of 9‐*cis*‐retinal (purple), **2** (magenta), **4 b** (green) and **5** (cyan), shown in ball and stick representation. Catalytic residue C302, and Y296 which was identified in π‐stacking interactions with the substrates are shown as dark grey sticks. Ribbons are shown in grey and faded for clarity, the cofactor NAD(H) is shown in pastel orange, green, red and blue ball and stick representation.

To understand why compounds **4 a** and **4 b** exhibited enhanced selectivity for ALDH1A1 over the other isozymes tested, we carried out an in silico docking study wherein compounds **2**–**6**, and the natural ALDH1A1 substrates 9‐*cis*‐retinal, 13‐*cis*‐retinal, and all‐*trans*‐retinal, were docked into protein structures for ALDH1A1, ALDH2, and ALDH3A1 (Protein data bank ID: 4WB9, 1O01 and 4L2O respectively). In comparison to ALDH2 and ALDH3A1, ALDH1A1 has the largest access tunnel to the active site residue (Figure S3), which suggests that bulkier and more rigid substrates would be preferentially turned over by ALDH1A1. Tunnel topography may also explain why reducing the amide in compound **4 a** to a flexible tertiary amine in compound **5**, resulted in increased affinity for ALDH2 and ALDH3A1. Compounds **4 a** and **4 b** displayed similar binding modes as **2** to ALDH1A1 in our in silico model, as shown in Figure 3. The benzaldehyde portions of compounds **4 a**, **4 b**, **5**, and **6** occupied a similar site to that of compound **2**; however, the pyridyl‐ring was predicted to make additional contacts, presumably via π‐stacking between the pyridine and the tyrosine‐Y296 of ALDH1A1 (Figure 3). This putative π‐stacking interaction may explain the increase in binding affinity for compounds **4 a**, **4 b**, **5**, and **6** when compared to compounds that lack the pyridine functional group. The low binding affinity of compounds **4 a**, **4 b**, **5**, and **6** for ALDH2 (Figure 2 and Table 1) might be ascribed to the equivalent phenylalanine residue within the active site, which being less electron‐rich than tyrosine, results in a lowered π‐stacking efficiency.^24^ In ALDH3A1 the active site tyrosine is replaced by a methionine, which is incapable of π‐stacking. Consequently, no pattern in binding affinity for ALDH3A1 was observed for substrates with the pyridyl substituent.

**Scheme 2:**
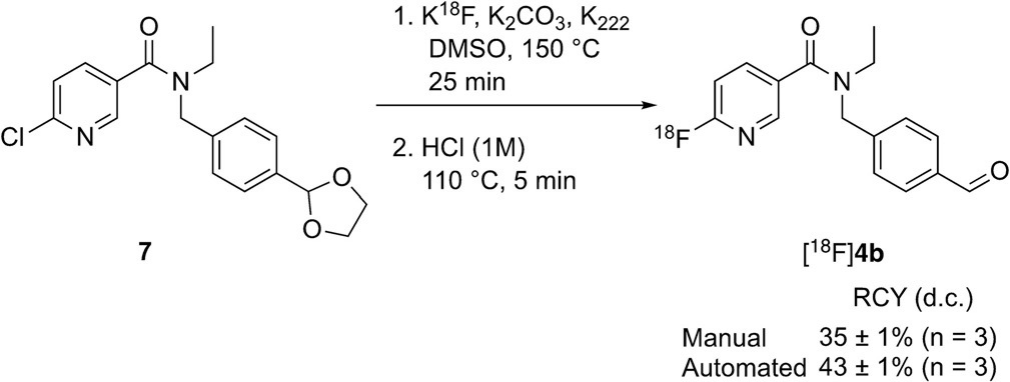
Radiofluorination of [^18^F]**4 b**. See Supporting Information for further details. RCY, radiochemical yield; d.c.=decay‐corrected.

Compound **4 b** was further considered as a potential radiochemical probe to assess cellular ALDH activity as it not only had excellent enzyme efficiencies and selectivity for ALDH1A1, but also potential radiochemical accessibility. Radiolabeling via nucleophilic substitution with [^18^F]fluoride allows for substitution at the 2‐position on a pyridine ring, whilst the 3‐position **4 a** exhibits poor reactivity.^25^ Consequently, the ^18^F‐radiolabeled analogue of **4 b**, *N*‐ethyl‐6‐(fluoro‐^18^F)‐*N*‐(4‐formylbenzyl)nicotinamide was chosen as our lead candidate radiotracer. To access [^18^F]**4 b**, a nucleophilic aromatic substitution (S_N_Ar) was performed on the 6‐chloronicotinamide precursor **7**, which was prepared from the acid chloride of 6‐chloronicotinic acid **20** and amine **A** (Scheme 1 F). Stirring **7** with [^18^F]KF/K_222_ in DMSO at 150 °C for 25 min followed by an acid‐catalyzed acetal cleavage step furnished [^18^F]**4 b** in 35±1 % (*n*=3) decay‐corrected radiochemical yield (RCY) after reverse phase semi‐preparative HPLC purification following manual radiolabeling (Scheme 2). Automation of this procedure improved the RCY to 43±1 % (decay‐corrected; *n*=3). Starting the synthesis with ≈1.0 GBq of [^18^F]fluoride, the radiotracer was obtained with a radiochemical purity of 99 % (see Supporting Information) and a molar activity of up to 4.4 GBq μmol^−1^ (manual) and up to 7.2 GBq μmol^−1^ (automated).

**Figure 4:**
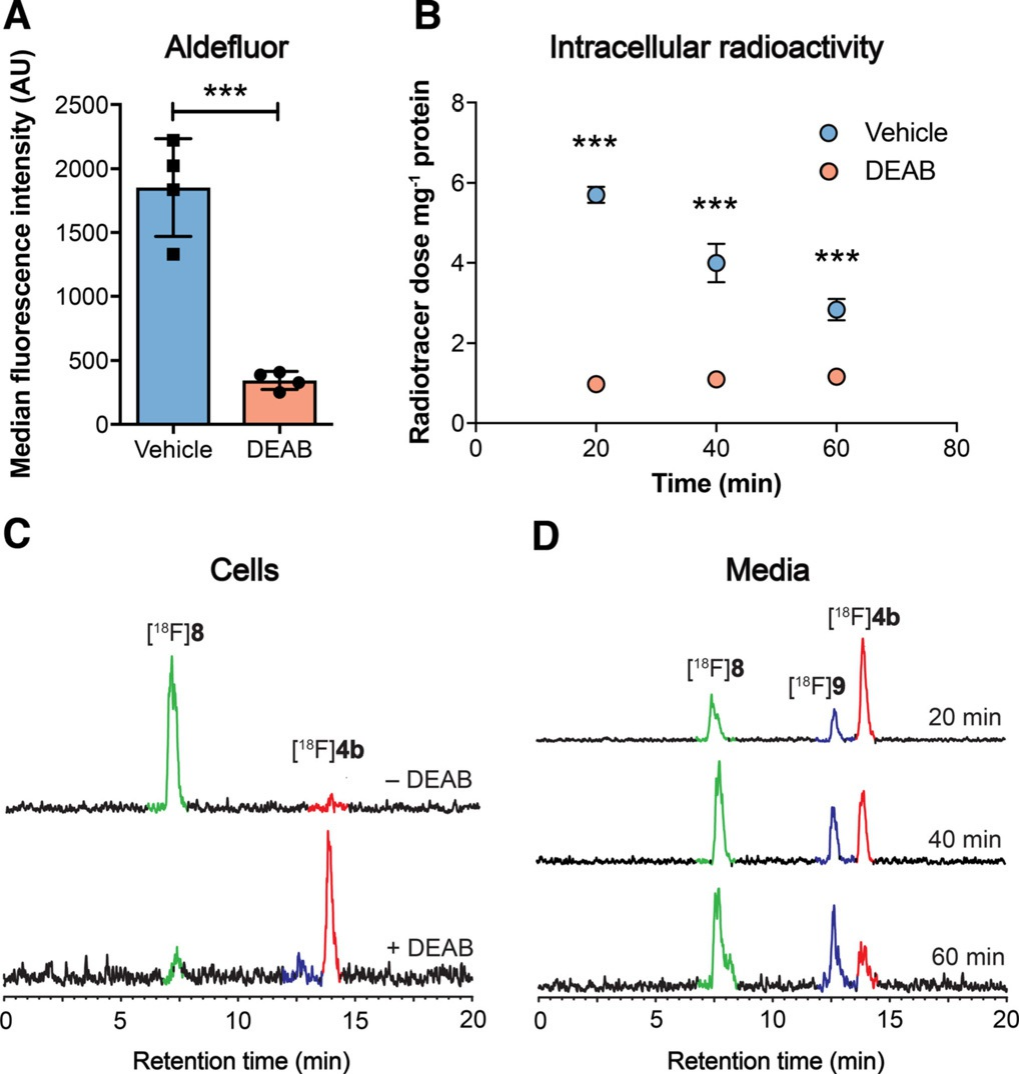
Detection of ALDH activity in HCT116 mut cells. A) ALDH activity in vehicle (DMSO) and DEAB‐treated cells (30 μm, 45 min), as measured by ALDH‐mediated trapping of Aldefluor and detection by flow cytometry. Data are means ±SD (*n*=4). B) Intracellular radioactivity levels in HCT116 mut cells treated with either vehicle or DEAB (30 μm, 45 min) following incubation with [^18^F]**4 b**. Data are means ±SD (*n*=3). C) Radio‐HPLC chromatograms from cell lysates following 20 min incubation of [^18^F]**4 b** (red peak) with (bottom), or without DEAB treatment (top). The green peak corresponds to the carboxylate [^18^F]**8**. D) Composition of radioactive metabolites in the media following 20, 40 and 60 min incubation of [^18^F]**4 b** (red) with HCT116 mut cells. The presence of [^18^F]**8** (green peak) along with the alcohol [^18^F]**9** (blue peak) were identified via co‐injection with [^19^F]**4 b**, [^19^F]**8**, and [^19^F]**9** and their detection at 254 nm (see Supporting Information for further information). ***, *P*<0.001 in vehicle vs. DEAB‐treated cells.

Furthermore, reference compounds for cellular metabolite analysis, carboxylic acid **8** and alcohol **9**, were prepared by the KMnO_4_ mediated oxidation of **4 b**, and the NaBH_4_ mediated reduction of aldehdye **4 b**, respectively (Scheme 1 G). With a tracer candidate in hand we assessed whether [^18^F]**4 b** could provide a readout of ALDH activity in tumor cells grown in culture. We used the HCT116 *Kras* ^G13D/−^ mutant (HCT116 mut) human colorectal cancer cell line as a model of aggressive, therapy‐resistant cancer. HCT116 mut lines displayed high ALDH activity, which was specifically inhibited through the incubation of cells with the ALDH inhibitor DEAB (30 μm; Figure 4 A). Incubation of HCT116 mut cells with [^18^F]**4 b** resulted in rapid cell uptake and intracellular retention of radioactivity, reaching 5.7±0.2 % radiotracer dose mg^−1^ protein at 20 min (*n*=3). Treatment of cells with DEAB resulted in an 83 % reduction in cell‐associated radioactivity to 1.0±0.1 % radiotracer dose mg^−1^ protein (*n*=3; *P*<0.0001), indicating ALDH‐specific intracellular trapping of either [^18^F]**4 b** or its products (Figure 4 B). To confirm the identity of the intracellular radioactive species present, we performed radio‐HPLC analysis of the resulting cell lysates. 20 min after the addition of the radiotracer, near‐complete conversion of the [^18^F]**4 b** aldehyde to the corresponding carboxylic acid [^18^F]**8** was observed (Figure 4 C; see Scheme 1 G for structures of **8** and **9**), confirmed through co‐injection with non‐radioactive [^19^F]**8** (See Supporting Information for details). Incubation of cells with DEAB resulted in a substantial reduction in the production of [^18^F]**8**, with >90 % of radioactivity present as the parent compound (Figure 4 C), suggesting that in the absence of ALDH1A1 activity the aldehyde does not undergo intracellular oxidation and therefore is not retained.

Whilst the levels of [^18^F]**4 b** in DEAB‐treated cells did not significantly change over the 60 min time course of the experiment (*P*>0.05; Figure 4 B, red circles), the amount of intracellular radioactivity decreased in a time‐dependent fashion in vehicle (DMSO)‐treated cells following addition of [^18^F]**4 b** (Figure 4 B, blue circles). Despite washout of radioactivity from vehicle‐treated cells, cell‐associated radioactivity at 60 minutes remained 2.4‐fold higher than cells treated with DEAB, at 2.8±0.3 % radiotracer dose mg^−1^ and 1.2±0.1 % radiotracer dose mg^−1^ protein, respectively (*P*=0.0005; *n*=3). Analysis of media samples by radio‐HPLC following cell incubation showed a progressive increase in the levels of [^18^F]**8** and the subsequent appearance of the alcohol [^18^F]**9** (see Scheme 1 G for structures), indicating imperfect intracellular trapping of [^18^F]**8** following its production by ALDH (Figure 4 D). Appearance of [^18^F]**8** in the media therefore accounts for the time‐dependent reduction in cell‐associated radioactivity observed in vehicle‐treated cells following incubation with [^18^F]**4 b**, potentially as the result of efflux pump‐mediated excretion of the carboxylate. Media incubation of [^18^F]**4 b** at 37 °C for 60 min in the absence of cells did not result in the production of [^18^F]**8** (Supporting Information; chromatogram S3), confirming that the conversion to the carboxylate was cell‐mediated. Together, these data show [^18^F]**4 b** to be specific and sensitive marker of ALDH activity in tumor cells.

**Figure 5:**
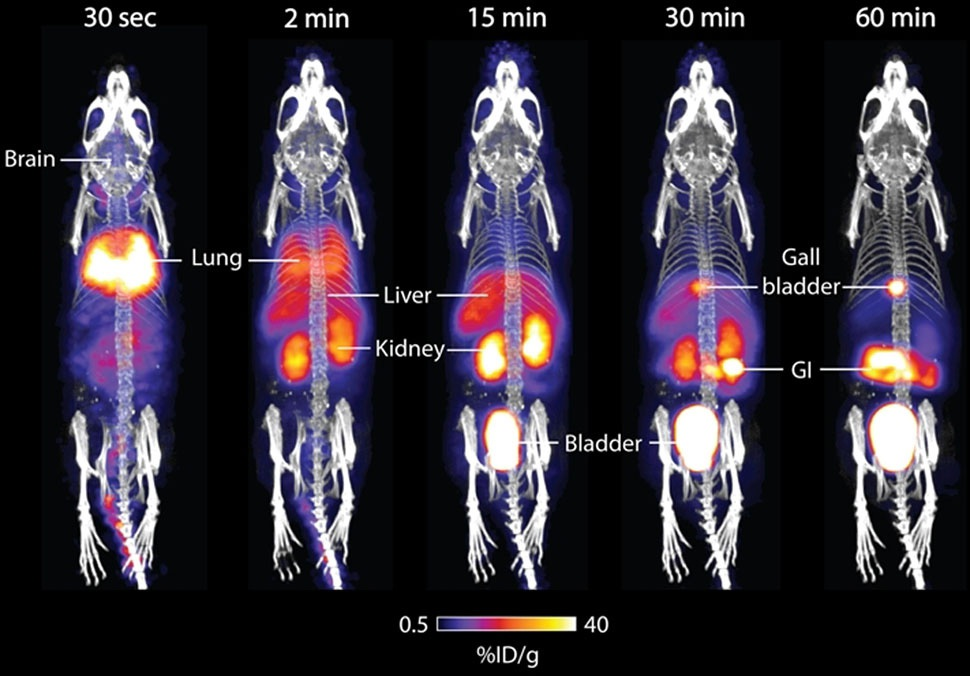
PET/CT maximum intensity projections of [^18^F]**4 b** in a healthy balb/c mouse. Representative time course images are shown to illustrate [^18^F]**4 b** pharmacokinetics. Gray scale is CT image, and color scale is PET image. Uptake in key organs are illustrated. GI, gastrointestinal tract. Images are representative of three separate mice.

Given that [^18^F]**4 b** can measure ALDH activity in HCT116 mut cells with high sensitivity and specificity, we next explored [^18^F]**4 b's** in vivo biodistribution. Dynamic microPET imaging following intravenous injection of [^18^F]**4 b** into healthy balb/c mice (Figure 5) revealed rapid and extensive uptake in the lungs, known to express high levels of ALDH1A1. Liver uptake peaked at 5 min post injection (p.i.) at 20.0±2.6 % injected dose (ID) g^−1^ tissue (*n*=3 mice). [^18^F]**4 b** clearance was initially via the kidneys and bladder to afford high contrast images with low uptake in background tissue (2.2±0.3 %ID g^−1^ in the muscle at 5 min p.i.; *n*=3 mice). However, hepatobiliary excretion was evident by 30 min p.i., as shown by radiotracer uptake in the gallbladder and gastrointestinal (GI) tract, indicating possible metabolism of the parent compound at this time point. [^18^F]**4 b** was further cleared from all other organs other than gallbladder and GI over the remainder of the imaging time course. For time activity curves for organs with high [^18^F]**4 b** uptake, see Figure S4.

The combined renal and hepatobiliary excretion observed at 30 min p.i., prompted us to assess the in vivo stability of [^18^F]**4 b**. Radio‐HPLC analysis of plasma taken by terminal exsanguination from anesthetized mice following tail vein intravenous (i.v.) injection of [^18^F]**4 b** revealed complete conversion to [^18^F]**8** by 2 min (Supporting Information; chromatogram S7). The appearance of a second, unknown peak of similar retention time to [^18^F]**8** (Supporting Information chromatogram S7) was evident by 5 min, increasing to ≈40 % total radioactivity in the blood by 60 min, which may account for the mixed routes of excretion observed at later imaging time points. Importantly, the rapid metabolism of the free aldehyde may limit the in vivo tissue penetration of [^18^F]**4 b**.

## Conclusions

In conclusion, we have developed a focused library of ALDH substrates based on the well‐known inhibitor DEAB. The addition of a fluoronicotinamide dramatically increased ALDH1A1 isoform specificity, thought to result from π‐stacking interactions with a tyrosine residue proximal to the active site. *N*‐Ethyl‐6‐(fluoro)‐*N*‐(4‐formylbenzyl)nicotinamide **4 b** was taken forward for radiolabeling and evaluation in both tumor cells and in mice. [^18^F]**4 b** was rapidly taken up by ALDH‐expressing colorectal cancer cells in culture and intracellularly trapped through ALDH‐specific conversion to the corresponding carboxylic acid. In vivo, high radiotracer uptake in the lung and liver were observed, combined with rapid clearance from background tissues and excretion via the urinary tract. Rapid oxidation of this lead compound in vivo however highlights a potential limitation of aldehyde‐based radiotracers for ALDH imaging. Future strategies will focus on the development of second generation ALDH1A1 radiotracers with improved in vivo stability to image drug resistance in animal models of cancer.

## Experimental Section

See Supporting Information for detailed synthetic, radiochemical methods, enzyme assays, cellular and studies in mice.

## Conflict of interest

The authors declare no conflict of interest.

## Supporting information

As a service to our authors and readers, this journal provides supporting information supplied by the authors. Such materials are peer reviewed and may be re‐organized for online delivery, but are not copy‐edited or typeset. Technical support issues arising from supporting information (other than missing files) should be addressed to the authors.

Supplementary
