# Supplementary material for: Mapping Aldehyde Dehydrogenase 1A1 Activity using an [18F]Substrate‐Based Approach
Source: Chemistry. 2019 Jan 14;25(9):2345–51. doi: 10.1002/chem.201805473 (PMC6379060; doi:10.1002/chem.201805473)
Supplement: Supplementary file 1 — Supplementary [file CHEM-25-2345-s001.pdf]

CHEMISTRY
A European Journal

Supporting Information

Mapping Aldehyde Dehydrogenase 1A1 Activity using an [^{18}F]Substrate-Based Approach

Raul Pereira,[a] Thibault Gendron,[b] Chandan Sanghera,[a] Hannah E. Greenwood,[a] Joseph Newcombe,[b, c] Patrick N. McCormick,[a] Kerstin Sander,[b] Maya Topf,[c] Erik Årstad,[b] and Timothy H. Witney*[a]

chem_201805473_sm_miscellaneous_information.pdf

GENERAL 2
SYNTHESIS 3
RADIOCHEMISTRY 14
ENZYME KINETICS 19
DOCKING STUDIES 20
CELL ASSAYS 22
PET SCANS 23
BLOOD METABOLITE ANALYSIS 23
SUPPLEMENTAL FIGURES 24
HPLC CHROMATOGRAMS 27
NMR SPECTRA 32
REFERENCES 54

GENERAL

All reactions, unless specified, were performed in dried apparatus with magnetic stirring under an inert atmosphere of argon. All solvents and chemicals were used as purchased unless stated otherwise. All NMR spectra were recorded on Bruker AV300, AV400, AVIIIHD 500, AVIIIHD 600 or AVIIIHD 700 spectrometers. ^{1}H and ^{13}C NMR spectra are reported as chemical shifts (δ) in parts per million (ppm) relative to residual unlabelled solvent peak using the Bruker internal referencing procedure (edlock). ^{19}F NMR spectra are referenced relative to $CFCl_3$. Coupling constants (J) are reported in units of hertz (Hz) and are rounded off. The following abbreviations are used to describe multiplets: s (singlet), d (doublet), t (triplet), q (quartet), m (multiplet), brs (broad). High-resolution mass spectra (HRMS, m/z) were recorded on Thermo Finingan MAT900 magnetic sector (EI), Agilent 6510 QTOF or Waters LCT Premier spectrometers using positive electrospray ionization (ESI). Infrared spectra were recorded as neat compounds using a Bruker Tensor 27 FT-IR spectrometer. Absorptions are reported in wavenumbers (cm^{-1}) and only peaks of interest are reported. Melting points of solids were measured on a Sanyo apparatus and are uncorrected. IUPAC names were generated using ChemDraw Professional 16.0. All dry solvents were bought either from Sigma or Acros and used as is. Thin layer chromatography (TLC) was performed using Merck aluminium-foil baked plates coated with Kieselgel 60 F245. The products were visualized using UV fluorescence (254 nm) or potassium permanganate stain. Flash chromatography was performed using an IsoleraTM flash system from Biotage using pre-packed silica gel (20-40 µm) columns using silica gel amount eluent systems as described for each experiment. 5-fluoronicotinic acid, 6-chloronicotinic acid, 6-fluoronicotinic acid, and 2-(4-bromophenyl)-1,3-dioxolane were purchased from Acros. Ethylamine hydrochloride and triethyl amine were purchased from Sigma-Aldrich. 5-Fluoropyridine-3-sulfonyl chloride was purchased from Insight BioTechnology.

SYNTHESIS

Compounds **2**,[1] **11**,[2] **13**,[3] 4-(1,3-dioxolan-2-yl)benzaldehyde,[4] and 4-(1,3-dioxan-2-yl)benzaldehyde[5] were prepared according to literature procedures.

4-((diethylamino)methyl)-2-fluorobenzaldehyde **3a**

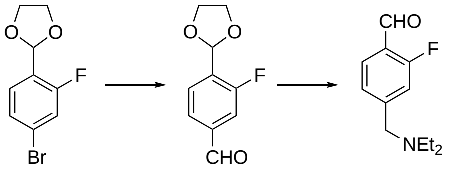

11 **12** **3a**

4-(1,3-dioxolan-2-yl)-3-fluorobenzaldehyde 12

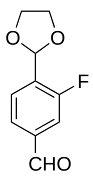

A dry 100 mL round-bottom flask was charged with 2-(4-bromo-2-fluorophenyl)-1,3-dioxolane, **11**, [2] (0.74 g, 3.0 mmol), evacuated and refilled with argon (3×). Dry THF (15 mL) was injected and the resulting solution was cooled to -78°C. *n*BuLi (2.8 mL, 1.6M in hexane, 4.5 mmol, 1.5 equiv) was injected over 5 minutes, and stirred at –78 °C for a further 1 hour after which dry DMF (0.35 mL, 4.5 mmol, 1.5 equiv) was injected and then the reaction was allowed to reach room temperature overnight. The reaction was quenched with water (15 mL) and extracted with ethyl acetate (50 mL × 3). The combined ether phases were rinsed with water (30 mL), brine (30 mL) and then dried over $MgSO_4$. The organic solvents were removed under reduced pressure to yield a yellow oil. Purification *via* flash chromatography [silica gel (24 g), ethyl acetate/petroleum ether (0:100) to (10:90)] to yield 4-(1,3-dioxolan-2-yl)-3-fluorobenzaldehyde, **12**, as a clear oil (0.26 g, 44%).

^{19}F NMR (282 MHz, $CDCl_3$): δ [ppm]= –117.3. **^{1}H NMR** (300 MHz, $CDCl_3$): δ [ppm]= 4.12 (m, 4H, OCH_2CH_2O), 6.12 (s, 1H, OC*H*O), 7.58 (d, *J* = 9.9 Hz, 1H, Ar-*H*), 7.71 (m, 2H, Ar-*H*), 9.99 (d, *J* = 1.7 Hz, 1H, C*H*O). **^{13}C NMR** (75 MHz, $CDCl_3$): δ [ppm]= 65.7, 98.5 (d, *J* = 3.3 Hz), 115.8 (d, *J* = 22.0 Hz), 125.9 (d, *J* = 3.5 Hz), 128.7 (d, *J* = 3.8 Hz), 131.8 (d, *J* = 13.2 Hz), 138.8 (d, *J* = 6.6 Hz), 161.5 (d, *J* = 252.9 Hz), 190.7 (d, *J* = 2.2 Hz). **IR** (ATR, neat): 1/λ [cm^{-1}] =2890, 1696, 1583, 1432, 1385, 1271, 1186, 1073, 941. **HRMS** (CI) m/z $[M]^{+\cdot}$: calcd. for $C_{10}H_9FO_3$: 196.0535, found:196.0530.

4-((diethylamino)methyl)-2-fluorobenzaldehyde 3a

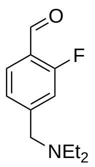

A 50 mL round-bottom flask was charged with 4-(1,3-dioxolan-2-yl)-3-fluorobenzaldehyde, **12**, (0.58 g, 3.0 mmol) and sodium triacetoxyborohydride (0.89 g, 4.2 mmol, 1.4 equiv), the flask was evacuated and refilled with argon (3×). diethylamine (0.22 g, 0.3 mL, 3.0 mmol, 1.0 equiv) and dry DCE (8 mL) were injected and the reaction was allowed to stir overnight at room temperature. The reaction was quenched with K_2CO_3 (sat. aq) until alkaline (pH~11). Phases separated, and the aqueous phase was extracted with diethyl ether (50 mL × 3). The combined organic phases were dried over $MgSO_4$ and the organic solvents were removed under reduced pressure to yield

N-(4-(1,3-dioxolan-2-yl)-3-fluorobenzyl)-*N*-ethylethanamine as a yellow oil that was dissolved in wet THF (10 mL) and added to a 50 mL round-bottom flask. Conc. HCl was added until acidic (pH~1), and the resulting solution was stirred overnight at room temperature. The reaction was quenched with aqueous K_2CO_3 (sat. aq) until alkaline (pH~11), and then extracted with methylene chloride (50 mL × 3). The combined organic phases were dried over $MgSO_4$ and the organic solvents were removed under reduced pressure to yield a yellow oil. Purification *via* flash chromatography [silica gel (24 g), methanol/methylene chloride (0:100 to 10:90)] to yield 4-((diethylamino)methyl)-2-fluorobenzaldehyde, **3a**, as a clear pale yellow oil (0.332 g, 52%).

^{19}F NMR (282 MHz, $CDCl_3$): δ [ppm]= – 122.5. **^{1}H NMR** (300 MHz, $CDCl_3$): δ [ppm]= 1.03 (t, *J* = 7.1 Hz, 6H, C*H*$_3$CH$_2$), 3.60 (q, *J* = 7.1 Hz, 4H, CH$_3$C*H*$_2$), 3.60 (s, 2H, Ar-C*H*$_2$-NEt$_2$) 7.23 (m, 2H, Ar-*H*), 7.79 (t, *J* = 7.5 Hz, 1H, Ar-*H*), 10.3 (s, 1H, C*H*O). **^{13}C NMR** (75 MHz, $CDCl_3$): δ [ppm]= 12.1, 47.3, 57.4 (d, *J* = 1.7 Hz), 116.2 (d, *J* = 20.9 Hz), 122.9 (d, *J* = 8.3 Hz), 124.6 (d, *J* = 3.1 Hz), 128.5 (d, *J* = 2.2 Hz), 151.6 (d, *J* = 8.2 Hz), 165.1 (d, *J* = 258.4 Hz), 187.2 (d, *J* = 6.3 Hz). **IR** (ATR, neat): 1/λ [cm^{-1}] =2968, 2931,2805, 1692, 1618, 1574, 1425, 1397, 1245, 1192, 1099. **HRMS** (ESI) m/z [M+H]$^+$: calcd. for $C_{12}H_{17}FNO$: 210.1294, found:210.1295.

4-((diethylamino)methyl)-3-fluorobenzaldehyde **3b**

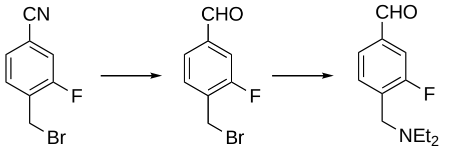

13 **14** **3b**

4-(bromomethyl)-3-fluorobenzaldehyde 14

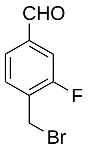

A dry 100 mL round-bottom flask was charged with 4-(bromomethyl)-3-fluorobenzonitrile, **13**,[3] (1.1 g, 5.14 mmol), evacuated and refilled with argon (3×). Dry toluene (25 mL) was injected and the resulting solution was cooled to 0 °C. Diisobutylaluminum hydride (7.7 mL, 1M in hexanes 7.7 mmol, 1.5 equiv) was injected over 5 minutes and then stirred for one hour more at 0 °C. The reaction was carefully quenched with HCl (25 mL, 10% aqueous) at 0 °C. The reaction was allowed to stir at room temperature for a further 15 minutes before being extracted with methylene chloride (50 mL × 3). The combined organic phases were dried over $MgSO_4$ and the organic solvents were removed under reduced pressure to yield a yellow oil. Purification *via* flash chromatography [silica gel (80 g), ethyl acetate / petroleum ether (0:100 to 15:90)] to yield 4-(bromomethyl)-3-fluorobenzaldehyde, **14**, as a clear oil (1.08 g, 97%).

^{19}F NMR (282 MHz, $CDCl_3$): δ [ppm]= –115.1. **^{1}H NMR** (300 MHz, $CDCl_3$): δ [ppm]= 4.52 (s, 2H, Ar-C*H*$_2$-Br), 7.57 (m, 2H, Ar-*H*), 7.66 (m, 1H, Ar- *H*), 9.97 (d, *J* = 1.7 Hz, 1H, C*H*O). **^{13}C NMR** (75 MHz, $CDCl_3$): δ [ppm]= 24.5 (d, *J* = 4.4 Hz), 115.9 (d, *J* = 22.1 Hz), 126.3 (d, *J* = 3.6 Hz), 131.9 (d, *J* = 15.1 Hz), 132.1 (d, *J*

= 3.0 Hz), 138.4 (d, *J* = 6.5 Hz), 160.9 (d, *J* = 253.3 Hz), 190.4 (d, *J* = 2.0 Hz). **IR** (ATR, neat): 1/λ [cm^{-1}] = 3062, 1688, 1575, 1497, 1429, 1393, 1313 1245, 1210, 1145, 1134, 1089, 970. **HRMS** (CI) m/z $[M]^{+\cdot}$: calcd. for C_8H_6BrFO: 215.9580, found:215.9581.

4-((diethylamino)methyl)-3-fluorobenzaldehyde 3b

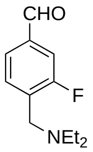

A dry 100 mL round-bottom flask was charged with 4-(bromomethyl)-3-fluorobenzaldehyde, **14**, (1.0 g, 4.6 mmol), evacuated and refilled with argon (3×). Dry THF (20 mL) followed by diethylamine (1.68 g, 2.4 mL, 23.0 mmol, 5.0 equiv) were injected, and then stirred overnight at room temperature. The reaction was quenched with water (10 mL) and then extracted with ethyl acetate (50 mL × 3). The combined organic phases were dried over $MgSO_4$ and the organic solvents were removed under reduced pressure to yield a yellow oil. Purification *via* flash chromatography [silica gel (40 g), ethyl acetate / petroleum ether (10:90 to 30:70)] to yield 4-((diethylamino)methyl)-3-fluorobenzaldehyde, **3b**, as a clear oil (0.96 g, >95%).

^{19}F NMR (282 MHz, $CDCl_3$): δ [ppm]= – 117.1 **^{1}H NMR** (300 MHz, $CDCl_3$): δ [ppm]= 1.05 (t, *J* = 7.1 Hz, 6H, CH_2CH_3), 2.55 (q, *J* = 7.1 Hz, 4H, CH_2CH_3), 3.67 (s, 2H, Ar-CH_2-NEt_2), 7.50 (dd, *J* = 9.7, 1.5 Hz, 1H, Ar-*H*), 7.66 (m, 2H, Ar-*H*), 9.95 (d, *J* = 1.7 Hz, 1H, C*H*O). **^{13}C NMR** (75 MHz, $CDCl_3$): δ [ppm]= 12.0, 47.4, 50.3 (d, *J* = 2.4 Hz), 115.2 (d, *J* = 23.0 Hz), 125.9 (d, *J* = 3.2 Hz), 131.6 (d, *J* = 4.7 Hz), 134.9 (d, *J* = 14.8 Hz), 136.9 (d, *J* = 6.6 Hz), 161.5 (d, *J* = 248.7 Hz), 190.9 (d, *J* = 2.2 Hz). **IR** (ATR, neat): 1/λ [cm^{-1}] =2968, 2931, 2805, 1692, 1617, 1574, 1425, 1397, 1245, 1192, 1099. **HRMS** (ESI) m/z $[M+H]^+$: calcd. for $C_{12}H_{17}FNO$: 210.1294, found: 210.1093.

N-(4-(1,3-dioxolan-2-yl)benzyl)ethanamine A

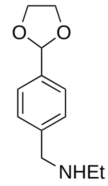

A 100 mL round bottom flask was charged with 4-(1,3-dioxolan-2-yl)benzaldehyde[4] (2.6 g, 14.6 mmol), $EtNH_2$.HCl (4.8 g, 58.4 mmol, 4.0 equiv), 4 Å molecular sieves (5 g), evacuated and refilled with argon (3×). Dry methanol (40 mL) followed by Et_3N (8.3 mL, 59.7 mmol, 4.0 equiv) was injected, and then stirred at room temperature for 2 hours after which $NaBH_4$ (0.56 g, 14.9 mmol, 1.0 equiv) was added portion-wise under argon. The reaction was allowed to stir overnight. The reaction was diluted with methylene chloride (100 mL) and filtered over a plug of celite (2 cm × 2 cm). The organic solvents were removed under reduced pressure to yield a white residue that was treated with K_2CO_3 (10 mL, sat. aq), water (20 mL) and then extracted with methylene chloride (100 mL × 3). The combined organic phases were rinsed with water (50 mL), brine (50 mL) and then dried over $MgSO_4$. The solvents were removed under reduced pressure to yield a yellow oil. Purification *via* flash chromatography [silica gel (80 g), methanol/methylene chloride (0:100) to (5:95)] to yield *N*-(4-(1,3-dioxolan-2-yl)benzyl)ethanamine as a clear oil (2.15 g, 71%).

^{1}**H NMR** (600 MHz, $CDCl_3$): δ [ppm]= 1.12 (t, *J* = 7.1 Hz, 3H, CH_3CH_2), 2.66 (q, *J* = 7.1 Hz, 2H, CH_3CH_2), 3.80 (s, 2H, Ar-CH_2-NHEt), 4.07 (m, 4H, OCH_2CH_2O), 5.80 (s, 1H, OC*H*O), 7.33 (d, *J* = 7.9 Hz, 2H, Ar-*H*), 7.43 (d, *J* = 7.8 Hz, 2H, Ar-*H*). ^{13}**C NMR** (150 MHz, $CDCl_3$): δ [ppm]= 15.3, 43.6, 53.7, 65.4, 103.7, 126.6, 128.3, 136.6, 141.6. **IR** (ATR, neat): 1/λ [cm^{-1}] = 2964, 2884, 1384, 1219, 1077, 1020, 941. **HRMS** (ESI) m/z $[M+H]^+$: calcd. for $C_{12}H_{18}NO_2$: 208.1332, found:208.1331.

N-(4-(1,3-dioxan-2-yl)benzyl)ethanamine **B**

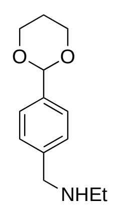

A 100 mL round bottom flask was charged with 4-(1,3-dioxan-2-yl)benzaldehyde[5] (0.7 g, 3.64 mmol), $EtNH_2$.HCl (0.59 g, 7.2 mmol, 2.0 equiv), 4 Å molecular sieves (2 g), evacuated and refilled with argon (3×). Dry methanol (20 mL) followed by Et_3N (1.1 mL, 7.28 mmol, 2.2 equiv) was injected, and then stirred at room temperature for 2 hours after which $NaBH_4$ (0.137 g, 3.64 mmol, 1.0 equiv) was added portion-wise under argon. The reaction was allowed to stir overnight. The reaction was diluted with methylene chloride (50 mL) and filtered over a plug of celite (2 cm × 2 cm). The organic solvents were removed under reduced pressure to yield a white residue that was treated with K_2CO_3 (5 mL, sat. aq), water (10 mL) and then extracted with methylene chloride (50 mL × 3). The combined organic phases were rinsed with water (25 mL), brine (25 mL) and then dried over $MgSO_4$. The solvents were removed under reduced pressure to yield a yellow oil. Purification *via* flash chromatography [silica gel (40 g), methanol/methylene chloride (0:100) to (5:95)] to yield *N*-(4-(1,3-dioxan-2-yl)benzyl)ethanamine as a clear oil (0.515 g, 64%).

^{1}**H NMR** (300 MHz, $CDCl_3$): δ [ppm]= 1.11 (t, *J* = 7.1 Hz, 3H,$NHCH_2CH_3$), 1.45 (m, 1H, OCH_2 CH_2CH_2O), 2.22 (m, 1H, OCH_2 CH_2CH_2O), 2.64 (q, *J* = 7.1 Hz, 2H, $NHCH_2CH_3$), 3.79 (s, 2H, Ar-CH_2-NHEt), 3.99 (m, 2H, OCH_2 CH_2CH_2O), 4.26 (m, 2H, OCH_2 CH_2CH_2O), 5.49 (s, 1H, OC*H*O), 7.32 (d, *J* = 8.0 Hz, 2H,Ar-*H*) 7.44 (d, *J* = 8.0 Hz, 2H, Ar-*H*). ^{13}**C NMR** (75 MHz, $CDCl_3$): δ [ppm]= 15.3, 25.9, 43.5, 53.7, 67.5, 101.7, 126.2, 128.2, 137.6, 141.0. **IR** (ATR, neat): 1/λ [cm^{-1}] = 2955, 2874, 1394, 1222, 1081, 1010, 934. **HRMS** (ESI) m/z $[M+H]^+$: calcd. for $C_{13}H_{20}NO_2$: 222.1494, found: 222.1490.

N-ethyl-5-fluoro-*N*-(4-formylbenzyl)nicotinamide **4a**

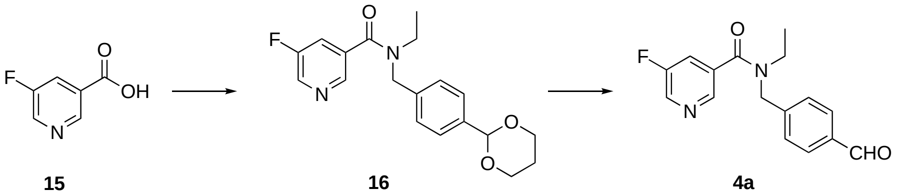

15 **16** **4a**

N-(4-(1,3-dioxan-2-yl)benzyl)-*N*-ethyl-5-fluoronicotinamide 16

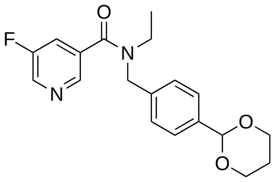

A dry 100 mL round-bottom flask was charged with 5-fluoronicotinic acid (0.423 g, 3.0 mmol), evacuated and refilled with argon (3×). Dry toluene (10 mL) followed by oxalyl chloride(0.380 mg, 0.26 mL, 1.0 equiv), and DMF (2 drops) were injected. The reaction was stirred at room temperature for 5 minutes and at 40 °C for a further hour. Dry methylene chloride (10 mL) was injected, and the reaction cooled to -78 °C. triethyl amine (1.7 mL, 12.0 mmol, 4.0 equiv) was injected followed by a solution of *N*-(4-(1,3-dioxan-2-yl)benzyl)ethanamine, **B**, (0.664 g, 3.0 mmol, 1.0 equiv) in dry methylene chloride (2 mL). The reaction was allowed to reach room temperature overnight, and then quenched with water (20 mL) and K_2CO_3 (10 mL, sat. aq), and then extracted with methylene chloride (50 mL × 3). The combined methylene chloride phases were rinsed with water (30 mL), brine (30 mL) and then dried over $MgSO_4$. The organic solvents were removed under reduced pressure to yield a yellow oil. Purification *via* flash chromatography [silica gel (40 g), methanol/methylene chloride (0:100) to (10:90)] to yield *N*-(4-(1,3-dioxan-2-yl)benzyl)-*N*-ethyl-5-fluoronicotinamide, **16**, as a clear oil (0.8 g, 77%).

^{19}F NMR (282 MHz, $CDCl_3$): δ [ppm]= –125.8. **^{1}H NMR*** (500 MHz, $CDCl_3$): δ [ppm]= 1.03-1.33 (m, 3H, C*H*$_3$CH$_2$), 1.39-1.43 (m, 1H, OCH$_2$C*H*$_2$CH$_2$O), 2.17 (m, 1H, OCH$_2$C*H*$_2$CH$_2$O), 3.13-3.48 (m, 2H, CH$_3$C*H*$_2$), 3.92-3.97 (m, 2H, OC*H*$_2$CH$_2$C*H*$_2$O), 4.22-4.24 (m, 2H, OC*H*$_2$CH$_2$C*H*$_2$O), 4.46-4.72 (m, 2H, Ar-C*H*$_2$), 5.47 (s, 1H, OC*H*O), 7.13 (brs, 1H, Ar-*H*), 7.23 (brs, 1H, Ar-*H*), 7.45 (m, 3H, Ar-*H*), 8.46 (m, 2H, Ar-*H*). **^{13}C NMR*** (125 MHz, $CDCl_3$): δ [ppm]= 12.1, 13.6, 25.7, 40.2, 42.7, 46.9, 51.8, 67.3, 101.0, 101.2, 121.2, 121.4, 126.3, 126.5, 126.7, 128.1, 133.4, 137.3, 138.6, 139.0, 139.1, 142.9, 143.2, 158.9 (d, J_{FC} = 259.6 Hz), 167.46, 167.47. **IR** (ATR, neat): 1/λ [cm^{-1}] =3072, 2964, 2931, 2845, 1624, 1573, 1481, 1415, 1379, 1317, 1273, 1236, 1214, 1152, 1104, 1009, 986, 946. **HRMS** (ESI) m/z [M+H]$^+$: calcd. for $C_{19}H_{22}FN_2O_3$: 345.1614, found:345.1617.

*Note: The compound exists as a mixture of rotamers.

N-ethyl-5-fluoro-*N*-(4-formylbenzyl)nicotinamide 4a

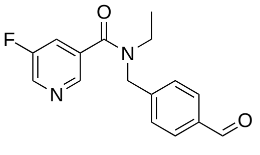

A 50 mL round-bottom flask was charged with *N*-(4-(1,3-dioxan-2-yl)benzyl)-*N*-ethyl-5-fluoronicotinamide, **16**, (0.76 g, 2.2 mmol) and THF (20 mL). To this solution, conc. HCl was added until acidic (pH~1), and the resulting solution was stirred overnight at room temperature. The reaction was quenched with aqueous K_2CO_3 (sat.) until alkaline (pH~11), and then extracted with methylene chloride (50 mL × 3). The combined organic phases were dried over $MgSO_4$ and the organic solvents were removed under reduced pressure to yield a yellow oil. Purification *via* flash chromatography [silica gel (40 g), methanol/methylene chloride (0:100) to (05:95)] to yield *N*-ethyl-5-fluoro-*N*-(4-formylbenzyl)nicotinamide, **4a**, as a clear oil (0.62 g, 98%).

^{19}F NMR (282 MHz, $CDCl_3$): δ [ppm]= –124.8. ^{1}H NMR* (600 MHz, $CDCl_3$) δ [ppm]= 1.14-1.24 (m, 3H, CH_3CH_2), 3.27-3.57 (m, 2H, CH_3CH_2), 4.59-4.83 (m, 2H, Ar-CH_2), 7-34-7.51 (m, 3H, Ar-*H*), 7.89 (m, 2H, Ar-*H*), 8.52 (brs, 2H, Ar-*H*), 10.01 (s, 1H, C*H*O). ^{13}C NMR* (150 MHz, $CDCl_3$) δ [ppm]= 12.4, 14.0, 40.9, 43.7, 47.6, 52.1, 121.6, 121.7, 127.2, 128.6, 130.4, 133.3, 136.0, 139.48, 139.64, 143.1, 143.13, 143.9, 159.1 (d, J_{FC} = 259.6 Hz), 167.9, 191.8. **IR** (ATR, neat): 1/λ [cm^{-1}] =3054, 2974, 1692, 1630, 1576, 1415, 1289, 1210, 1164, 891. **HRMS** (ESI) m/z $[M+H]^+$: calcd. for $C_{16}H_{16}FN_2FO_2$: 287.1196, found:287.1198.
*Note: The compound exists as a mixture of rotamers.

N-ethyl-6-fluoro-*N*-(4-formylbenzyl)nicotinamide **4b**

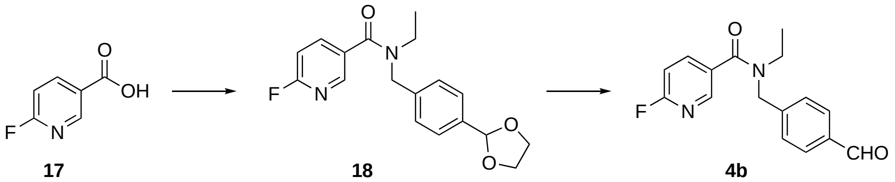

N-(4-(1,3-dioxolan-2-yl)benzyl)-N-ethyl-6-fluoronicotinamide 18

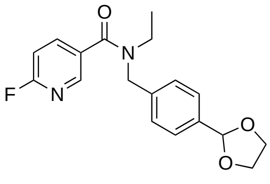

A dry 100 mL round-bottom flask was charged with 6-fluoronicotinic acid, **17**, (0.352 g, 2.5 mmol), evacuated and refilled with argon (3×). Dry toluene (10 mL) followed by oxalyl chloride (0.318 mg, 0.22 mL, 1.0 equiv), and DMF (2 drops) were injected. The reaction was stirred at room temperature for 5 minutes and at 40 °C for a further hour. Dry methylene chloride (10 mL) was injected, and the reaction cooled to -78 °C. Triethyl amine (1.4 mL, 10.0 mmol, 4.0 equiv) was injected followed by a solution of *N*-(4-(1,3-dioxolan-2-yl)benzyl)ethanamine, **A**, (0.519 g, 2.5 mmol, 1.0 equiv) in dry methylene chloride (2 mL). The reaction was allowed to reach room temperature overnight, and then quenched with water (15 mL) and K_2CO_3 (15 mL, sat. aq), and then extracted with methylene chloride (50 mL × 3). The combined methylene chloride phases were rinsed with water (30 mL), brine (30 mL) and then dried over $MgSO_4$.The organic solvents were removed under reduced pressure to yield a yellow oil. Purification *via* flash chromatography [silica gel (40 g), ethyl acetate/methylene chloride (0:100) to (20:80)] to yield *N*-(4-(1,3-dioxolan-2-yl)benzyl)-*N*-ethyl-6-fluoronicotinamide, **18**, as a colourless solid (0.8 g, 97%).

Mp = 62-63 °C. ^{19}**F NMR*** (282 MHz, DMSO-d_6): δ [ppm]= –65.1, –65.32. ^{1}**H NMR*** (700 MHz, DMSO-d_6): δ [ppm]= 1.03-1.12 (m, 3H, CH_3CH_2), 3.17-3.41 (m, 2H, CH_3CH_2),3.94-4.04 (m, 2H, Ar-CH_2), 4.51 (m, 2H, OCH_2CH_2O), 4.72 (m, 2H, OCH_2CH_2O), 5.72 (s, 1H, OC*H*O), 7.21-7.27 (m, 2H, Ar-*H*), 7.41-7.43 (m, 3H, Ar-*H*), 8.02-8.12 (m, 1H, Ar-*H*), 8.31-8.40 (m, 1H, Ar-*H*). ^{13}**C NMR*** (176 MHz, DMSO-d_6): δ [ppm]= 12.14, 13.50, 40.01, 43.24, 46.63, 51.35, 64.81, 102.67, 109.54, 109.76, 126.60, 126.79, 126.95, 127.45, 131.03, 137.03, 137.30, 137.93, 138.49, 140.53, 140.58, 145.50, 145.58, 162.9 (d, J_{FC} = 238 Hz) 167.3,

167.5. **IR** (ATR, neat): 1/λ [cm^{-1}] =2978, 2881, 1617, 1590, 1464, 1433, 1374, 1311, 1250, 1081, 979. **HRMS** (ESI) m/z [M+H]$^+$: calcd. for $C_{18}H_{20}FN_2O_3$: 331.1458, found: 331.1475.

*Note: The compound exists as a mixture of rotamers.

N-ethyl-6-fluoro-*N*-(4-formylbenzyl)nicotinamide 4b

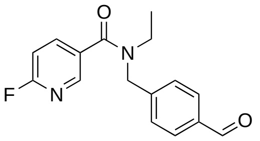

A 50 mL round-bottom flask was charged with *N*-(4-(1,3-dioxolan-2-yl)benzyl)-*N*-ethyl-6-fluoronicotinamide, **18**, (0.33 g, 1.0 mmol) and THF (20 mL). Conc. HCl was added until acidic (pH~1), and the resulting solution was stirred overnight at room temperature. The reaction was quenched with aqueous K_2CO_3 (sat.) until alkaline (pH~11), and then extracted with methylene chloride (50 mL × 3). The combined organic phases were dried over $MgSO_4$ and the organic solvents were removed under reduced pressure to yield a clear oil. Purification *via* flash chromatography [silica gel (40 g), ethyl acetate/methylene chloride (0:100) to (20:80)] to yield *N*-ethyl-6-fluoro-*N*-(4-formylbenzyl)nicotinamide, **4b**, as a clear oil (0.28 g, 98%).

^{19}F NMR (282 MHz, $CDCl_3$): δ [ppm]= –64.7. **^{1}H NMR*** (600 MHz, $CDCl_3$): δ [ppm]= 1.14-1.23 (m, 3H, C*H*$_3$CH$_2$), 3.29-3.56 (m, 2H, CH$_3$C*H*$_2$), 4.61-4.82 (m, 2H, Ar-C*H*$_2$), 6.93-7.01 (m, 1H, Ar-*H*), 7.35-7.50 (m, 2H, Ar-*H*), 7.88-7.91 (m, 3H, Ar-*H*), 8.29-8.35 (m, 1H, Ar-*H*), 10.01 (s, 1H, C*H*O). **^{13}C NMR*** (150 MHz, $CDCl_3$): δ [ppm]= δ 12.3, 14.0, 41.0, 43.8, 47.7, 52.2, 110.0 (d, *J* = 37.1 Hz), 128.6, 130.2, 130.4, 135.9, 140.28, 140.34, 144.1, 146.0, 163.9 (d, J_{FC} = 243.0 Hz), 168.6, 191.8. **IR** (ATR, neat): 1/λ [cm^{-1}] = 2974, 1695, 1633, 1607, 1577, 1476, 1418, 1291, 1212, 1166. **HRMS** (ESI) m/z [M+H]$^+$: calcd. for $C_{16}H_{16}FN_2O_2$:287.1196, found:287.1194.

*Note: The compound exists as a mixture of rotamers.

4-((ethyl((5-fluoropyridin-3-yl)methyl)amino)methyl)benzaldehyde 5

A dry 50 mL round-bottom flask was charged with *N*-(4-(1,3-dioxan-2-yl)benzyl)-*N*-ethyl-5-fluoronicotinamide, **16**, (0.344 g, 1.0 mmol), evacuated and re-filled with argon (3×). Dry THF (10 mL) was injected and the resulting solution was cooled to 0 °C. Lithium aluminium hydride (2.0 mL, 1M in THF 2.0 mmol, 2.0 equiv) was injected over 5 minutes and then stirred for one hour more at 0 °C. The reaction was carefully quenched at 0 °C with water (0.1 mL) followed by NaOH (0.1 mL, 15% aq.) and water (0.3 mL). The reaction was stirred for a further 30 minutes at room temperature, diluted with THF (30 mL) and then dried with $MgSO_4$. This was filtered over a plug of celite (3 cm × 3 cm) to remove the aluminum salts. The organic solvents were removed under pressure to yield a yellow oil that was dissolved in wet THF (10 mL) and added to a 50 mL round-bottom flask. Conc. HCl was added until acidic (pH~1), and the resulting solution was stirred overnight at room temperature. The reaction was quenched with aqueous K_2CO_3 (sat.) until alkaline (pH~11), and then extracted with methylene chloride (50 mL × 3). The combined organic phases

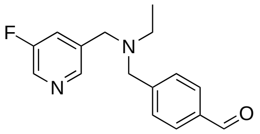

were dried over $MgSO_4$ and the organic solvents were removed under reduced pressure to yield a clear oil. Purification *via* flash chromatography [silica gel (24 g), methanol/dichloromethane (0:100) to (10:90)] to yield 4-((ethyl((5-fluoropyridin-3-yl)methyl)amino)methyl)benzaldehyde as a clear oil (0.13 g, 47%).

^{19}F NMR (282 MHz, $CDCl_3$): δ [ppm]= -127.4. **^{1}H NMR** (500 MHz, $CDCl_3$): δ [ppm]= 1.09 (t, *J* = 7.1 Hz, 3H, C*H*$_3$CH$_2$), 2.53 (q, *J* = 7.1 Hz, 2H, CH$_3$C*H*$_2$), 3.60 (s, 2H, Ar-C*H*$_2$), 3.65 (s, 2H, Ar-C*H*$_2$), 7.45 (m, 1H, Ar-*H*), 7.52 (d, *J* = 8.2 Hz, 2H, Ar-*H*), 7.83 (d, *J* = 8.2 Hz, 2H, Ar-*H*), 8.34 (m, 1H, Ar-*H*), 8.37 (s, 1H, Ar-*H*), 9.98 (s, 1H, C*H*O). **^{13}C NMR** (125 MHz, $CDCl_3$): δ [ppm]= 12.0, 47.7, 54.7, 57.9, 122.8 (d, *J* = 17.9 Hz), 129.2, 130.0, 135.6, 137.0 (d, *J* = 23.3 Hz), 137.3 (d, *J* = 3.1 Hz), 145.8 (d, *J* = 3.7 Hz), 146.9, 159.8 (d, *J* = 256.5 Hz), 192.0 (d, *J* = 7.1 Hz). **IR** (ATR, neat): 1/λ [cm^{-1}] =2968, 2816, 1695, 1604, 1576, 1427, 1373, 1264, 1207, 1163. **HRMS** (ESI) m/z $[M+H]^+$: calcd. for $C_{16}H_{18}FN_2FO_2$: 273.1403, found: 273.1400.

N-ethyl-5-fluoro-*N*-(4-formylbenzyl)pyridine-3-sulfonamide **6**

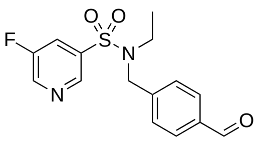

A dry 50 mL round-bottom flask was charged with 5-fluoropyridine-3-sulfonyl chloride, **19**, (0.25 g, 1.27 mmol), evacuated and refilled with argon (3×). Dry methylene chloride (10 mL) was injected and the resulting solution was cooled to 0°C. Triethylamine (0.53 mL, 0.386 g 3.8 mmol, 3.0 equiv) was injected followed by a solution of *N*-(4-(1,3-dioxolan-2-yl)benzyl)ethanamine, **A**, (0.32 g, 1.53 mmol, 1.2 equiv) in dry methylene chloride (2 mL). The reaction was allowed to reach room temperature overnight, and then quenched with water (15 mL) and K_2CO_3 (15 mL, sat. aq), and then extracted with methylene chloride (50 mL × 3). The combined organic phases were rinsed with water (30 mL), brine (30 mL) and then dried over $MgSO_4$.The organic solvents were removed under reduced pressure to yield a yellow oil that was dissolved in wet THF (10 mL) and added to a 50 mL round-bottom flask. Conc. HCl was added until acidic (pH~1), and the resulting solution was stirred overnight at room temperature. The reaction was quenched with aqueous K_2CO_3 (sat.) until alkaline (pH~11), and then extracted with methylene chloride (50 mL × 3). The combined organic phases were dried over $MgSO_4$ and the organic solvents were removed under reduced pressure to yield a clear oil. Purification *via* flash chromatography [silica gel (24 g), ethyl acetate/ petroleum ether (20:80) to (40:60)] to yield *N*-ethyl-5-fluoro-*N*-(4-formylbenzyl)pyridine-3-sulfonamide as a colourless solid (0.28 g, 68%).

Mp = 85 °C. **^{19}F NMR** (282 MHz, $CDCl_3$): δ [ppm]= –122.8. **^{1}H NMR** (300 MHz, $CDCl_3$): δ [ppm]= 0.99 (t, *J* = 7.2 Hz, 3H, C*H*$_3$CH$_2$), 3.29 (q, *J* = 7.2 Hz, 2H, CH$_3$C*H*$_2$), 4.47 (s, 2H, Ar-C*H*$_2$), 7.49 (d, *J* = 8.0 Hz, 2H, Ar-*H*), 7.81 (m, 1H, Ar-*H*), 7.86 (d, *J* = 8.2 Hz, 2H, Ar-*H*), 8.68 (d, *J* = 2.6 Hz, 1H, Ar-*H*), 8.87 (s, 1H, Ar-*H*), 10.01 (s, 1H, C*H*O). **^{13}C NMR** (75 MHz, $CDCl_{3:}$) δ [ppm]= 13.6, 43.4, 51.1, 121.7 (d, *J* = 20.4 Hz), 128.6, 130.3, 136.2, 137.9, 142.1(d, *J* = 23.6 Hz), 142.8, 143.6 (d, *J* = 4.6 Hz), 158.8 (d, *J* = 263.6 Hz), 191.7. **IR**

(ATR, neat): 1/λ [cm^{-1}] = 3052, 1687, 1605, 1576, 1420, 1343, 1301, 1239, 1210, 1151, 1011, 896. **HRMS** (ESI) m/z $[M+H]^+$: calcd. for $C_{15}H_{16}FN_2FO_3S$: 323.0860, found: 323.0861.

N-(4-(1,3-dioxolan-2-yl)benzyl)-6-chloro-*N*-ethylnicotinamide **7**

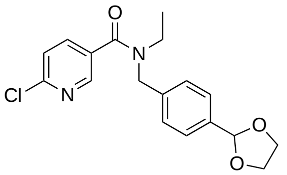

A dry 100 mL round-bottom flask was charged with 6-chloronicotinic acid, **20**, (0.393 g, 2.5 mmol), evacuated and refilled with argon (3×). Oxalyl chloride (0.317 g, 0.214 mL, 1.0 equiv), and DMF (2 drops) were injected. The reaction was stirred at 40 °C for 1 h. Dry methylene chloride (20 mL) was injected, and the reaction cooled to -78°C. Triethylamine (1.4 mL, 10.0 mmol, 4.0 equiv) was injected followed by a solution of *N*-(4-(1,3-dioxolan-2-yl)benzyl)ethanamine, **A**,(0.518 g, 2.5 mmol, 1.0 equiv) in dry methylene chloride (2 mL). The reaction was allowed to reach room temperature overnight, and then quenched with water (15 mL) and K_2CO_3 (15 mL, sat. aq), and then extracted with methylene chloride (50 mL × 3). The combined organic phases were rinsed with water (30 mL), brine (30 mL) and then dried over $MgSO_4$. The organic solvents were removed under reduce pressure to yield a yellow oil. Purification *via* flash chromatography [silica gel (40 g), ethyl acetate/methylene chloride (0.2% Et_3N) (15:85) to (25:75)] to yield *N*-(4-(1,3-dioxolan-2-yl)benzyl)-6-chloro-*N*-ethylnicotinamide, **7**, as a colourless solid (0.65 g, 75%).

Mp = 57-58 °C. **^{1}H NMR*** (600 MHz, $CDCl_3$): δ [ppm]= 1.09-1.22 (m, 3H, CH_3CH_2), 3.18-3.54 (m, 2H, CH_3CH_2), 4.04 (m, 2H, OCH_2CH_2O), 4.13 (m, 2H, OCH_2CH_2O), 4.51-5.73(m, 2H, Ar-CH_2), 5.80 (s, 1H, OC*H*O), 7.18 (brs, 1H, Ar-*H*), 7.28-7.41 (m, 2H, Ar-*H*), 7.47-7.49 (m, 2H, Ar-*H*), 7.68-7.73 (m, 1H, Ar-*H*), 8.46-8.47 (m, 1H, Ar-*H*). **^{13}C NMR*** (150 MHz, $CDCl_3$): δ [ppm]= 12.3, 13.8, 40.6, 43.0, 47.1, 51.9, 65.5, 103.3, 103.5, 124.3, 124.4, 126.5, 127.1, 127.4, 128.4, 131.1, 131.4, 137.3, 137.5, 137.8, 137.9, 147.4, 147.8, 152.5, 152.6, 168.3. **IR** (ATR, neat): 1/λ [cm^{-1}] = 1630, 1423, 1106, 1020, 940. **HRMS** (ESI) m/z $[M+H]^+$: calcd. for $C_{18}H_{20}Cl\ N_2O_3$:347.1162, found: 347.1158.

*Note: The compound exists as a mixture of rotamers.

4-((*N*-ethyl-6-fluoronicotinamido)methyl)benzoic acid **8**

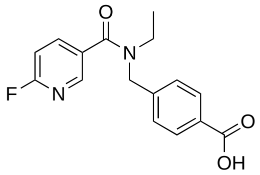

A 10 mL round-bottom flask was charged with *N*-ethyl-6-fluoro-*N*-(4-formylbenzyl)nicotinamide, **4b**, (0.082 g, 0.286 mmol), *tert*-butanol (1.5 mL) and water (1.5 mL) and the resulting solution was cooled to 0 °C in an ice bath. Potassium permanganate (0.056 g, 0.357 mmol, 1.25 equiv) in water (1.5 mL) followed by NaOH (1.5 mL, 10%, aq) were injected. The cooling bath was removed and the reaction was stirred at room temperature overnight. The reaction was quenched with sodium thiosulfate (20% aq) until the purple colour disappeared, and the pH was adjusted to 2-3 by the addition of HCl (appx. 2 mL, 2N, aq), and then extracted with ethyl acetate (25 mL × 3). The combined organic phases were dried

over $MgSO_4$. The organic solvents were removed under reduced pressure to yield a sticky colourless residue. Purification *via* flash chromatography [silica gel (8 g), methanol/methylene chloride (0.2% Et_3N) (0:100) to (20:80)] to yield 4-((*N*-ethyl-6-fluoronicotinamido)methyl)benzoic acid, **8**, as a colourless solid (0.038 g, 44%).

Mp = 141 °C ^{19}**F NMR** (282 MHz, DMSO-d_6): δ [ppm]= –66.9. ^{1}**H NMR*** (600 MHz, DMSO-d_6): δ [ppm]= 1.03-1.13 (m, 3H, CH_3CH_2), 3.19-3.42 (m, 2H, CH_3CH_2), 4.56-4.77 (m, 2H, Ar-CH_2), 7.21-7.49 (m, 3H, Ar-*H*), 7.93 (brs, 2H, Ar-*H*), 8.0-8.15 (m, 1H, Ar-*H*), 8.27-8.41 (m, 1H, Ar-*H*), 12.94 (s, 1H, COO*H*). ^{13}**C NMR*** (150 MHz, DMSO-d_6): δ [ppm]= 12.2, 13.6, 40.2, 43.6, 46.8, 51.4, 109.6, 109.8, 126.9, 127.5, 129.5, 130.95, 130.98, 140.6, 142.3, 142.8, 145.5, 145.6, 162.9 (d, J_{FC} = 237.4 Hz), 167.15, 167.68. **IR** (ATR, neat): 1/λ [cm^{-1}] = 2968, 2932, 1684, 1627, 1589, 1422, 1371, 1284, 1239, 1093, 1019, 929. **HRMS** (ESI) m/z $[M+H]^+$: calcd. for $C_{16}H_{16}FN_2O_3$: 303.1139, found: 303.1138.

*Note: The compound exists as a mixture of rotamers.

N-ethyl-6-fluoro-*N*-(4-(hydroxymethyl)benzyl)nicotinamide 9

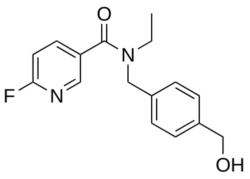

A 25 mL round-bottom flask was charged with *N*-ethyl-6-fluoro-*N*-(4-formylbenzyl)nicotinamide, **4b**, (0.1 g, 0.349 mmol),and MeOH (6 mL).Sodium borohydride (0.019 g, 0.49 mmol, 1.4 equiv) was added and the reaction was stirred at room temperature for 30 minutes. The organic solvents were removed under reduced pressure to yield a white residue that was treated with water (5 mL) and then extracted with methylene chloride (25 mL × 3). The combined organic phases were dried over $MgSO_4$. The solvents were removed under reduced pressure to yield a yellow oil. Purification *via* flash chromatography [silica gel (8 g), ethyl acetate/methylene chloride (20:80) to (80:20)] to yield *N*-ethyl-6-fluoro-*N*-(4-(hydroxymethyl)benzyl)nicotinamide, **9**, as a clear oil (0.099 g, >95%).

^{19}**F NMR** (282 MHz, $CDCl_3$): δ [ppm]= –65.2. ^{1}**H NMR*** (600 MHz, $CDCl_3$): δ [ppm]= 1.13-1.26 (m, 3H, CH_3CH_2), 3.23-3.55 (m, 2H, CH_3CH_2), 4.52 (m, 1H, Ar-CH_2), 4.7 (s, 2H, Ar-CH_2OH), 4.76(m, 1H, Ar-CH_2) 6.93-7.0 (m, 1H, Ar-*H*), 7.16 (brs, 1H, Ar-*H*), 7.36-7.38 (m, 3H, Ar-*H*), 7.88 (brs, 1H, Ar-*H*), 8.32 (brs, 1H, Ar-*H*). ^{13}**C NMR*** (150 MHz, $CDCl_3$): δ [ppm]= 12.3, 13.9, 40.7, 43.2, 47.3, 52.1, 65.1, 109.7, 109.9, 126.7, 127.63, 128.5, 130.5, 135.7, 136.4, 140.2, 140.3, 146.1, 163.9 (d, *J* = 242.6 Hz), 168.42. **IR** (ATR, neat): 1/λ [cm^{-1}] = 3391, 2975, 2932, 2872, 1616, 1591, 1417, 1368, 1289, 1249, 1018. **HRMS** (ESI) m/z $[M+H]^+$: calcd. for $C_{16}H_{18}FN_2FO_2$:289.1347, found: 289.1344.

*Note: The compound exists as a mixture of rotamers.

6-chloro-*N*-ethyl-*N*-(4-formylbenzyl)nicotinamide **10**

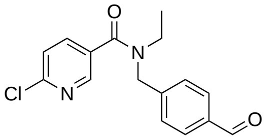

A 50 mL round-bottom flask was charged with *N*-(4-(1,3-dioxolan-2-yl)benzyl)-6-chloro-*N*-ethylnicotinamide, **7**, (0.8 g, 2.23 mmol), THF (20 mL) and water (2 mL). To this solution, HCl (1 mL, 11M, 11.0 mmol, 5 equiv) was added and allowed to stir over night at room temperature. The reaction was quenched with water (10 mL) and K_2CO_3 (appx. 15 mL, sat. aq.), and then extracted with methylene chloride (50 mL × 3). The combined organic phases were rinsed with water (30 mL), brine (30 mL) and then dried over $MgSO_4$. The organic solvents were removed under reduced pressure to yield a clear oil. Purification *via* flash chromatography [silica gel (40 g), ethylacetate/methylene chloride (0.2% Et_3N) (15:85) to (25:75)] to yield 6-chloro-*N*-ethyl-*N*-(4-formylbenzyl)nicotinamide, **10**, as a colourless solid (0.68 g, 97%).

Mp = 52-53 °C. **^{1}H NMR*** (600 MHz, $CDCl_3$): δ [ppm]= 1.14-1.25 (m, 3H, C*H*$_3$CH$_2$), 3.27-3.57 (m, 2H, CH$_3$C*H*$_2$), 4.59-4.82 (m, 2H, Ar-C*H*$_2$), 7.34-7.50 (m, 3H, Ar-*H*), 7.69-7.76 (m, 1H, Ar-*H*), 7.88-7.90 (m, 2H, Ar-*H*), 8.43-8.50 (m, 1H, Ar-*H*), 10.01 (s, 1H, C*H*O). **^{13}C NMR*** (150 MHz, $CDCl_3$): δ [ppm]= δ 12.3, 14.0, 41.0, 43.8, 47.7, 52.1, 124.5, 127.1, 128.6, 130.4, 131.0, 135.9, 137.4, 143.9, 147.5, 152.8, 168.5, 191.8. **IR** (ATR, neat): 1/λ [cm^{-1}] = 1690, 1630, 1578, 1415, 1273, 1210 1104, 1075, 1015, 985. **HRMS** (ESI) m/z [M+H]$^+$: calcd. for $C_{16}H_{16}ClN_2O_2$:303.0894, found: 303.0890.

*Note: The compound exists as a mixture of rotamers.

RADIOCHEMISTRY

GENERAL CONSIDERATIONS

All labelling reactions were performed manually using [^{18}F]fluoride in [^{18}O]H_2O (50-200 MBq) for screening studies. For preclinical tracer synthesis of [^{18}F]**4b** the reactions were either performed manually or using an HotBox III module (Scintomics, Fürstenfeldbruck, De) using 1-2 GBq [^{18}F]fluoride. Radio-HPLC were performed with an Agilent 1200 HPLC system equipped with a 1200 Series Diode Array Detector and a GABI Star NaI(Tl) scintillation detector (energy window 400-700 keV). The system was used for purification as well as characterisation of radiotracers. Columns and conditions used for purification and quality control (QC) are indicated in the protocol or next to the corresponding chromatogram.

Radiochemical yields were calculated as follows:

- Analytical radiochemical yields (ARCY) were determined using radio-HPLC chromatograms of the quenched crude labelling mixture and refer to the area under the curve (AUC) of the radioactive peak of interest divided by the summed AUC of all other radioactive peaks ([^{18}F]fluoride and potential side-products).
- Isolated radiochemical yields (RCY) refer to the activity of the pure tracer isolated after HPLC divided by the initial activity of [^{18}F]fluoride in [^{18}O]H_2O used for the labelling. Unless otherwise stated, isolated RCY are given decay corrected.

CARTRIDGES CONDITIONING AND CRYPTAND MIXTURE PREPARATION

Solid Phase Extraction (SPE) cartridges used in the radiolabelling experiments were conditioned prior to use as follows:

- Sep-Pak® Accell Plus QMA Plus Light Cartridge (130 mg, Waters Cat. no. WAT023525) were flushed successively with aqueous sodium hydroxide (1M, 5 mL), HPLC water (10 mL), aqueous potassium carbonate (1M, 1 mL), HPLC water (10 mL) and air (10 mL).
- Sep-Pak® Alumina N Plus Light Cartridge (280 mg, Waters Cat. no. WAT023561) were flushed with HPLC water (1 mL) and air-dried (5 mL).
- Sep-Pak® tC18 Plus Light Cartridge (145 mg, Waters Cat. no. WAT036805) was flushed with methanol (5 mL), HPLC water (10 mL) and air-dried (10 mL).
- Unless otherwise stated, all radiolabelling experiments were carried-out with a cryptand mixture composed of Kryptofix 222 and potassium carbonate in acetonitrile/water. Typically, a solution of potassium carbonate (2.6 mg) in HPLC water (0.2 mL) was added to a

solution of Kyptofix 222 (14.2 mg) in acetonitrile (1.1 mL). 0.6 mL of the resulting solution was used for each labelling experiment.

^{18}F-FLUORINATION OF 4b

OPTIMISATION

[^{18}F]Fluoride (50–200 MBq per reaction, < 100 µL) in ^{18}O-water was introduced in a V-shaped vial, followed by addition of a mixture of Kryptofix 222 and K_2CO_3. After removing the solvent by heating at 90 °C under a stream of nitrogen, dry acetonitrile (0.5 mL) was added, and the distillation was continued at 90 °C. This procedure was repeated once and the reaction vial was subsequently capped. The precursor, **7** (0.5-5 mg) dissolved in the dry solvent (0.6 mL) was added and the mixture was stirred for the specified duration at the specified temperature. The acetal cleavage-step was mediated with aqueous HCl (1M, 1.0 mL) at 110 °C for 5 minutes (Table S1). After cooling, the reaction was quenched with water (1.0 mL) and the mixture was analysed by radio-HPLC on an Eclipse® C18 4.6 × 100 mm, 5 µm HPLC column using water and methanol (gradient elution with a flow rate of 1.8 mL/min starting with 25% methanol content, then increased to 50% in 7 min; Rt ≈ 6.2 min).

Table S1: Optimisation of radiolabeling of **7**

Solvent	7 [mg]	Temp [°C]	Time [min]		Analytical RCY (%)	
			Labelling	Acetal Cleavage	[^{18}F]4bb	[^{18}F]4b
DMSO	5	110	15	–	40	_
DMSO	5*	110	15	–	_	3
DMSO	5	150	15	–	70	NA
DMSO	5	150	5	5	–	33
DMF	5	150	15	5	–	28
DMSO	5	150	25	5	–	51

Note: * *N*-ethyl-*N*-(4-formylbenzyl)-6-nitronicotinamide **10** was used instead of **7**.

MANUAL RADIOLABELLING OF [^{18}F]4b

[^{18}F]Fluoride (1.1–1.3 GBq per reaction) in ^{18}O-water was trapped on a Sep-Pak® QMA cartridge, released with a solution (0.6 mL) of Kryptofix 222 and potassium carbonate solution. After removing the solvent by heating at 90 °C under a stream of nitrogen, dry acetonitrile (0.5 mL) was added, and the distillation was continued at 90 °C. This procedure was repeated once and the reaction vial was subsequently capped. The chloro-precursor **7** (0.5-5 mg) dissolved in anhydrous DMSO (0.6 ± 0.05 mL) was added and the mixture was stirred for 25 min at 150 °C. After cooling the reaction to 110 °C, aqueous HCl (0.5 mL, 1M) was injected and the reaction stirred for 5 minutes. The reaction was cooled to room temperature following

which it was quenched by the addition of aqueous NH_4OAc (1.0 mL, 0.02M) and purified *via* semi preparative HPLC using a ZORBAX ® StableBond 300 C18, 9.4 x 250 mm, 5 µm HPLC column at room temperature and with a flow rate of 3.5 mL/min. The mobile phase consisted of aqueous NH_4OAc (0.02M) and methanol. Isocratic elution with 36.5% methanol content allowed for isolation of the radioactive product (Rt ≈ 18 min). The isolated fraction was diluted with water (20 mL) containing Na-ascorbate (5 mg) and passed through a Sep-Pak® Alumina N Plus Light Cartridge followed by a Sep-Pak® tC18 Plus Light Cartridge. The Sep-Pak® tC18 Plus Light Cartridge was rinsed with water (5 mL) and then pure [^{18}F]**4b** was eluted from the Sep-Pak tC18 cartridge with ethanol (0.5 mL). The ethanol was removed under a flow of N_2 for 3 minutes at 60 °C, and the residue was reformulated in sterile saline (1 mL, 0.9% NaCl) which was passed through a sterile filter into a sealed evacuated sterile vial. The decay corrected isolated RCY was 35 ± 1% (*n* = 3) at the end of HPLC purification. The radiochemical purity of [^{18}F]**4b** was > 99% and the molar activity of the tracer was 2.8-4.4 GBq/µmol when starting from appx. 1 GBq of [^{18}F]fluoride. The identity of the radiochemical product was confirmed by co-elution with the non-radioactive analogue. The total synthesis time, from [^{18}F]fluoride in water to formulation on sterile saline was 2h15 min.

AUTOMATED RADIOLABELLING OF [^{18}F]4b

HotBox III module (Scintomics, Fürstenfeldbruck, De) was used to perform this synthesis.

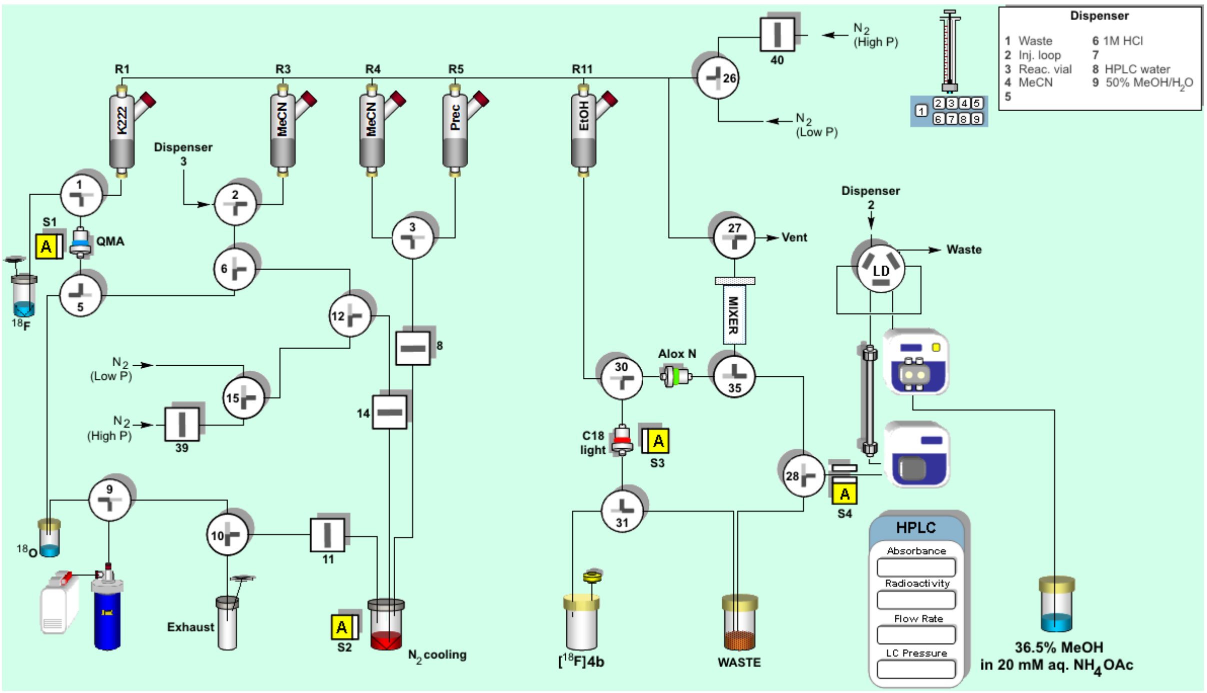

Panel S1: The interface of the Scintomics HotBox III radiochemical synthesiser.

Reaction sequence of $[^{18}F]$**4b** synthesis

1. $[^{18}F]$Fluoride (0.8–2.2 GBq) in ^{18}O-water was trapped trapping on Sep-Pak® QMA cartridge
2. $[^{18}F]$Fluoride elution with (0.6 mL) K222/K_2CO_3 solution
3. Azeotropic drying at 90 °C for 3 min (3X)
4. Addition of chloro-precursor **7** in dry DMSO (0.5 mL) to the reaction vial
5. Reaction at 150 ° C for 25 min
6. Cooling the reaction vial to 110 °C
7. Addition of HCl (1M, 0.5 mL)
8. Reacting at 110 °C for 5 min
9. Cooling the reaction vial to 25 °C
10. Quench: Addition of ammonium acetate buffer (4 mL)
11. Purification: semiprep-HPLC
12. Collection of the $[^{18}F]$**4b** peak
13. Dilution of the collected fraction with sterile water (35 mL)
14. Filtration through an alumina (Alox) cartridge
15. Trapping of the product on a C18 cartridge
16. Elution with ethanol (0.5 mL)

Reformulation and sterile filtration

1. Evaporation of EtOH at 60 °C for 3 min.
2. Formulation with sterile saline (1 mL)
3. Sterile filtration

Note: The reformulation and sterile filtration were performed manually

HPCL: ZORBAX ® StableBond 300 C18, 9.4 x 250 mm, 5 µm HPLC column at room temperature and with a flow rate of 3.5 mL/min. The mobile phase consisted of aqueous NH_4OAc (0.02M) and methanol. Isocratic elution with 36.5% methanol content allowed for isolation of the radioactive product (Chromatogram S1, Rt ≈ 18 min).

The decay corrected isolated RCY was 43 ± 1% (n = 3) at the end of HPLC purification and cartridge formulation. The identity of the radiochemical product was confirmed by co-elution with the non-radioactive analogue (Chromatogram S1 and S2). The radiochemical purity of $[^{18}F]$**4b** was > 99% and the molar activity of the tracer was 0.6-7.2 GBq/µmol.

MOLAR ACTIVITY

The Molar Activity of [^{18}F]**4b** was assessed by radio-HPLC using an analytical Eclipse® C18 (4.6 × 100) mm, 5 µm HPLC column, eluting with water and methanol (gradient elution with a flow rate of 1.8 mL/min starting with 25% methanol content, then increased to 50% in 7 min; Rt ≈ 6.2 min), monitoring with UV (254 nm) and radioactivity detector.

Calibration Curve

Stock **4b** solution: **4b** (1.3 mg, 4.54 µmol) was dissolved in HPLC grade methanol, and made up-to the mark in a standard 5mL flask. [c = 0.26 mg/mL]. Serial dilution: stock **4b** solution (0.5 mL) was diluted with water (0.5 mL) to generate solution **S1**. Similarly, **S1** (0.5 mL) was diluted with water (0.5 mL) to generate solution **S2**. This was repeated until **S11**. 20 µL of these solutions (S1-S11) were analysed on an Eclipse® C18 (4.6 × 100) mm, 5 µm HPLC column using water and methanol (gradient elution with a flow rate of 1.8 mL/min starting with 25% methanol content, then increased to 50% in 7 min; Rt ≈ 6.2 min). Monitoring with UV (254 nm).

Table S2: Molar Activity calculations for [^{18}F]**4b**

Solution	C [mg/mL]	m inj [µg]	Moles inj [µmol]	Area[mAU*min]
S1	0.13	2.6	0.009081066	3882.5
S2	0.065	1.3	0.004540533	1802.2
S3	0.0325	0.65	0.002270266	884.1
S4	0.01625	0.325	0.001135133	451
S5	0.008125	0.1625	0.000567567	208
S6	0.0040625	0.08125	0.000283783	104
S7	0.00203125	0.040625	0.000141892	52.5
S8	0.001015625	0.0203125	7.09458E-05	26.2
S9	0.000507813	0.01015625	3.54729E-05	13.4
S10	0.000253906	0.005078125	1.77365E-05	8.2
S11	0.000126953	0.002539063	8.86823E-06	5.1

ENZYME KINETICS

ALDH substrate kinetics were measured using an ALDH activity colorimetric assay kit (Biovision) according to the manufacturer's instructions. The assay protocol was modified to assess compounds **1**-**6** at 20 µM to 20 mM final concentrations with 1 µg/well human recombinant ALDH1A1 (bio-techne), ALDH2 (abcam), or ALDH3A1 (ATGen Co., Ltd). For ALDH3A1, 4 mM NADP+ was supplemented to all wells. The assay was initiated by the addition of enzyme after all reagents were pre-warmed to 37°C. Plates were read in kinetic mode on a Multiskan FC plate reader (Thermo Scientific) at 450 nm over 40 min at 37°C. The final reaction mixture contained 5% *v/v* DMSO in all wells. The initial reaction rate was expressed in units per microgram enzyme, where 1 unit of enzyme activity is defined as the amount of enzyme that reduces 1 nmol $NAD(P)^+$ in 1 min at 37°C, pH 8. Michaelis-Menten rate constants were fitted using GraphPad Prism (v.6.0h).

The concentration dependence of **2-6** on the rate of reaction was fit to a modified Michaelis-Menten equation accounting for substrate inhibition (Equation 1) to give an apparent K_M and V_{max} for the three isozymes (Table 1), where *V* is the initial reaction velocity, [S] is the substrate concentration, and K_i is the dissociation constant for substrate binding.

$$V = \frac{V_{max}[S]}{K_M + [S]\left(\frac{1+[S]}{K_i}\right)} \quad \text{(Eq.1)}$$

For the assessment of inhibitory kinetics for DEAB, 1 nM to 1 mM of compound was assayed in the presence of 500 µM propanal using the modified ALDH activity colorimetric assay described above. The final reaction mixture contained 5% *v/v* DMSO in all wells. The Ki of inhibition was calculated using the following equation, where [I] is the concentration of inhibitor and K_m is the Michaelis-Menten constant for propanal:

$$K_i = \frac{IC_{50}}{1 + \frac{[I]}{K_m}} \quad \text{(Eq.2)}$$

DOCKING STUDIES

Sequences for all 19 isozymes of ALDH1A1 were collected form Uniprot[6] and aligned using MUSCLE.[7] Protein structures were compared in UCSF Chimera.[8] Fpocket2.0[9] was utilized to analyse features of the pocket environment. The diameter of the substrate pocket entrance tunnel was investigated using HOLE2.[10] The substrates of interest (compounds **2**, **3a**, **3b**, **4a**, **4b**, **5**, **6**, 9-*cis*-retinal, 13-*cis*-retinal and all-*trans*-retinal) were docked into the receptor structure PDBID: 4WB9 corresponding to ALDH1A1 in the cofactor bound state. PDBID: 4L2O corresponding to inhibitor bound ALDH3A1, and PDBID: 1O01 corresponding to crotonaldehyde and cofactor bound ALDH1A1 were superposed on 4WB9 using the chimera matchmaker command (6). All residues of 4WB9 (ALDH1A1) within a 10 Å radius sphere, based on the centroid of the inhibitor in the superposed structure 4L2O (PDB ligand ID: 1DD), were included in the docking experiments. Visually this search area encompassed the immediate vicinity of the substrate tunnel. The search efficiency was automatically optimized to maximum (200%), and the ligands allowed full flexibility, 200 diverse solutions were generated with default diversity parameters. Ten amino acid side chains were selected to have full flexibility (N121, N170, F171, M175, W178, E269, Y297, C302, C303, F466). Priority was given to residues involved in the catalytic activity of the enzyme, followed by ensuring an even distribution of flexible residues around the search area to avoid bias. A scaffold match constraint with a weight of 5 was used to focus solutions to be bound with a correct orientation of the aldehyde portion. The scaffold used was taken from the aldehyde and α-carbon positions of crotonaldehyde in the superposed structure 1O01. Docking solutions were scored with two built-in scoring functions (ChemPLP and ChemScore). Solutions were analysed with an in-house written python script which selected the highest ranked solution for each compound, whereby the aldehyde was found to have a 'good' orientation with respect to the receptor. A 'good' orientation was defined as the aldehyde carbon being within 3.8 Å of the C302-γS, the aldehyde oxygen being within 5.5 Å of the N121-γN and the aldehyde O being further than the aldehyde C from C302-γS. Analysis of protein data bank (PDB) structures for each of the three isozymes of interest showed that there was little change in structure for states e.g. inhibitor or cofactor binding. RSMD between PDBID: 1O01, crotonaldehyde and NAD bound ALDH2 and PDBID: 3N80, apo ALDH2 was found to be 0.145 Å across all Cα positions. RMSD between PDBID: 3SZA apo ALDH3A1 vs. 4L2O inhibitor and cofactor bound ALDH3A1 was found to be 0.260 Å across all Cα positions. And the RMSD between 4WB9 (cofactor bound ALDH1A1) and 5L2O (inhibitor bound ALDH1A1) was found to be 0.298 Å. The structures chosen for further analysis were 4WB9, 1O01 and 4L2O for ALDH1A1, ALDH2 and ALDH3A1 respectively. This was because each of these structures contained the cofactor and were thought to be closest to the state that the enzyme would be in when a substrate was bound.

ALDH1A1 was found to share 68% sequence identity with ALDH2 and 27% sequence identity with ALDH3A1 while ALDH3A1 and ALDH2 shared 26% sequence identity, suggesting that ALDH1A1 and ALDH2 are much more similar to each other than they are to ALDH3A1. Analysis of the active site features of the chosen structures with Fpocket2.0 (4) revealed that the ALDH1A1 substrate pocket is slightly higher in hydrophobic density than that of ALDH2 or ALDH3A1 and that the ALDH3A1 substrate pocket has a reversal in polarity compared with ALDH1A1 and ALDH2, from overall positively charged pocket to a negatively charged pocket.

ALDH1A1 was also found to have the widest diameter entrance tunnel for the substrate binding pocket, similarly the tunnel remained wider than that of ALDH2 or ALDH3A1 until very close to the active cysteine residue (Fig. S6). The observation that ALDH1A1 has the largest access tunnel to the active site residue (Fig. S6) might suggest that bulkier and more rigid substrates would be preferentially turned over by ALDH1A1 vs. ALDH2 or ALDH3A1. This may also help to explain why reducing the amide in compound **4a** or **4b** to a secondary amine in compound **5**, maintains enzymatic efficiency at ALDH1A1 to a large extent but significantly increases efficiency at ALDH2 and ALDH3A1. Given the higher degree of flexibility for compound **5**, it may be more able to squeeze down a narrow entrance tunnel to the substrate binding site, while the more rigid and bent conformation of compounds **4a** and **4b**, preclude their access. The wider access tunnel to ALDH1A1 allows it to accommodate bulkier and less flexible ligands. The addition of the pyridyl ring allows the formation of a π-stacking interaction with Y296 in ALDH1A1 leading to higher binding affinities for substrates containing the pyridyl substituent. These two factors lead to compound **4a** and **4b** showing the greatest level of specificity towards ALDH1A1 over ALDH2 and ALDH3A1.

CELL ASSAYS

CELL CULTURE CONDITIONS

Human colorectal cancer HCT116 KRAS mut cells were kindly donated by Lewis Cantley (Weill Cornell Medical College). Cells were grown in DMEM media (ThermoFisher Scientific) supplemented with 10% foetal bovine serum and 100 U.mL^{-1} penicillin, 100 µg.mL^{-1} streptomycin (Sigma Alrich Ltd), maintained under standard culture conditions (37 °C, 5% CO_2, humidified atmosphere).

RADIOTRACER CELL UPTAKE EXPERIMENTS

HCT116 KRAS mutant cells were seeded (5 x 10^5 cells per well) into 6-well plates 24 h prior to uptake. 0.37 MBq of [^{18}F]**4b** was added to each well (1 mL). For treatment with DEAB, a final concentration of 30 µM was administered 15 min prior to radiotracer uptake and remained in the media throughout the uptake time course. At 20, 40 and 60 min, plates were placed on ice, washed three times with ice-cold phosphate-buffered saline (PBS) to remove exogenous radioactivity and lysed in RIPA buffer (500 µL; Fisher Scientific Ltd). Decay-corrected radioactivity in lysates was determined on a gamma counter (300 µL of lysate; 2480 WIZARD2 automated gamma counter, PerkinElmer), with the remaining cell lysate centrifuged (21,130 *g* for 10 min, 4°C) and the supernatant used to determine protein concentration following radioactive decay using a Pierce BCA assay, according to the manufacturer's instructions. To quantify radiotracer uptake in cells, three standard solutions of the radioactivity-containing medium were counted on the gamma counter. Data were expressed as a percentage of total radioactivity administered to cells per mg of protein.

RADIO-HPLC ANALYSIS

2.9 x 10^6 cells were seeded in 10 cm plates 24 h prior to radiotracer addition. [^{18}F]**4b** was added at a concentration of 0.37 MBq/mL (5 mL per plate). DEAB treatment was carried out as above. At 20, 40 and 60 min, plates were placed on ice and 30 µL of media sample added to a solution containing 470 µL methanol and 500 µL ddH_2O. Plates were washed three times with ice-cold PBS to remove exogenous radioactivity and cells harvested in a 1:1 solution of methanol and ddH_2O. On ice, cells were sonicated using three sets of five, 0.5 s pulses (Hielscher UP50H ultrasonic processor). The cell lysate and cell media samples were centrifuged at 21,130 *g* for 10 min, 4°C to remove cell debris. For protein extraction, an equal volume of TFA (1% in EtOH) was added to the supernatant, the solution immediately vortexed and centrifuged at 21,130 *g* for 10 min, at 4°C. The protein-free supernatant was collected and kept on ice prior to analysis. The protein free supernatant appx. (0.5 mL) was treated with aqueous NH_4OAc (4 mL, 0.1M), and to it was added 12.3 µmol of each of cold-standard (**4b**, **8**, **9**, and 6-fluoronicotinic acid) prior to analysis *via* radio-HPLC.

PET SCANS

All animal experiments were performed in accordance with the United Kingdom Home Office Animal (scientific procedures) Act 198. Female balb/c mice (aged 6-9 weeks, Charles River Laboratories) were anaesthetized with isoflurane (2.5% in oxygen) and maintained at 37 °C using an air-heated scanning bed. A tail vein cannula was inserted and a 3.7 MBq bolus of [^{18}F]**4b** was administered in approximately 100 µL of PBS. Immediately following injection, 120 min PET acquisition was performed on a Mediso nanoScan PET/CT system. CT images were acquired for anatomical visualization and attenuation correction (480 projections; helical acquisition; 50kVp; 300 ms exposure time). Reconstructed images (Tera-Tomo 3D reconstruction algorithm; 4 iterations; 6 subsets; 400-600 keV; 0.4 mm voxel size) were analysed using VivoQuant software (v. 2.5, Invicro Ltd.). Regions of interest (ROIs) were drawn manually on the CT images and the radioactivity concentration in each ROI expressed as a percentage of the injected dose per mL of tissue volume (%ID/mL).

BLOOD METABOLITE ANALYSIS

Female balb/c mice (aged 6-9 weeks, Charles River Laboratories) were anaesthetized with isoflurane (2.5% in oxygen) and maintained at 37 °C on a heated mat. A tail vein cannula was inserted and a 3.7 MBq bolus of [^{18}F]**4b** administered in approximately 100 µL of PBS. At 2, 5, 20, 30 and 60 min post [^{18}F]**4b** injection, approximately 1 mL of blood was collected into heparinised syringes by cardiac puncture and centrifuged to separate the plasma (21,130 g for 10 min, 4°C). An equal volume of TFA (1%, EtOH) was added to the plasma for protein precipitation, with samples immediately vortexed and centrifuged (21,130 g for 10 min, 4°C). The protein free supernatant appx. (0.5 mL) was treated with aqueous NH_4OAc (4 mL, 0.1M), and to it was added 12.3 µmol of each of cold-standard (**8** and 6-fluoronicotinic acid) prior to analysis *via* radio-HPLC.

SUPPLEMENTAL FIGURES

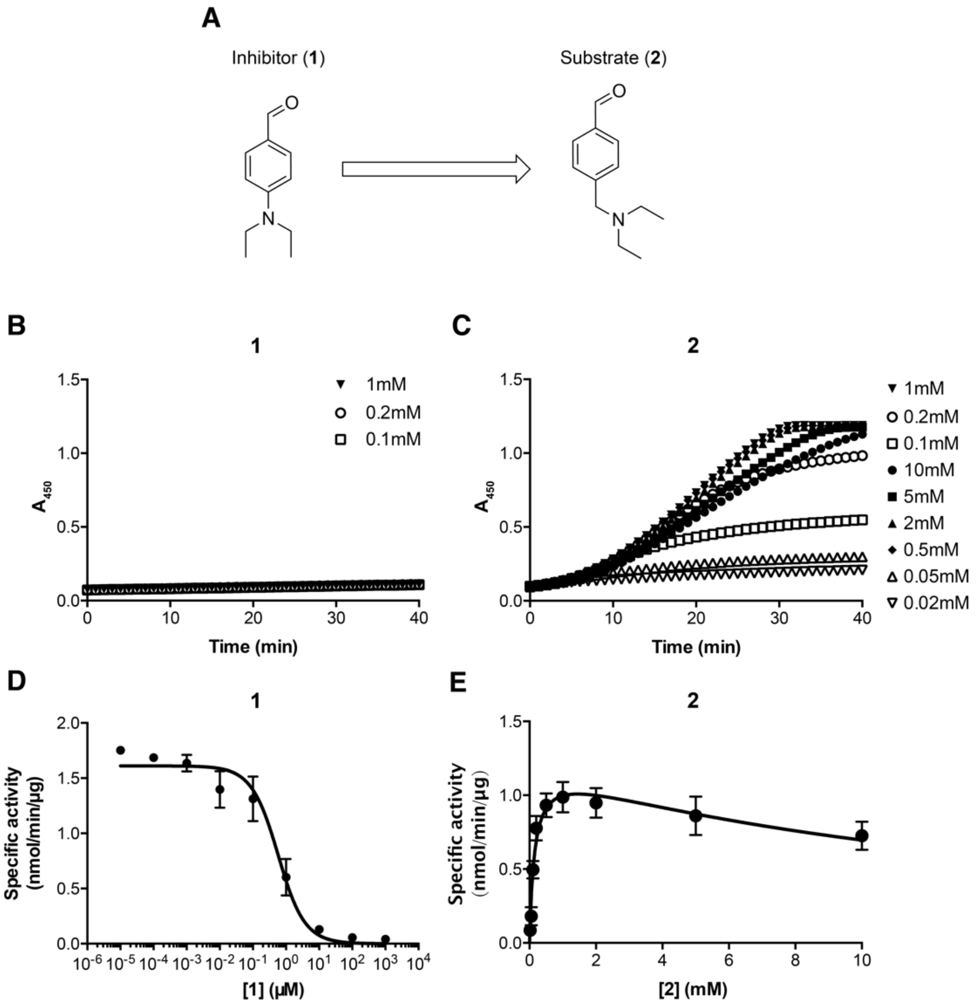

Fig. S1: ALDH1A1 enzyme kinetics, showing the conversion of DEAB **1** into a substrate **2** by decoupling the nitrogen from the aromatic system. A. Conversion of ALDH inhibitor **1** to substrate **2.** B. DEAB (**1**) is a poor substrate for ALDH1A1, with minimal NADH production over the duration of the experiment, as shown by minimal changes in absorbance at 450 nm. C. Decoupling the nitrogen from the aromatic system produced **2**, which was rapidly turned over by ALDH1A1 in a concentration-dependent manner, as shown by an increased absorbance at 450 nm. D. Concentration-dependent inhibition of ALDH1A1 activity by DEAB, **1**. The results were fitted to Equation 2 (solid line). Data are mean ± SD of three independent experiments. E. Michaelis Menten plot, showing the effect of concentration on the initial reaction velocity for compound **2**. The results were fitted to Equation 1 (solid line). Data are mean ± SD of three independent experiments.

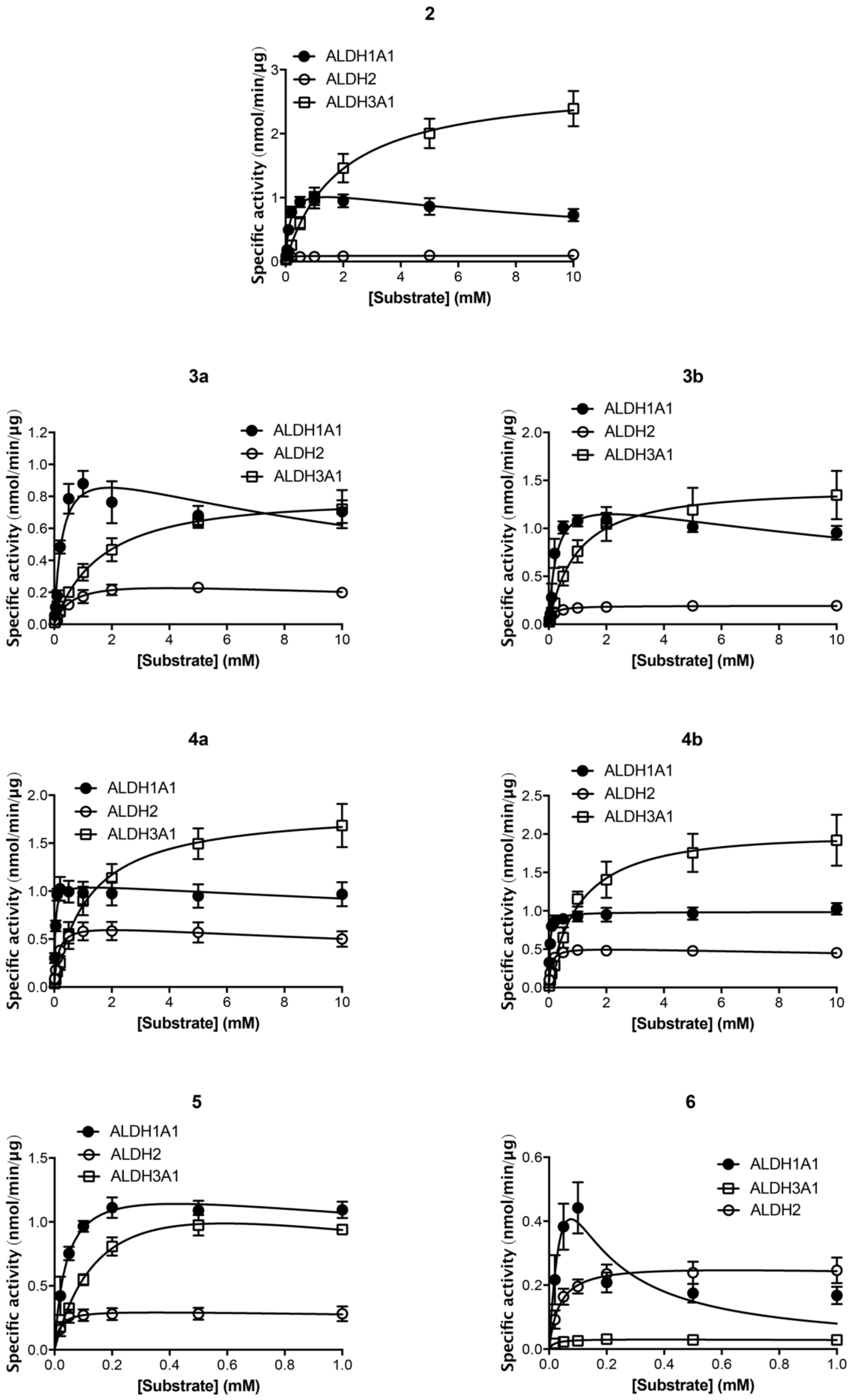

Fig. S2: Effect of substrate concentration for compounds **2**-**6** on the initial reaction velocity determined spectrophotometrically using recombinant human ALDH1A1, ALDH2 and ALDH3A1. Initial reaction velocity was determined by measuring the increase in absorbance at 450 nm at 37 °C. The results were fitted to Equation 1 (solid line). Data are mean ± SD of three independent experiments.

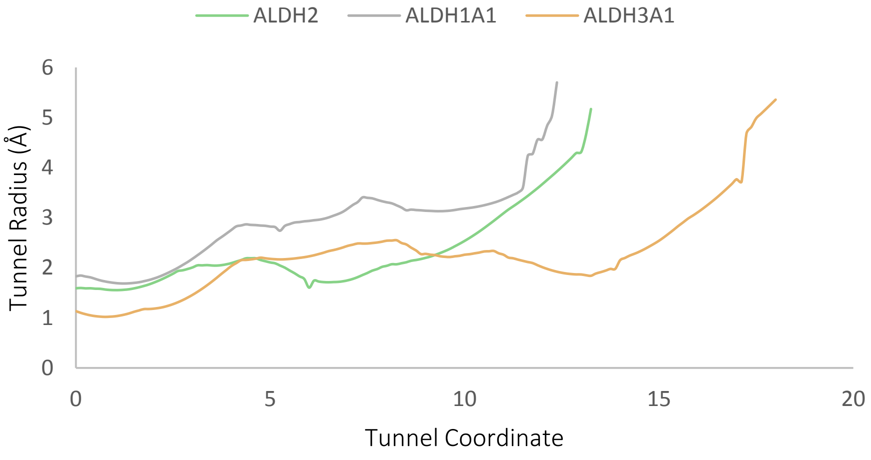

Fig. S3: Tunnel radii of ALDH1A1, ALDH2 and ALDH3A1

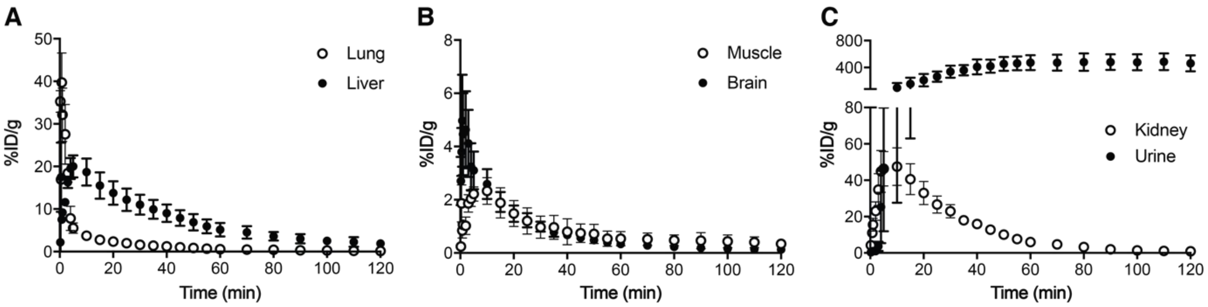

Fig. S4: [^{18}F]**4b** pharmacokinetics in healthy mice. A. The time versus radioactivity curve representing average counts from a dynamic 120 min scan in the lung and liver. B. [^{18}F]**4b** uptake profiles in the muscle and brain. C. Temporal urinary excretion of [^{18}F]**4b** from dynamic PET. Data are means ± SD (n = 3 animals). ID/g, injected dose per g tissue, assuming 1 g/mL.

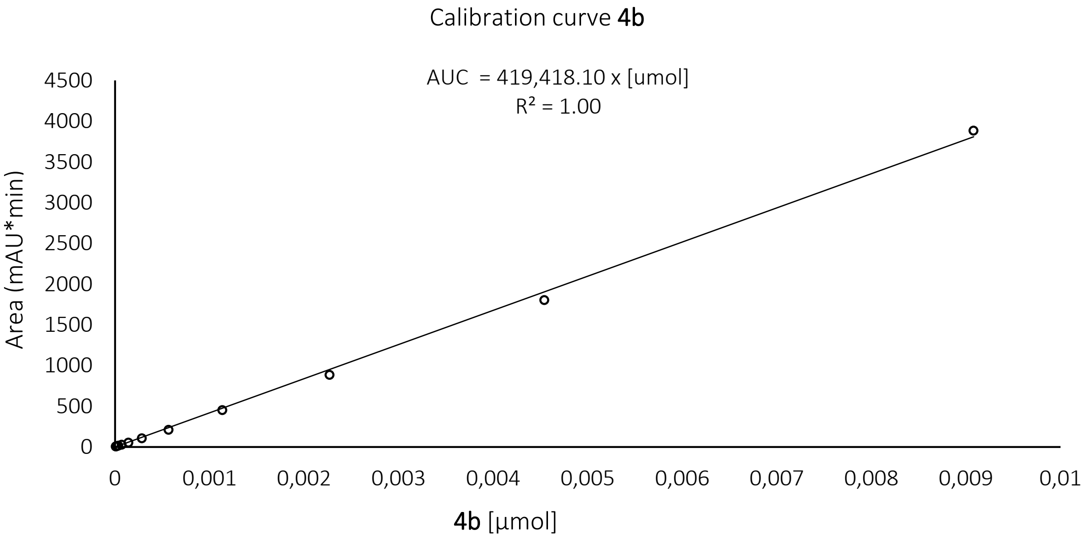

Fig. S5: **4b** calibration curve.

HPLC CHROMATOGRAMS

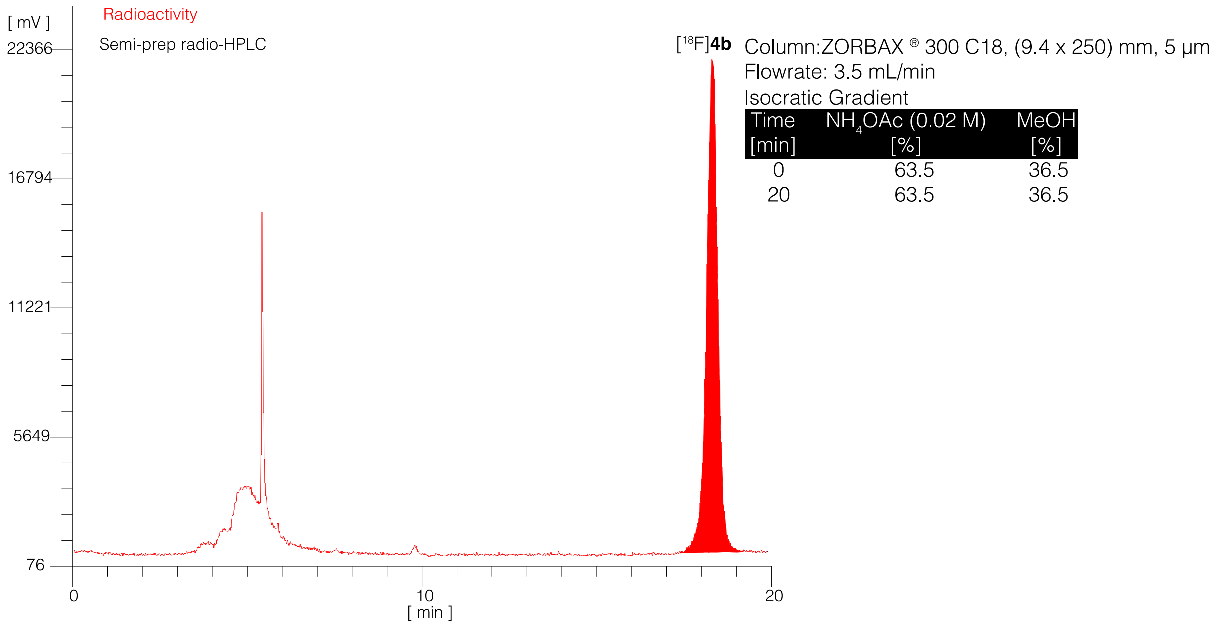

Chromatogram S1: Semi-prep radio-HPLC purification of [^{18}F]4b

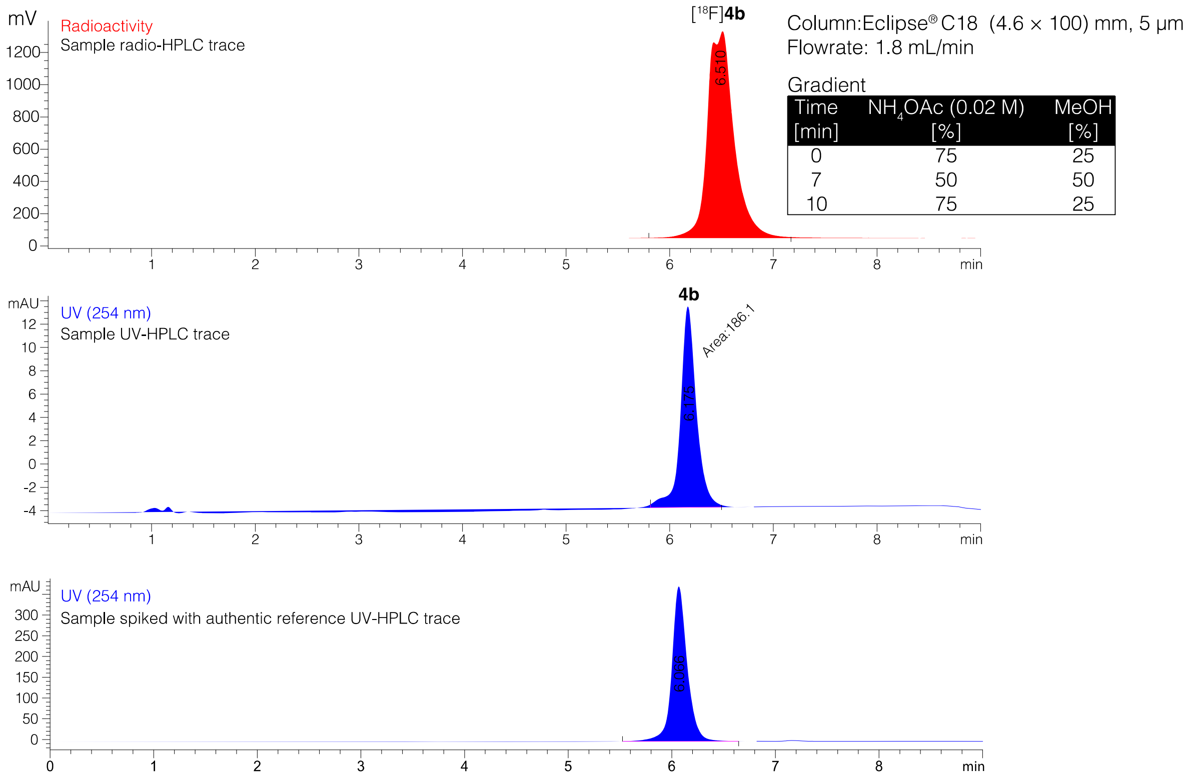

Chromatogram S2: Top: radio-HPLC trace of purified [^{18}F]**4b** on an analytical column. Middle: UV trace of purified [^{18}F]**4b**. Bottom: UV trace of purified [^{18}F]**4b** spiked with **4b** (authentic reference).

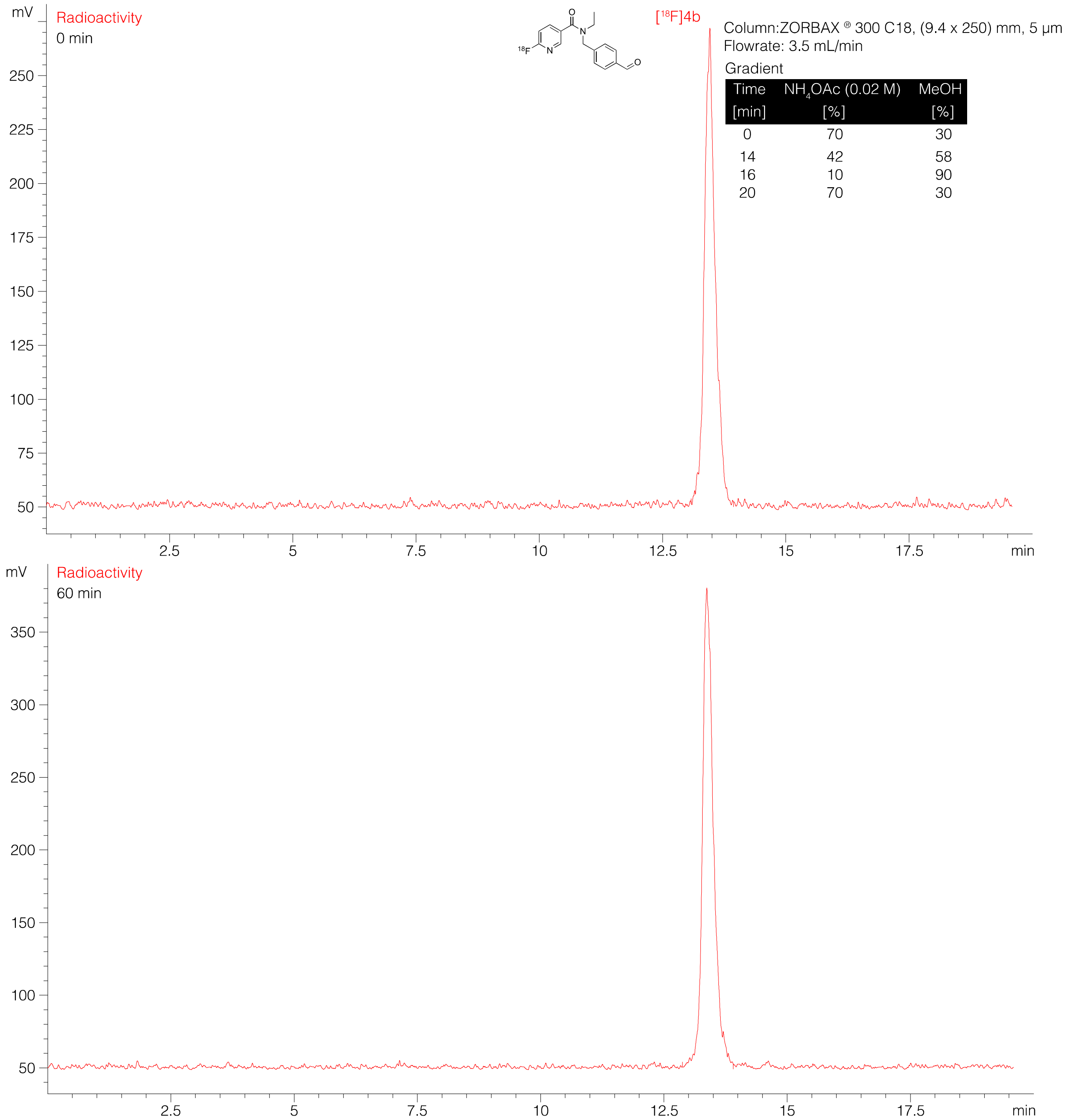

Chromatogram S3: Radio-HPLC chromatograms of [^{18}F]**4b** after incubating in cell media in the absence of cells for 0 min and 60 min at 37°C.

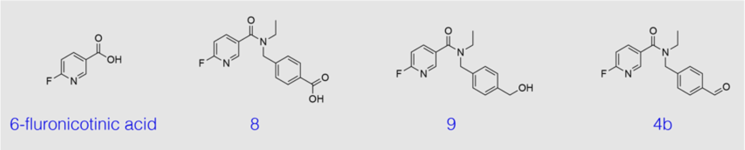

Column:ZORBAX ® 300 C18, (9.4 x 250) mm, 5 µm
Flowrate: 3.5 mL/min

Gradient

Time [min]	NH_4OAc (0.02 M) [%]	MeOH [%]
0	70	30
14	42	58
16	10	90
20	70	30

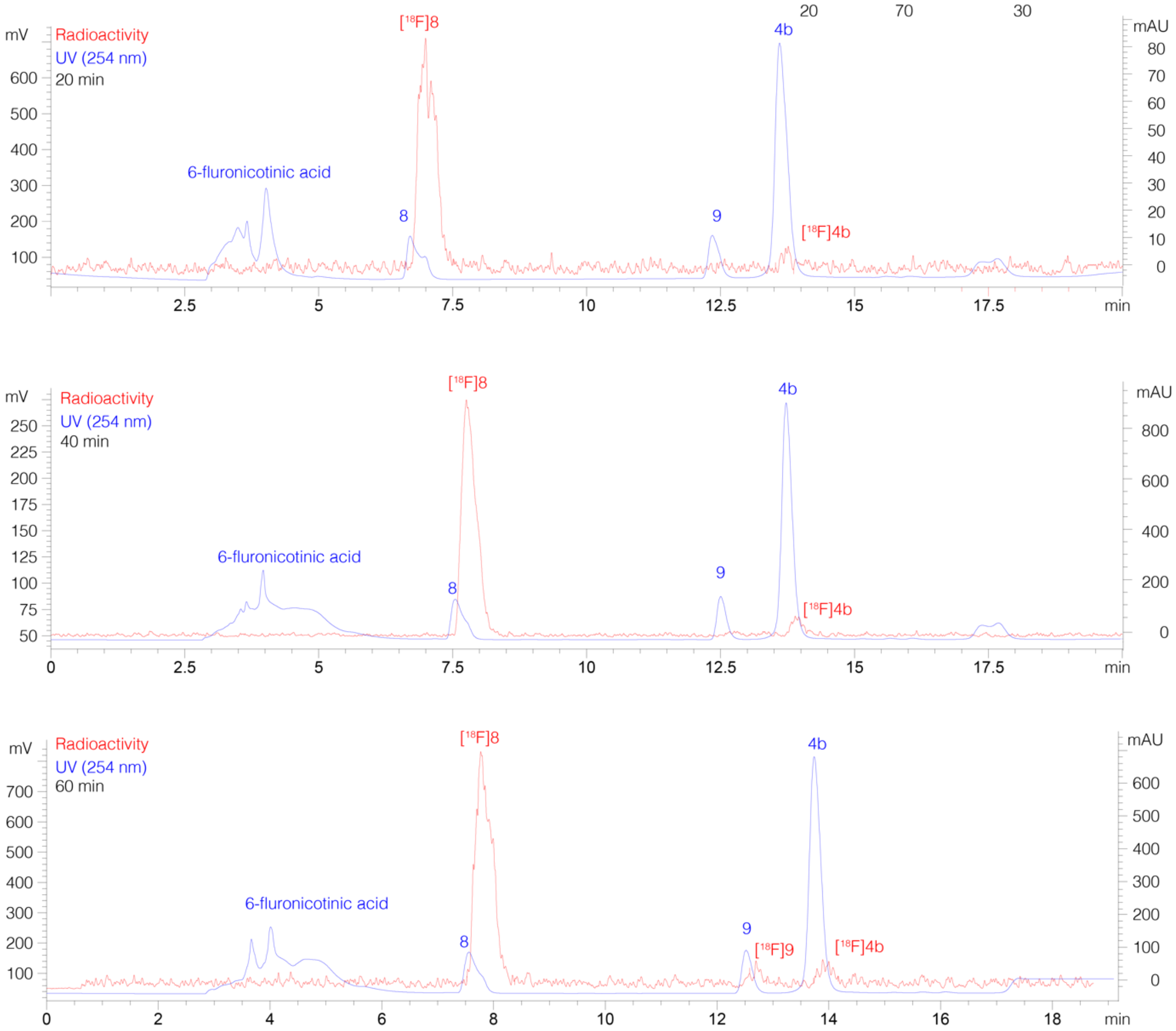

Chromatogram S4: Radio- and UV-HPLC chromatograms from cell lysates (co-injected with authentic 6-fluoronicotinic acid, **8**, **9**, and **4b**) following 20, 40, and 60 min incubation of [^{18}F]**4b**.

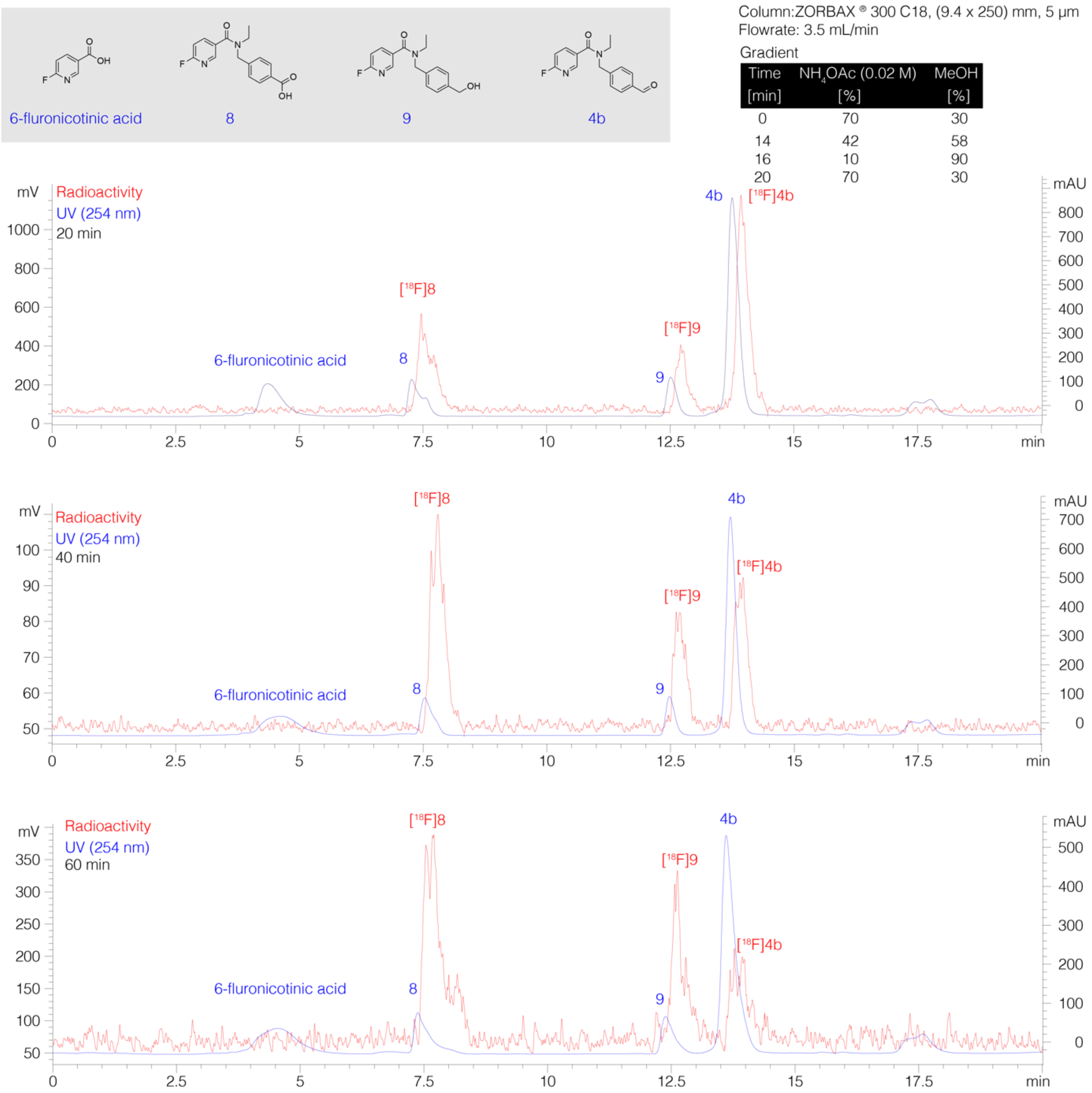

Chromatogram S5: Radio- and UV-HPLC chromatograms from media lysates (co-injected with authentic 6-fluoronicotinic acid, **8**, **9**, and **4b**) following 20, 40, and 60 min incubation of [^{18}F]**4b**.

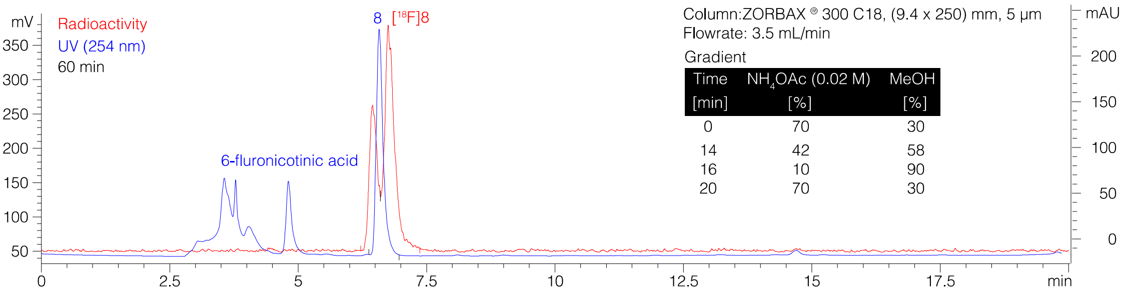

Chromatogram S6: Radio- and UV-HPLC chromatograms from mouse blood analysis (co-injected with authentic 6-fluoronicotinic acid, and **8**) following 60 min incubation of [^{18}F]**4b**.

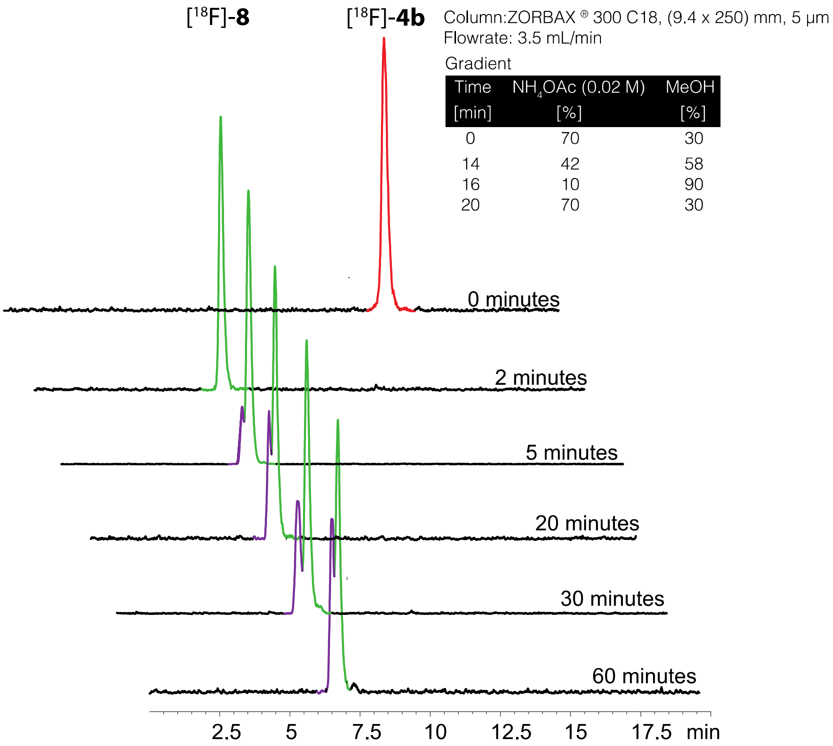

Time [min]	NH_4OAc (0.02 M) [%]	MeOH [%]
0	70	30
14	42	58
16	10	90
20	70	30

Chromatogram S7: [18F]**4b** stability and metabolism. Radio-HPLC traces from the plasma of mice taken at 0, 2, 5, 20, 30, 60 min after tail vein injection of [18F]**4b** (red peak at 13.5 min). Appearance of the corresponding carboxylic acid, [18F]**8**, (green) and an unknown metabolite (purple) were observed following injection.

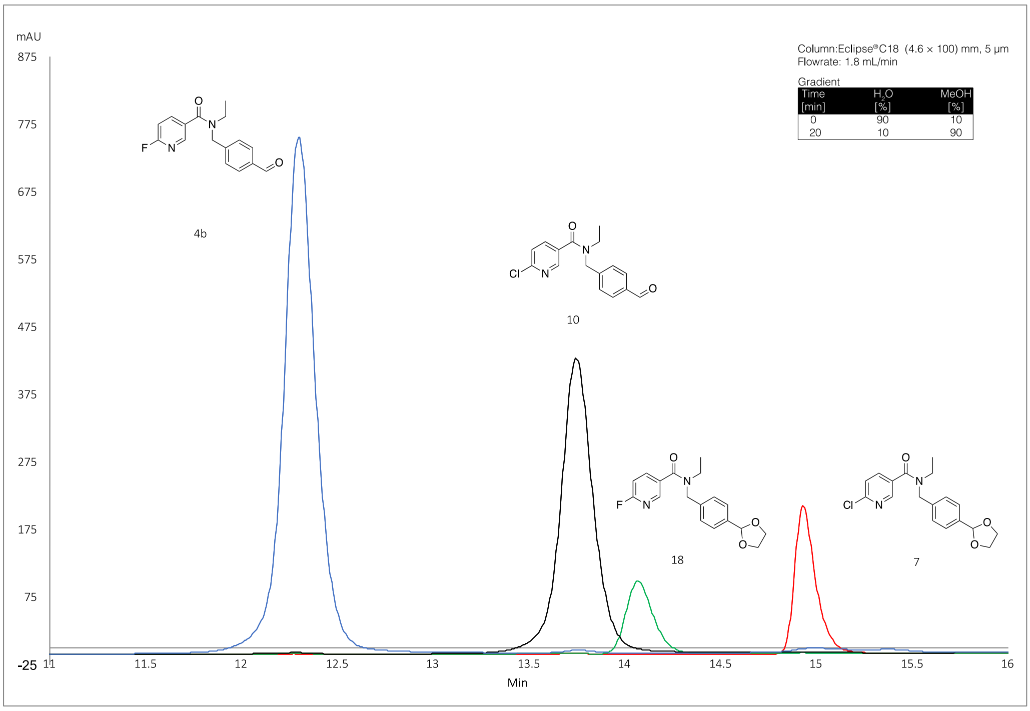

Time [min]	H_2O [%]	MeOH [%]
0	90	10
20	10	90

Chromatogram S8: UV-HPLC chromatograms showing the separation of **4b** from **7, 10,** and **18.**

NMR SPECTRA

^{19}F NMR (282 MHz, $CDCl_3$, 298K) of 4-(1,3-dioxolan-2-yl)-3-fluorobenzaldehyde **12**

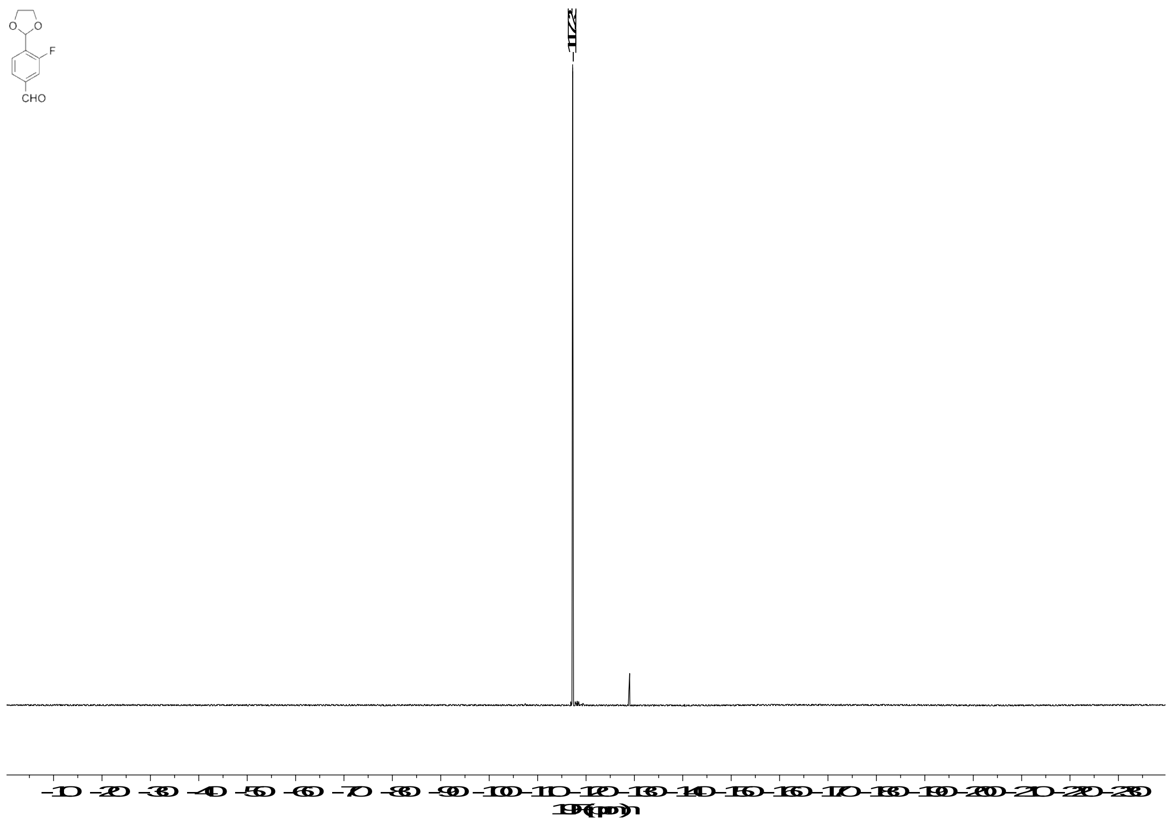

^{1}H NMR (300 MHz, $CDCl_3$, 298K) of 4-(1,3-dioxolan-2-yl)-3-fluorobenzaldehyde **12**

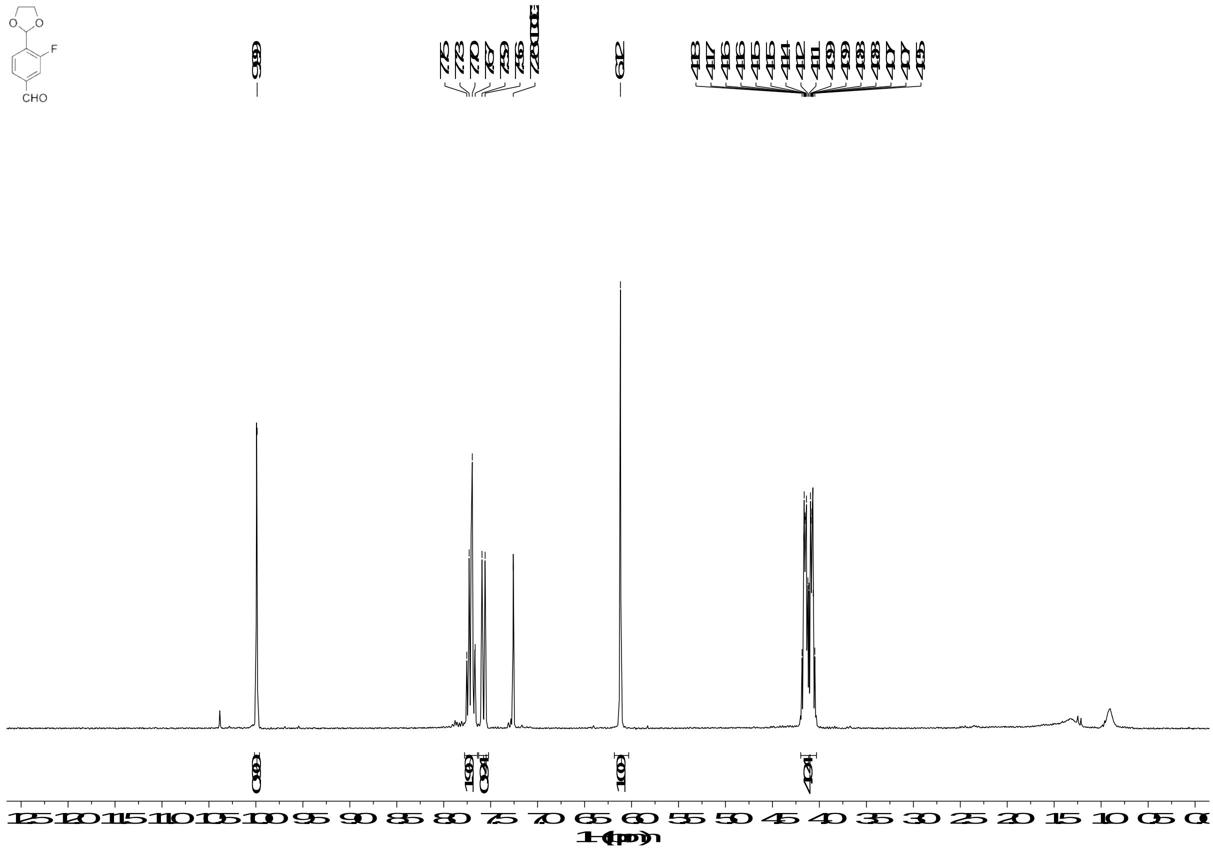

^{13}C NMR (75 MHz, $CDCl_3$, 298K) of 4-(1,3-dioxolan-2-yl)-3-fluorobenzaldehyde **12**

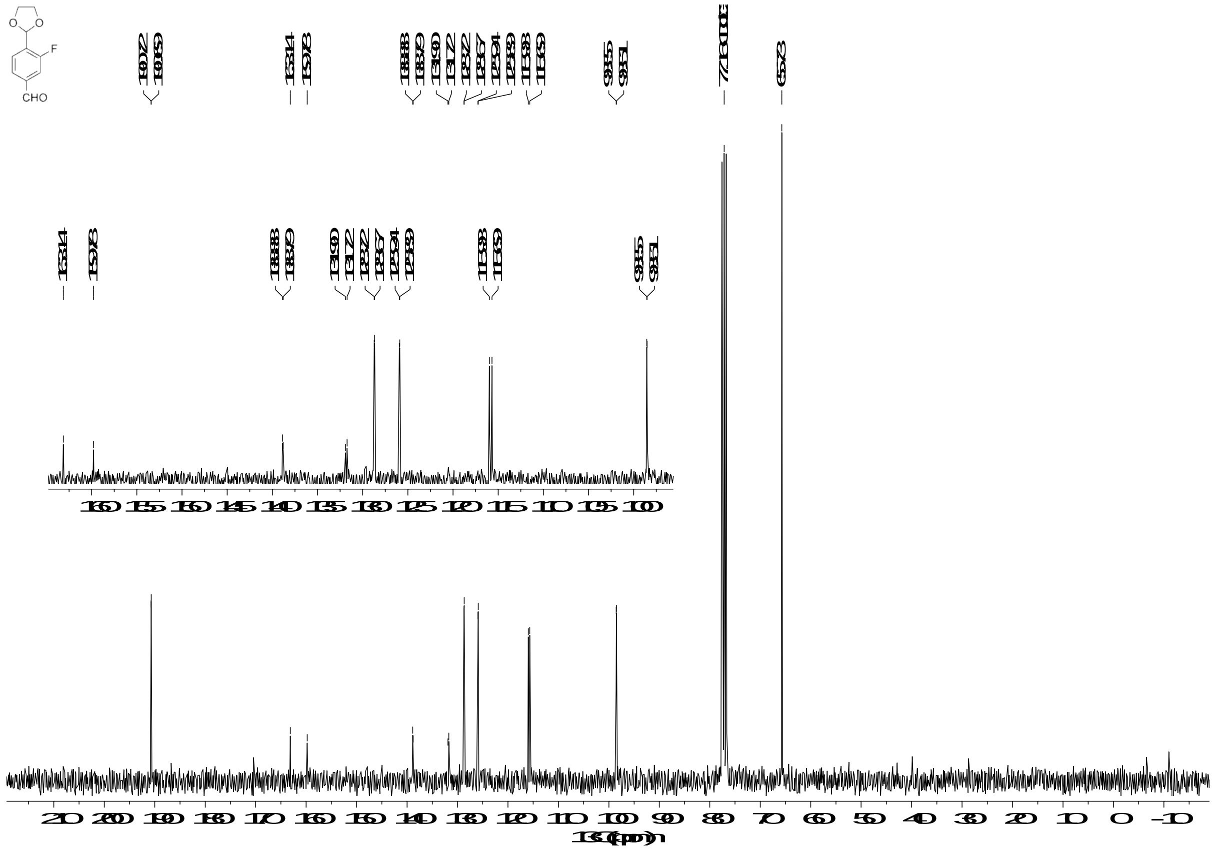

^{19}F NMR (282 MHz, $CDCl_3$, 298K) of 4-((diethylamino)methyl)-2-fluorobenzaldehyde **3a**

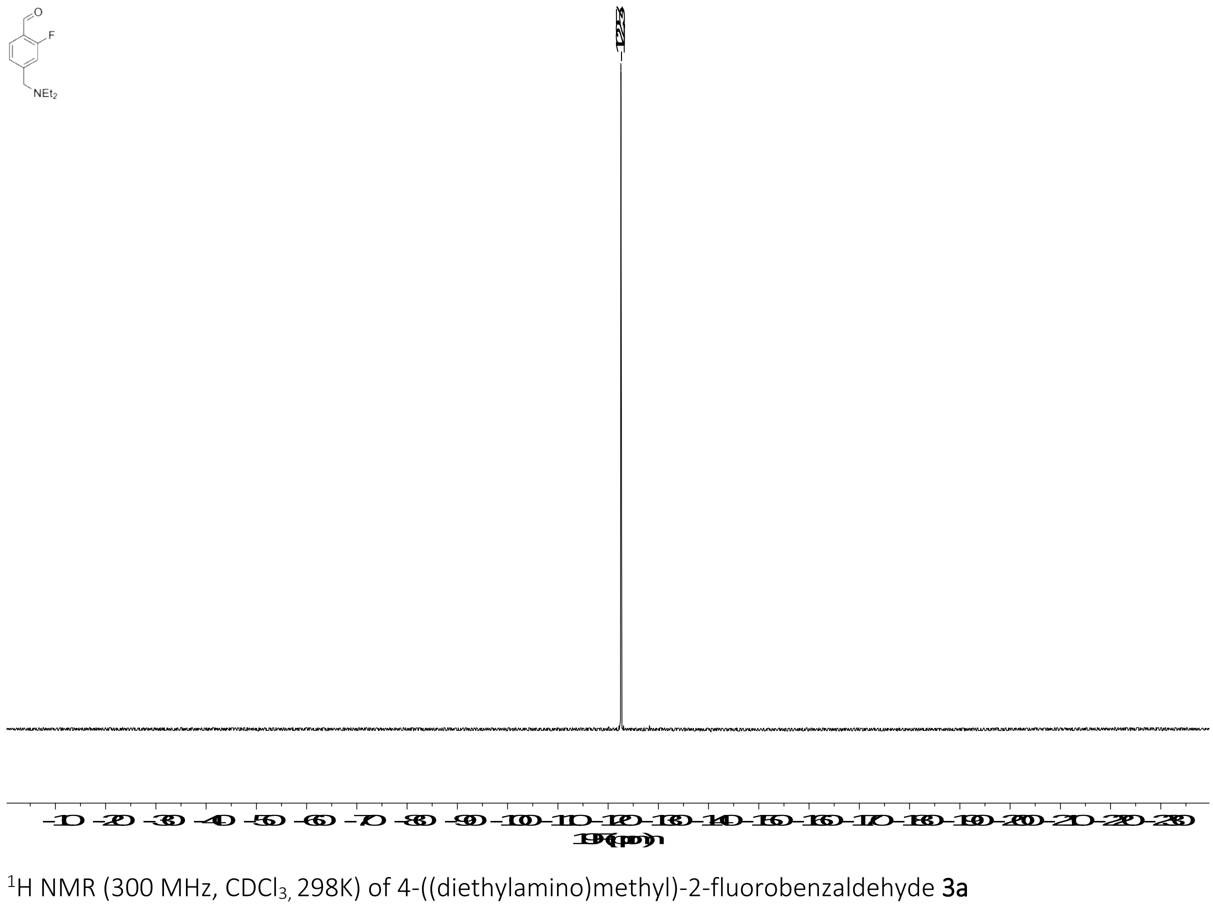

^{1}H NMR (300 MHz, $CDCl_3$, 298K) of 4-((diethylamino)methyl)-2-fluorobenzaldehyde **3a**

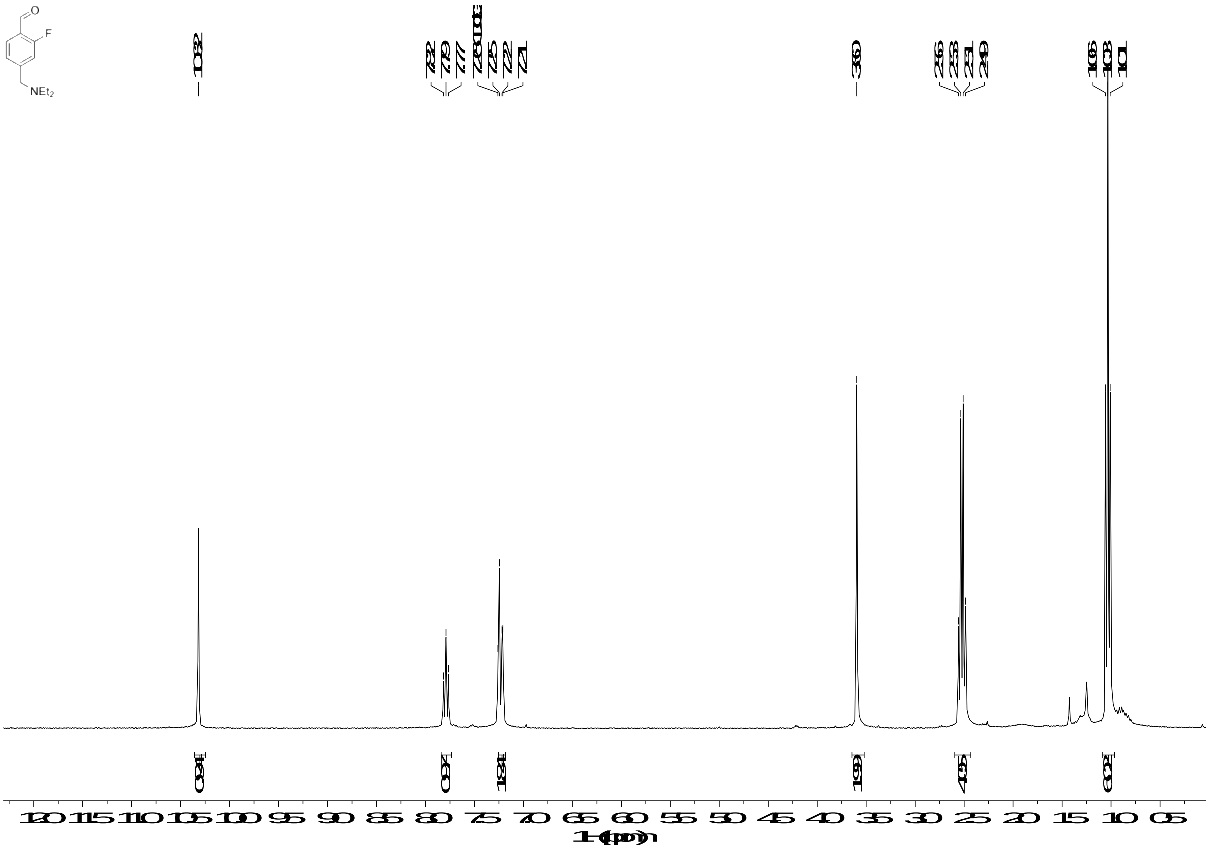

^{13}C NMR (75 MHz, $CDCl_3$, 298K) of 4-((diethylamino)methyl)-2-fluorobenzaldehyde **3a**

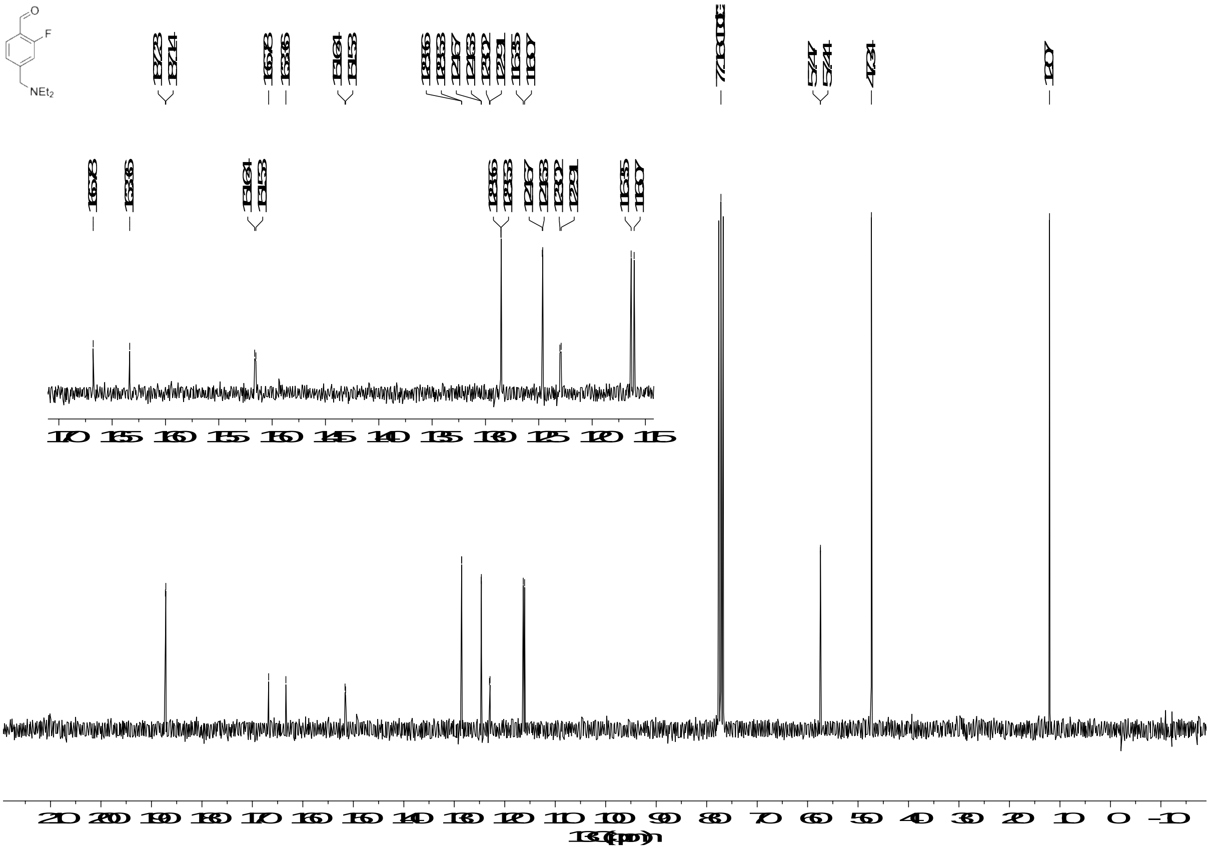

^{19}F NMR (282 MHz, $CDCl_3$, 298K) of 4-(bromomethyl)-3-fluorobenzaldehyde **14**

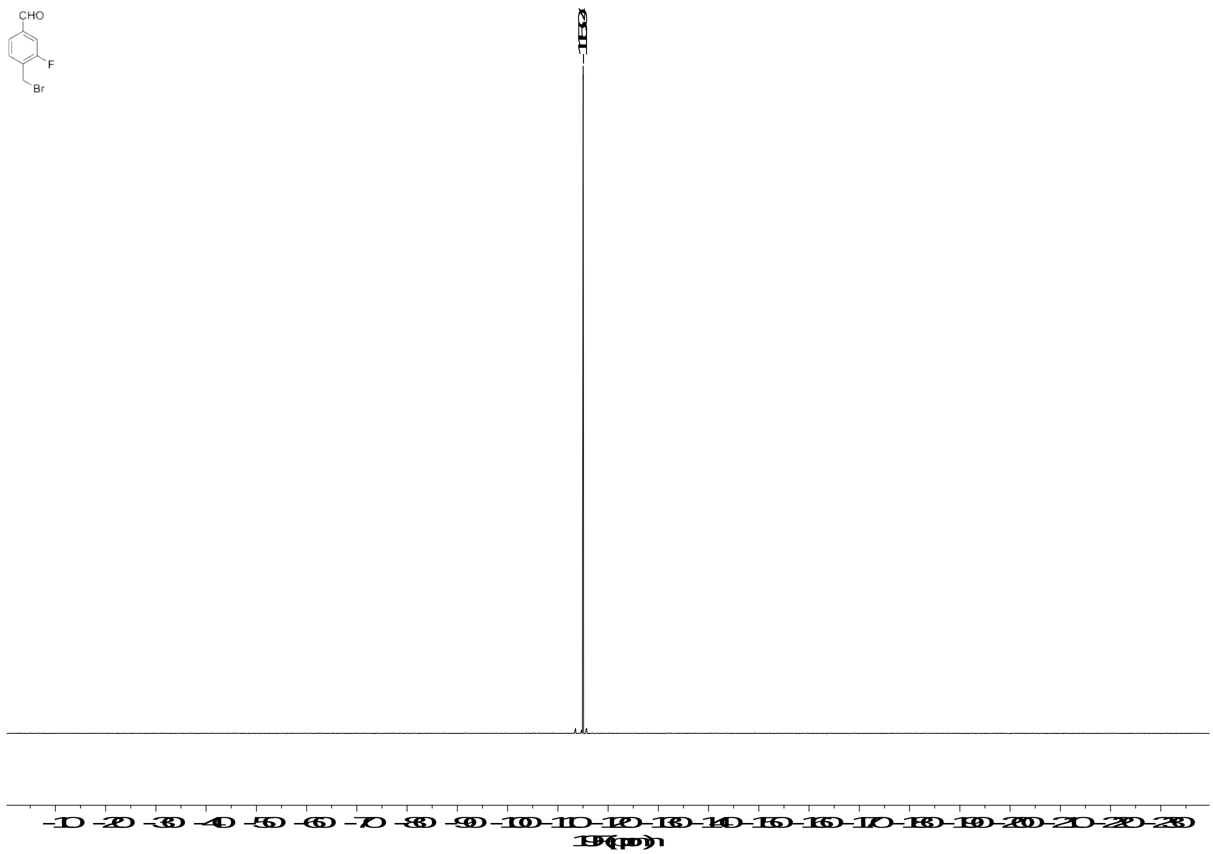

^{1}H NMR (300 MHz, $CDCl_3$, 298K) of 4-(bromomethyl)-3-fluorobenzaldehyde **14**

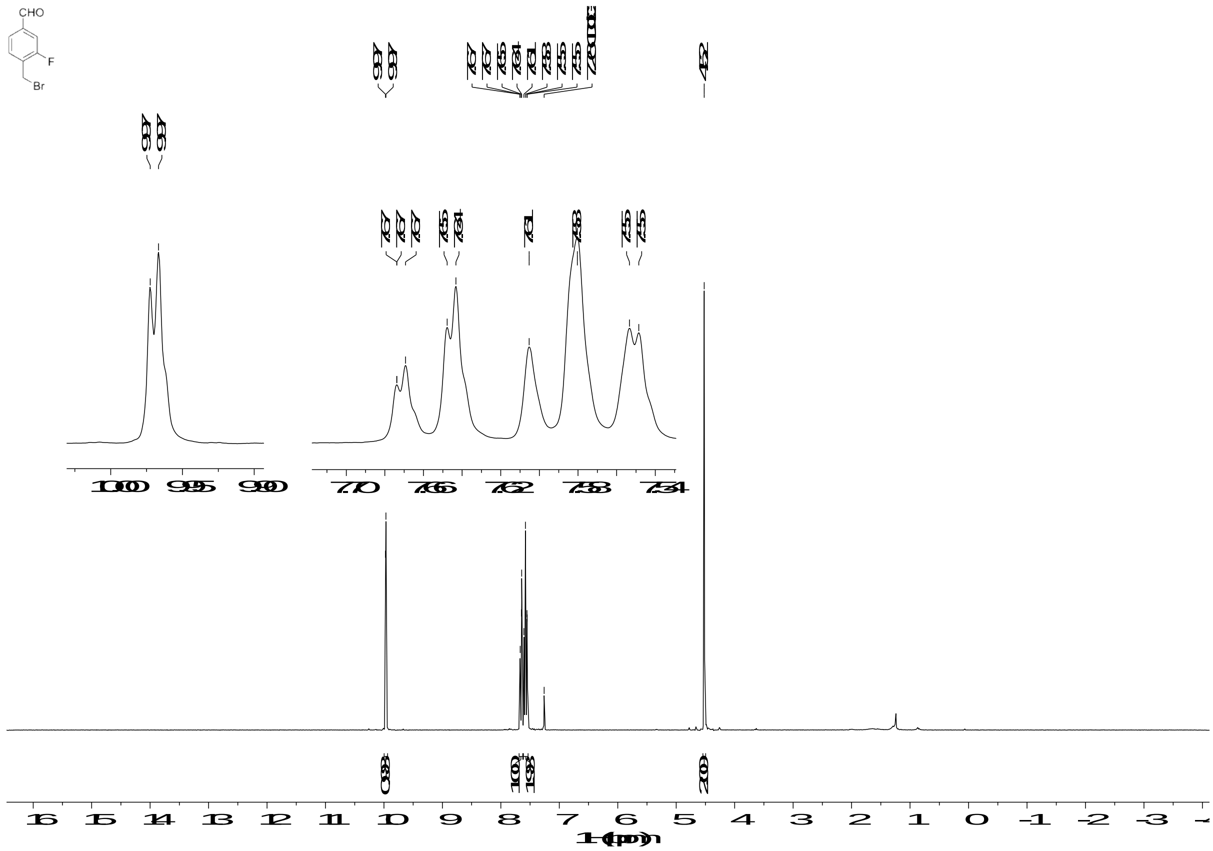

^{13}C NMR (75 MHz, $CDCl_3$, 298K) of 4-(bromomethyl)-3-fluorobenzaldehyde **14**

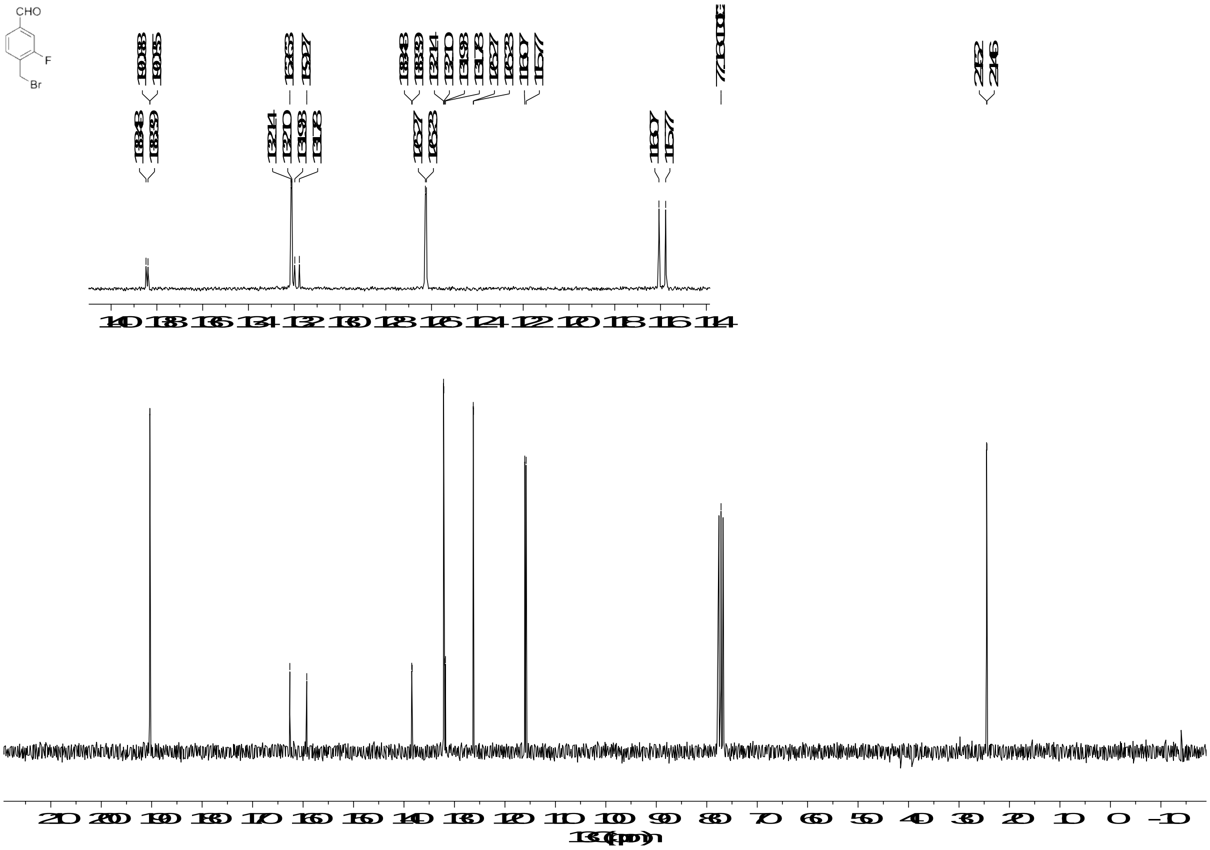

^{19}F NMR (282 MHz, $CDCl_3$, 298K) of 4-((diethylamino)methyl)-3-fluorobenzaldehyde **3b**

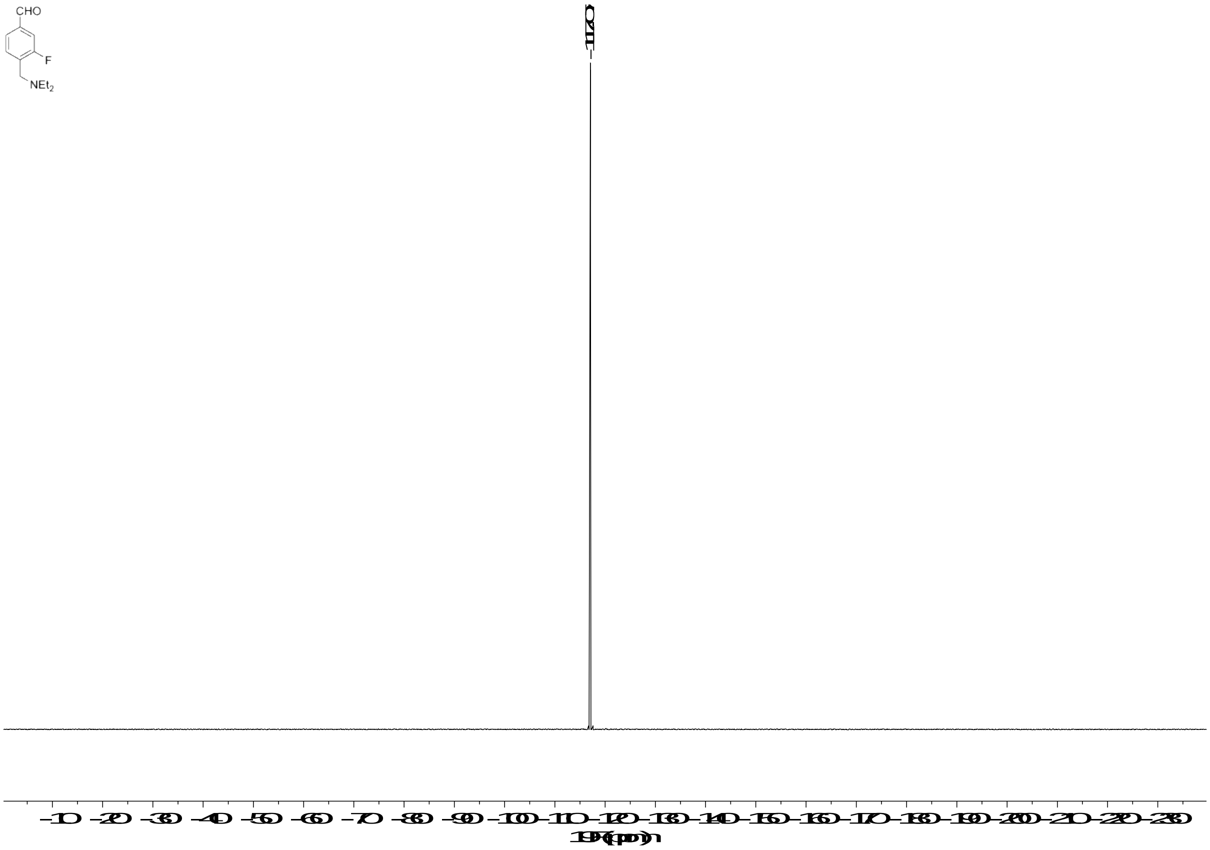

^{1}H NMR (300 MHz, $CDCl_3$, 298K) of 4-((diethylamino)methyl)-3-fluorobenzaldehyde **3b**

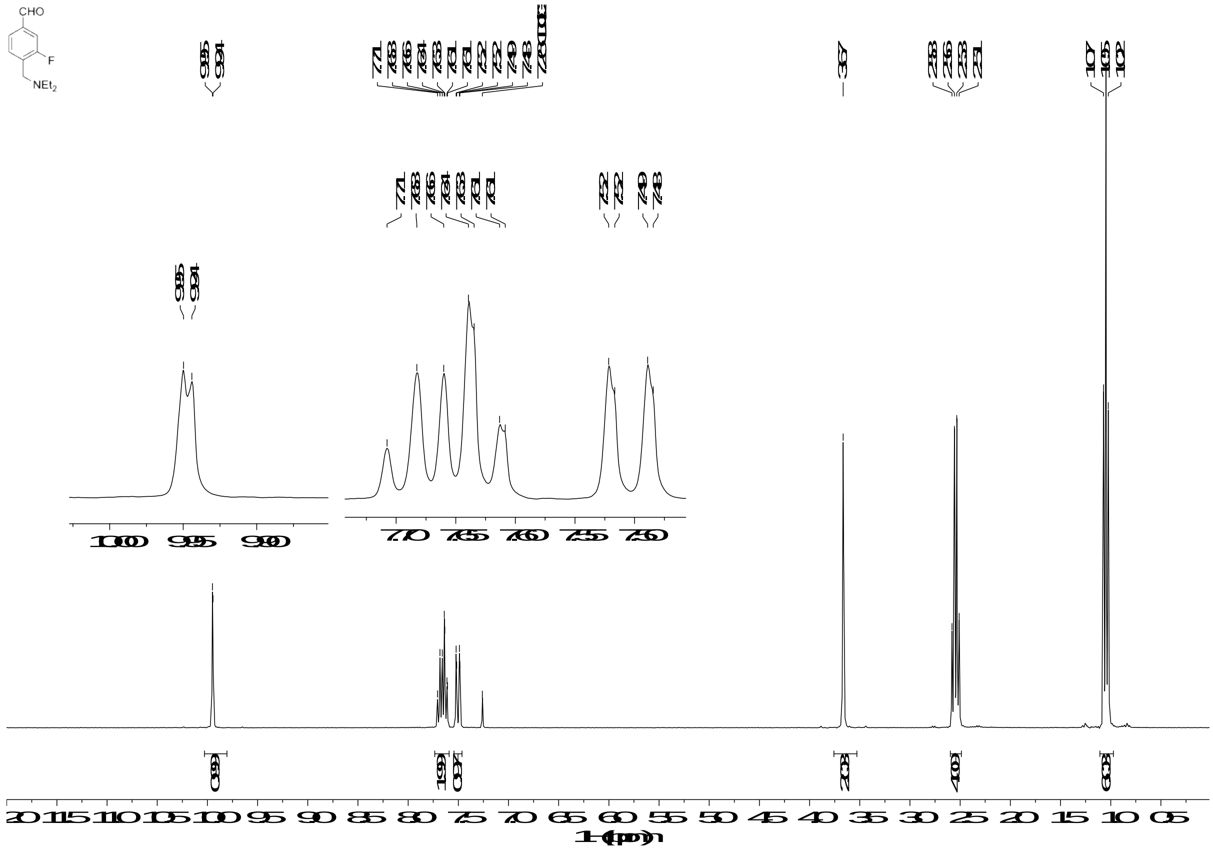

^{13}C NMR (75 MHz, $CDCl_3$, 298K) of 4-((diethylamino)methyl)-3-fluorobenzaldehyde **3b**

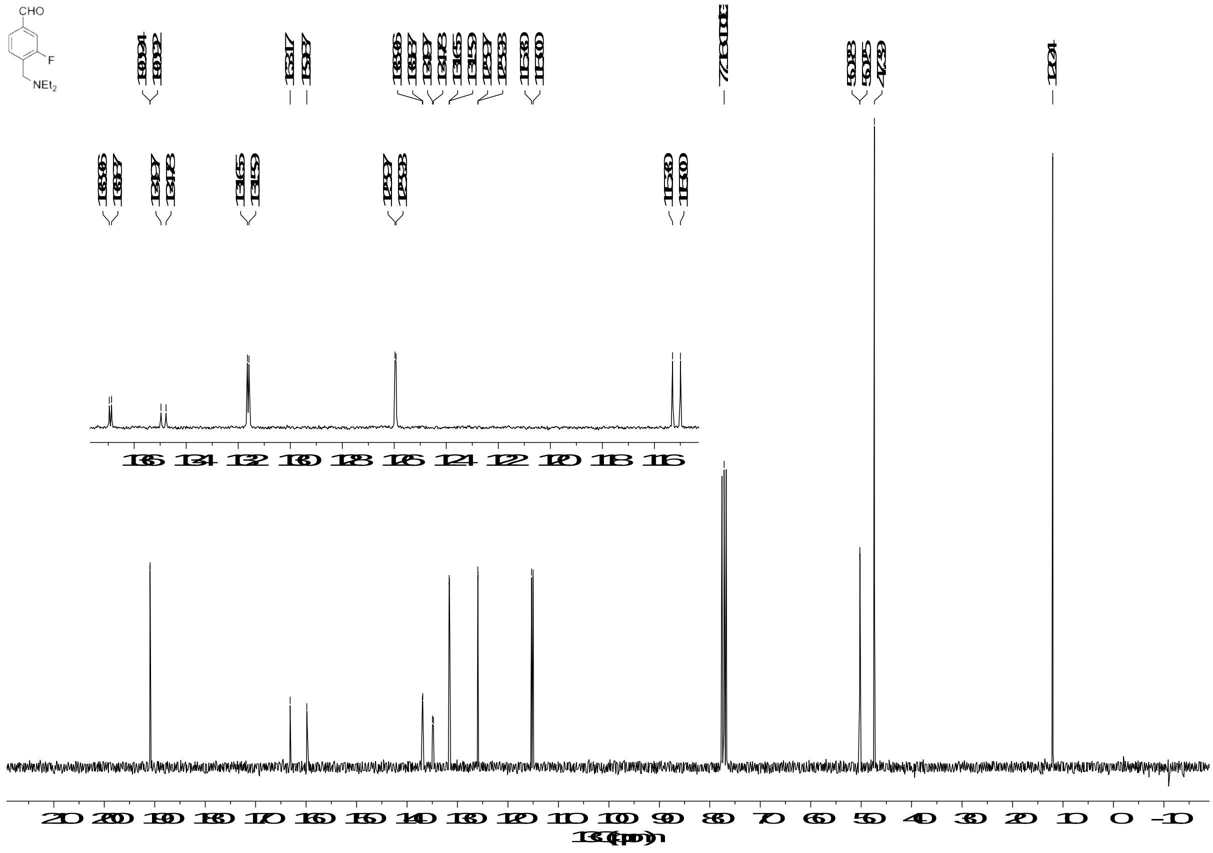

^{1}H NMR (600 MHz, $CDCl_3$, 298K) of *N*-(4-(1,3-dioxolan-2-yl)benzyl)ethanamine **A**

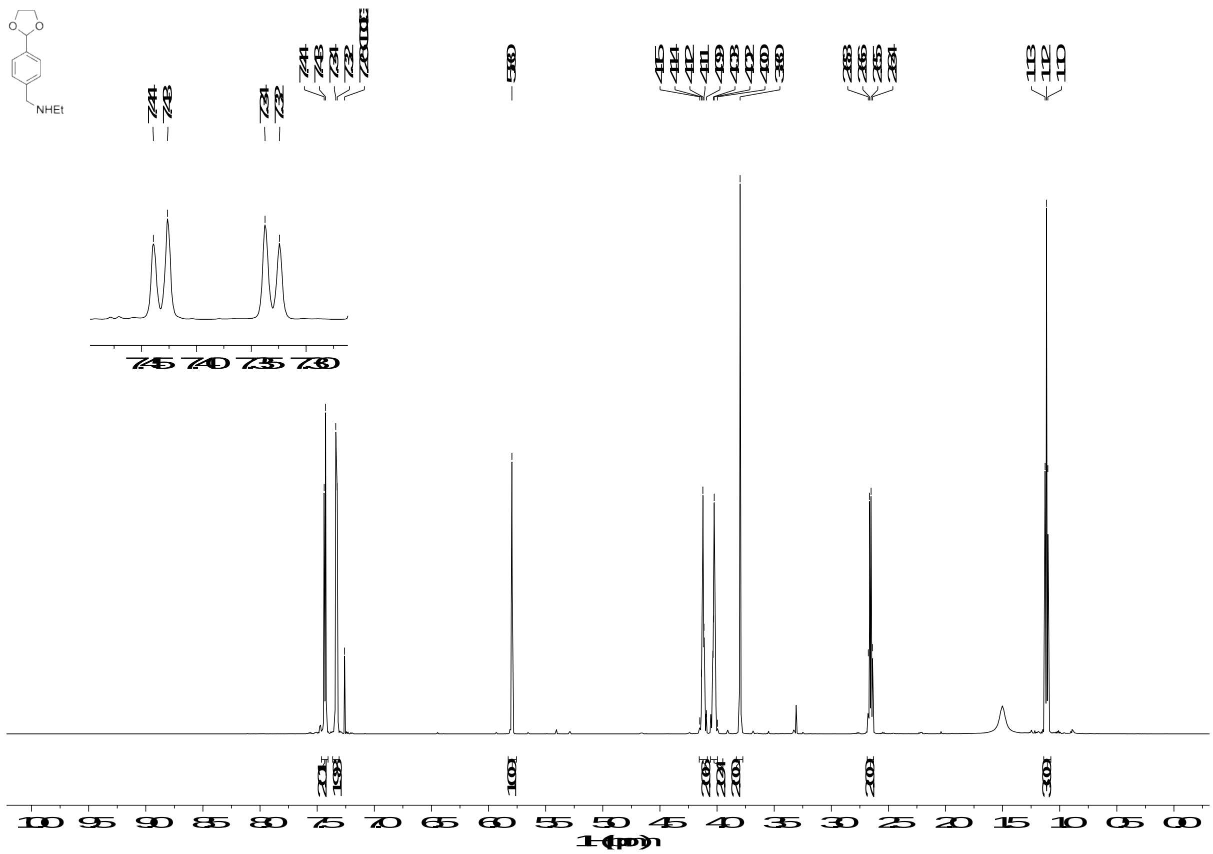

^{13}C NMR (150 MHz, $CDCl_3$, 298K) of *N*-(4-(1,3-dioxolan-2-yl)benzyl)ethanamine **A**

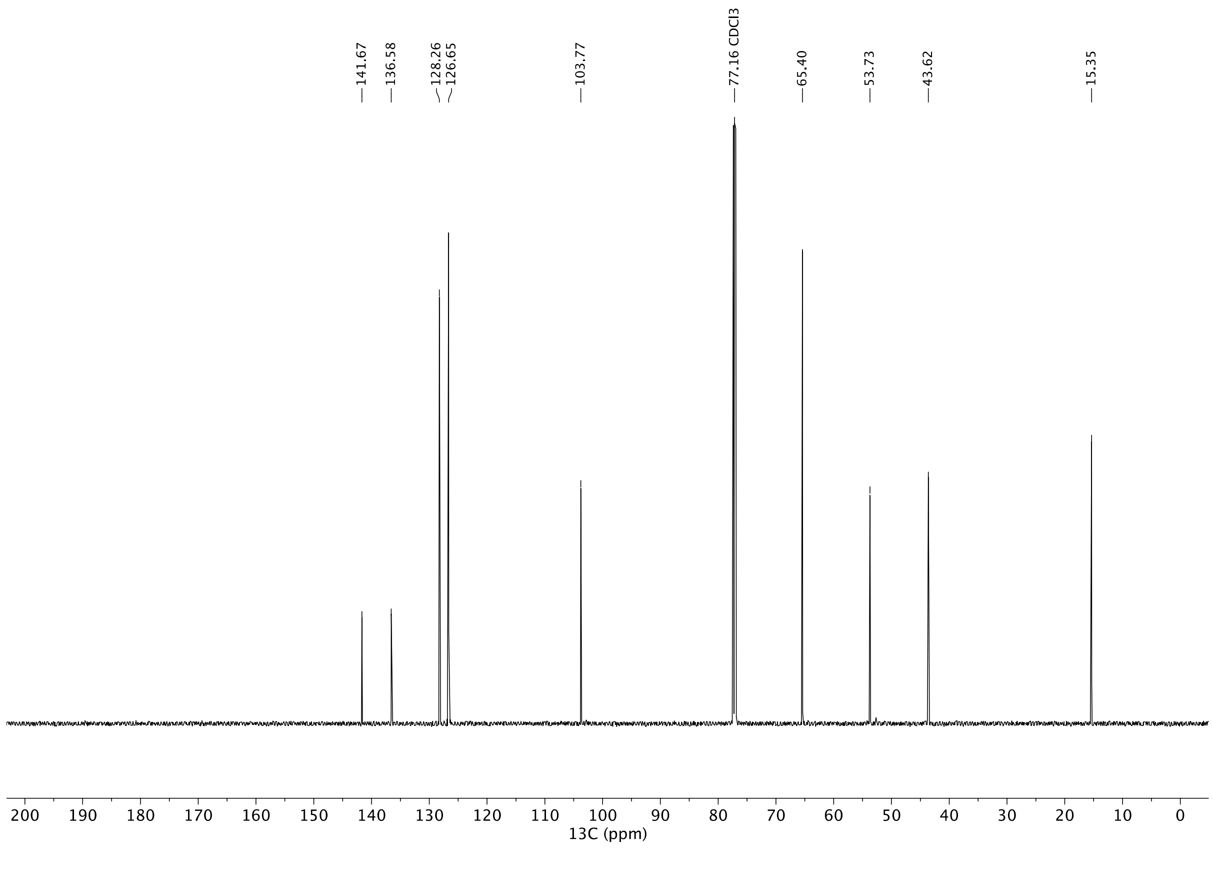

^{1}H NMR (300 MHz, $CDCl_3$, 298K) of *N*-(4-(1,3-dioxan-2-yl)benzyl)ethanamine **B**

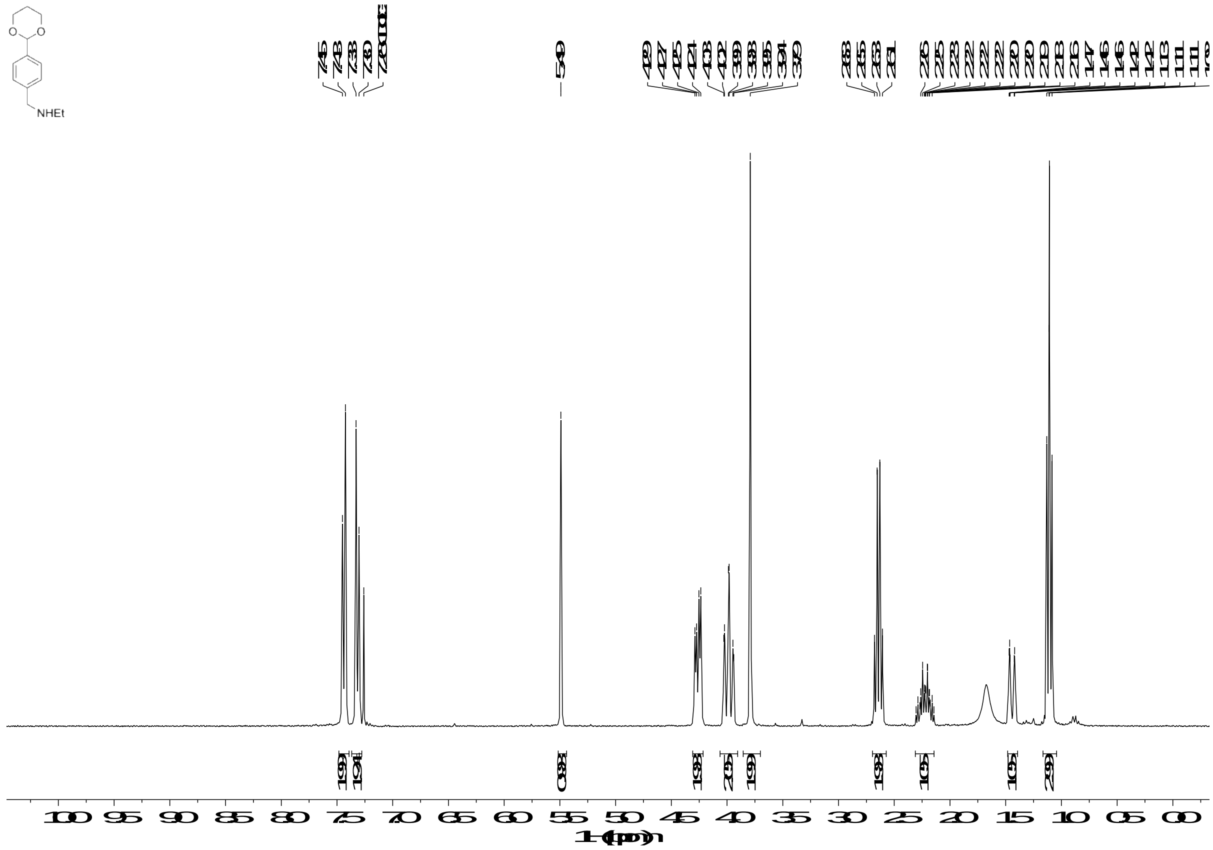

^{13}C NMR (75 MHz, $CDCl_3$, 298K) of *N*-(4-(1,3-dioxan-2-yl)benzyl)ethanamine **B**

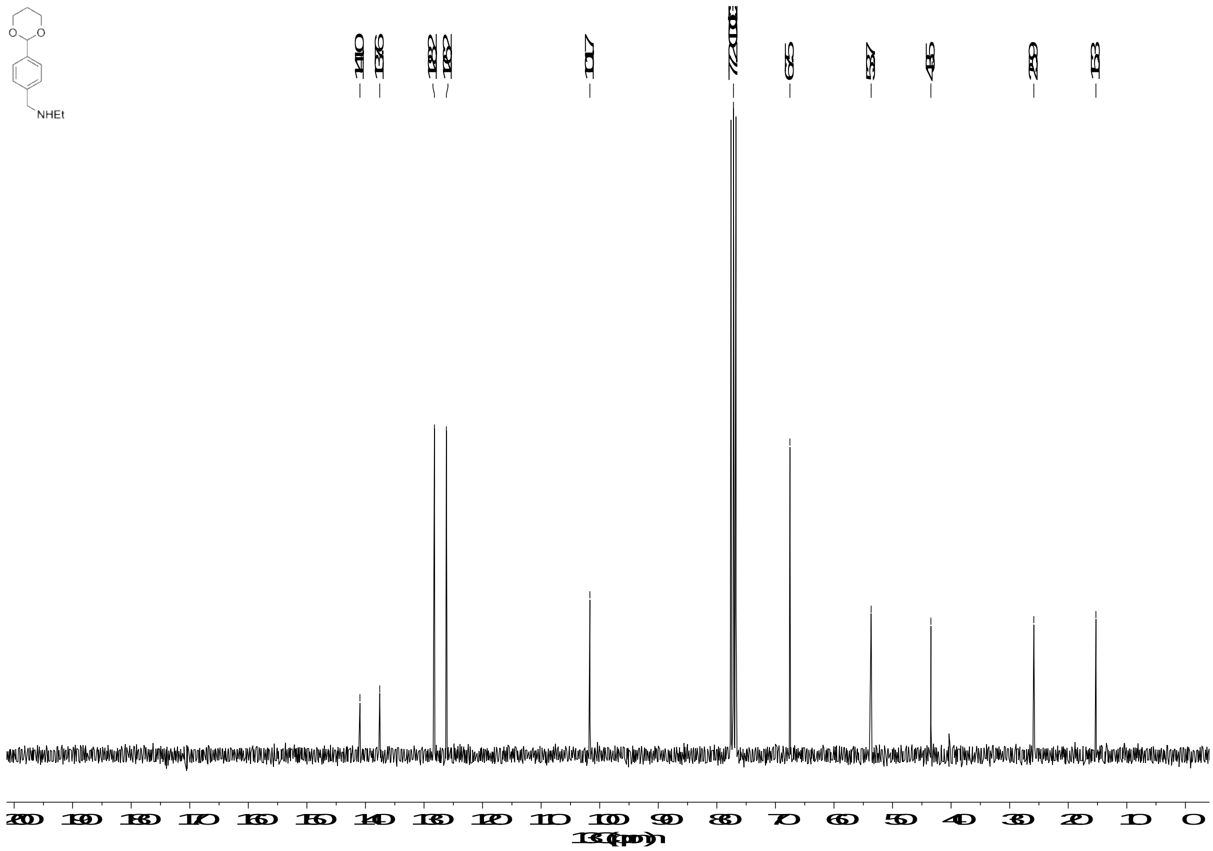

^{19}F NMR (282 MHz, $CDCl_3$, 298K) of *N*-(4-(1,3-dioxan-2-yl)benzyl)-*N*-ethyl-5-fluoronicotinamide **16**

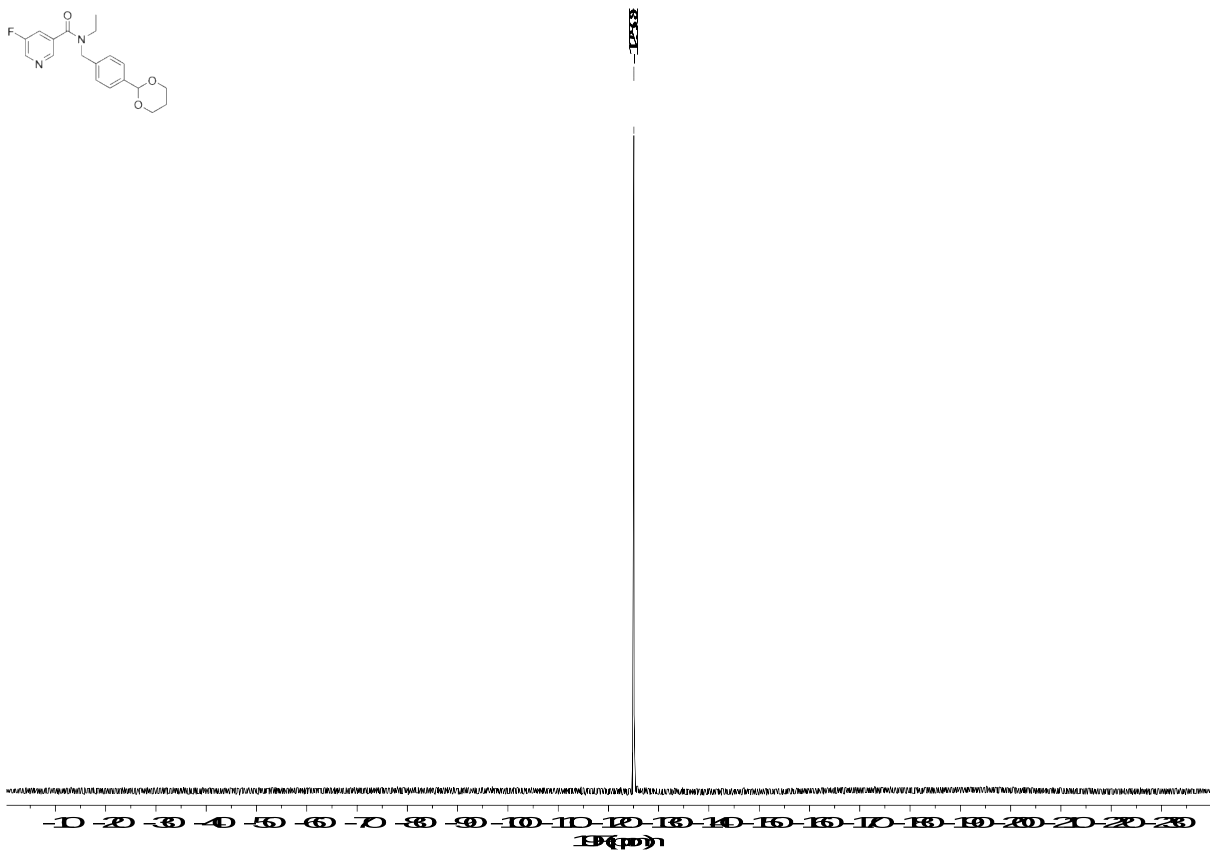

^{1}H NMR (500 MHz, $CDCl_3$, 298K) of *N*-(4-(1,3-dioxan-2-yl)benzyl)-*N*-ethyl-5-fluoronicotinamide **16**

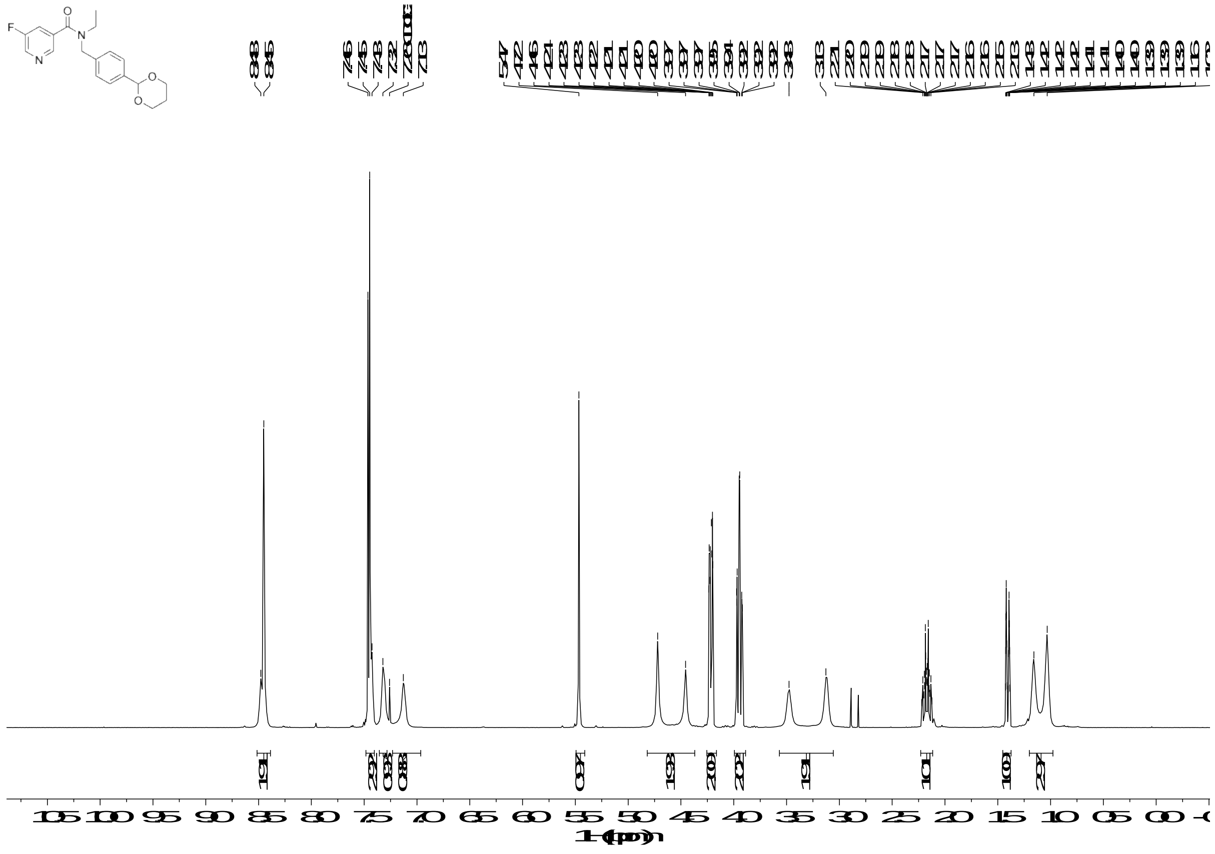

^{13}C NMR (125 MHz, $CDCl_3$, 298K) of *N*-(4-(1,3-dioxan-2-yl)benzyl)-*N*-ethyl-5-fluoronicotinamide **16**

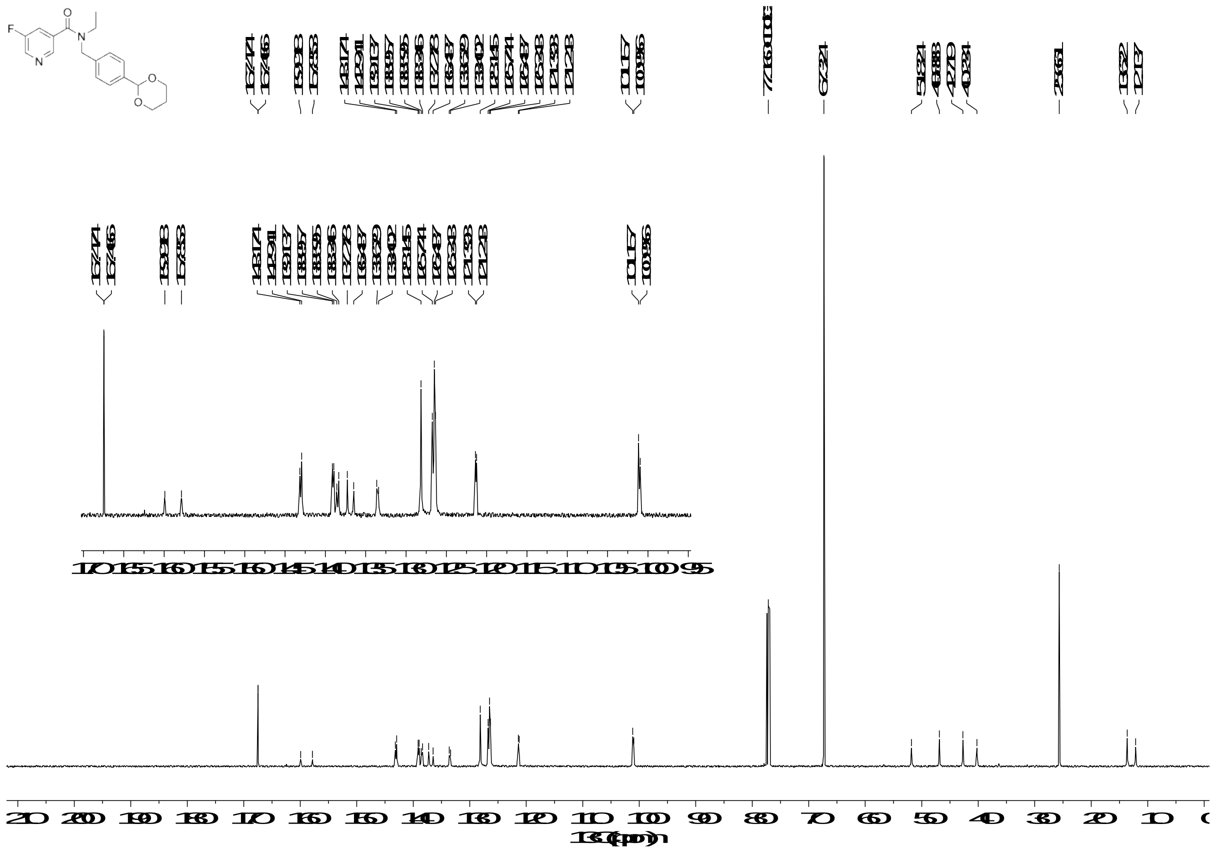

^{19}F NMR (282 MHz, $CDCl_3$, 298K) of *N*-ethyl-5-fluoro-N-(4-formylbenzyl)nicotinamide **4b**

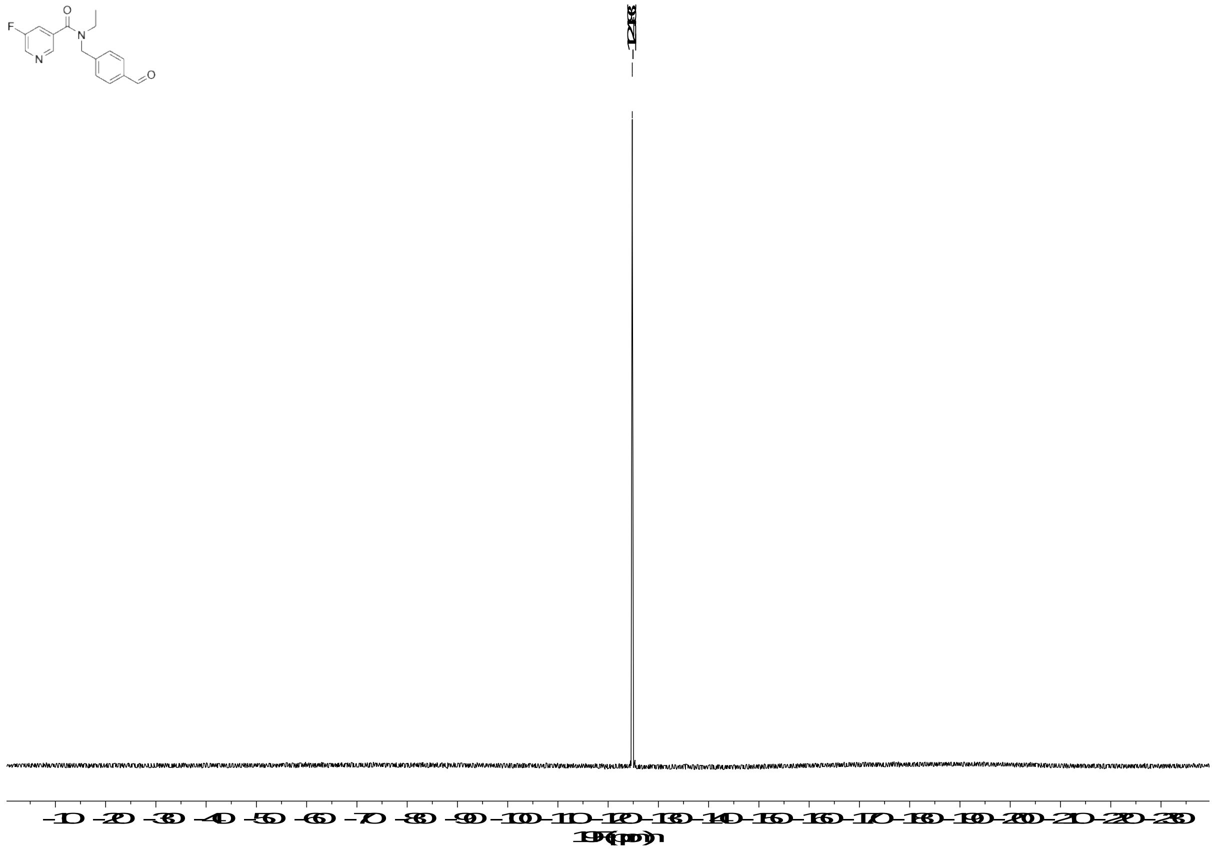

^{1}H NMR (600 MHz, $CDCl_3$, 298K) of *N*-ethyl-5-fluoro-N-(4-formylbenzyl)nicotinamide **4a**

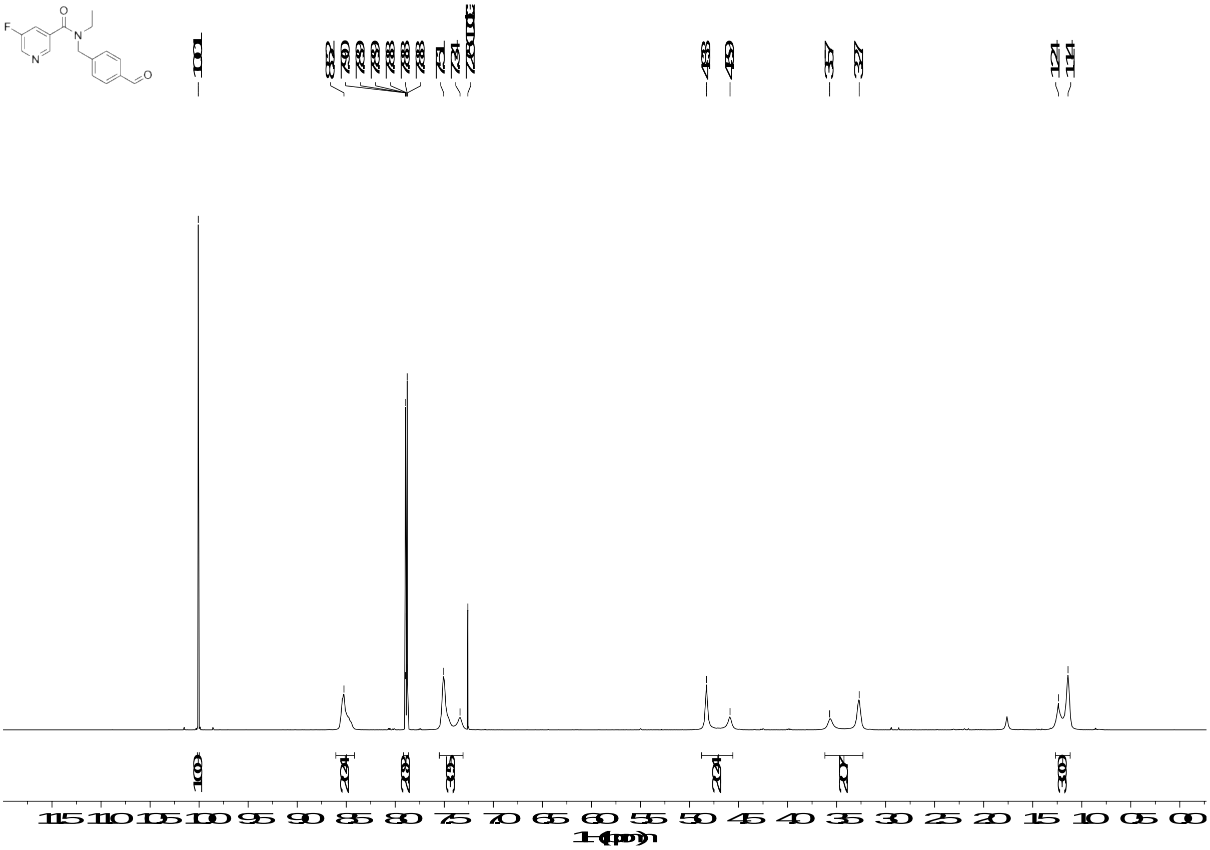

^{13}C NMR (150 MHz, $CDCl_3$, 298K) of *N*-ethyl-5-fluoro-N-(4-formylbenzyl)nicotinamide **4a**

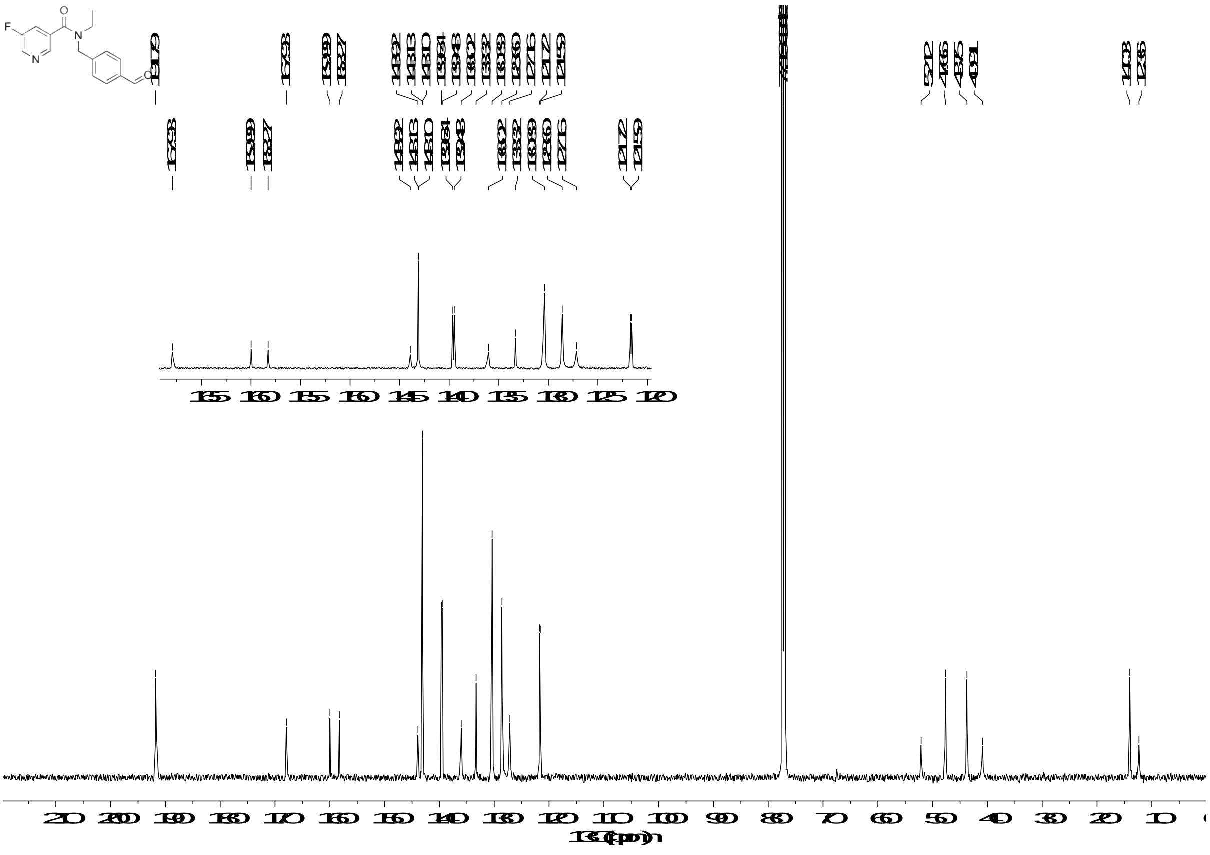

^{19}F NMR (282 MHz, DMSO-d_6, 298K) of *N*-(4-(1,3-dioxolan-2-yl)benzyl)-N-ethyl-6-fluoronicotinamide **18**

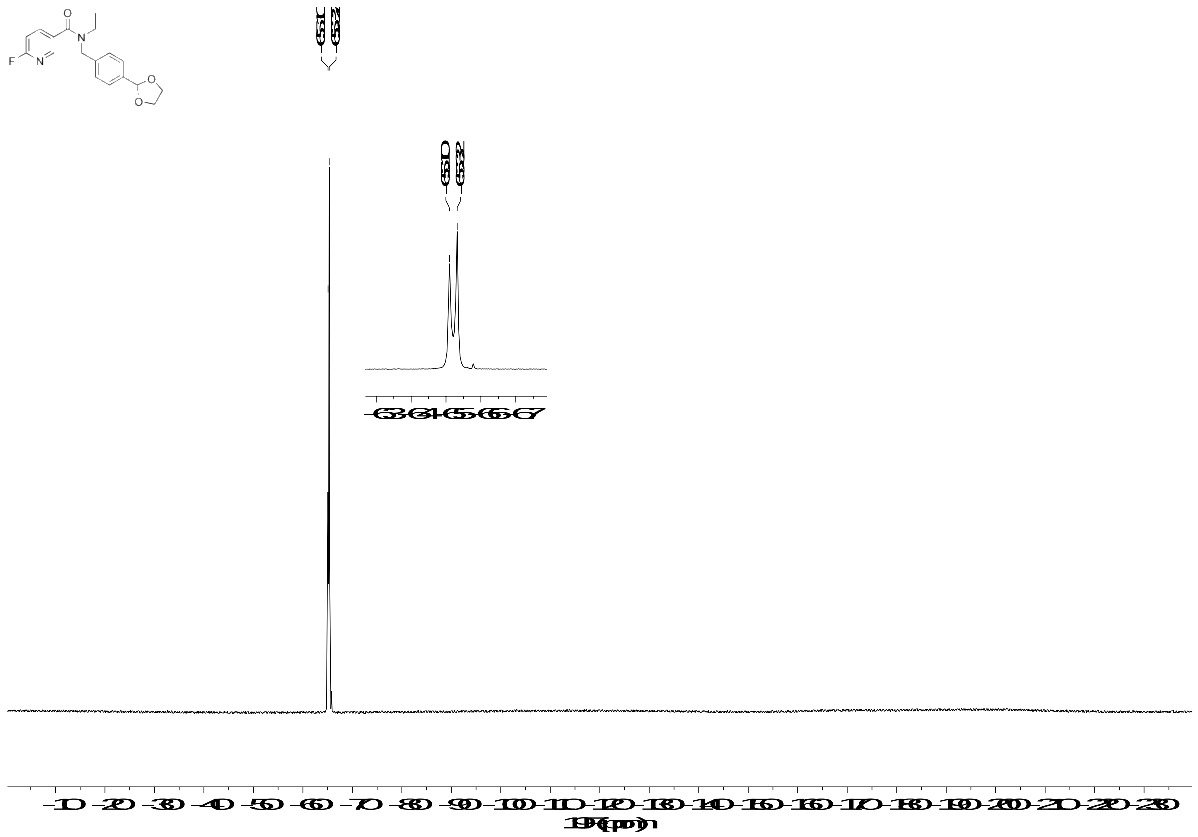

^{1}H NMR (700 MHz, DMSO-d_6, 298K) of *N*-(4-(1,3-dioxolan-2-yl)benzyl)-N-ethyl-6-fluoronicotinamide **18**

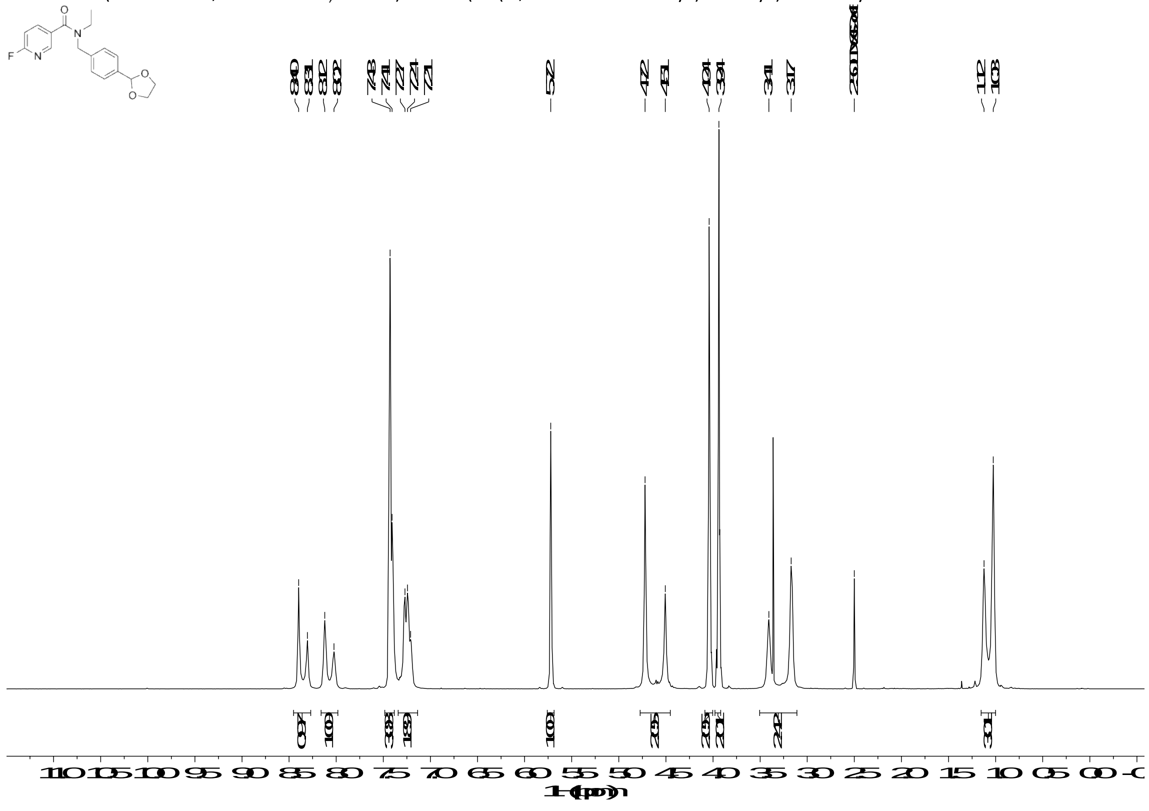

^{13}C NMR (175 MHz, DMSO-d_6, 298K) of *N*-(4-(1,3-dioxolan-2-yl)benzyl)-N-ethyl-6-fluoronicotinamide **18**

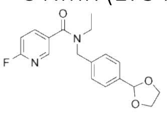

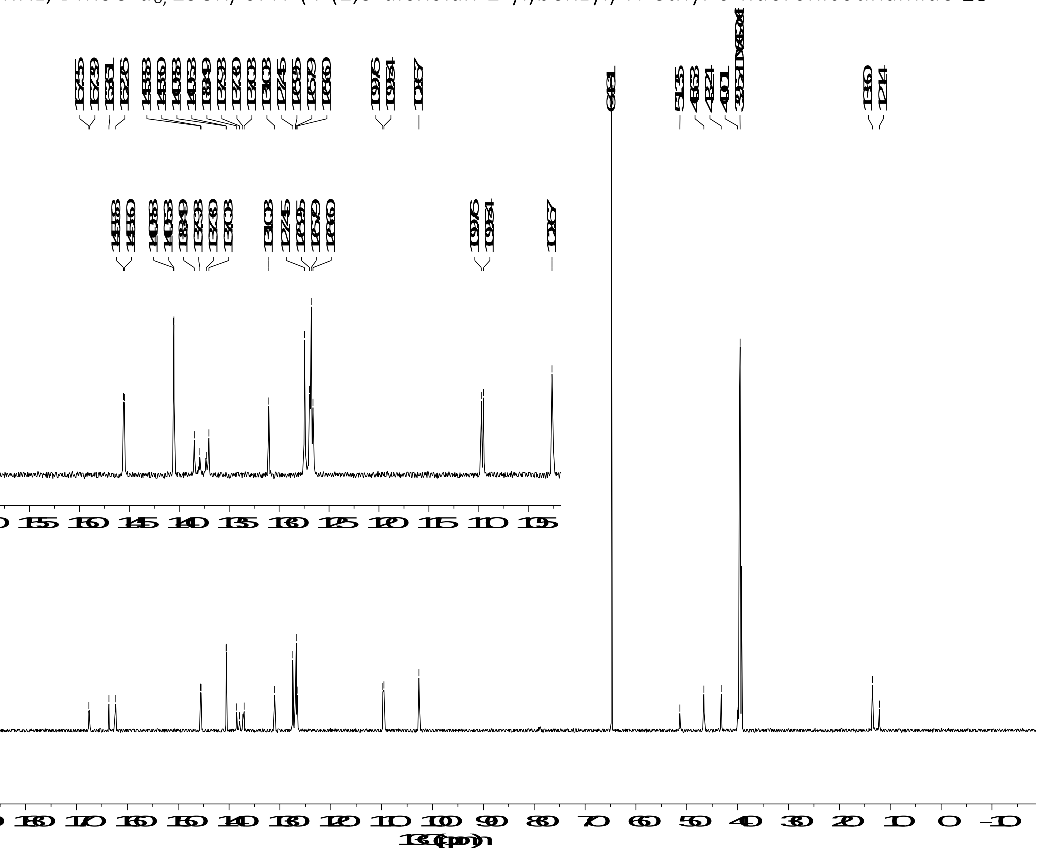

^{19}F NMR (282 MHz, $CDCl_3$, 298K) of *N*-ethyl-6-fluoro-*N*-(4-formylbenzyl)nicotinamide **4b**

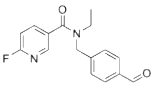

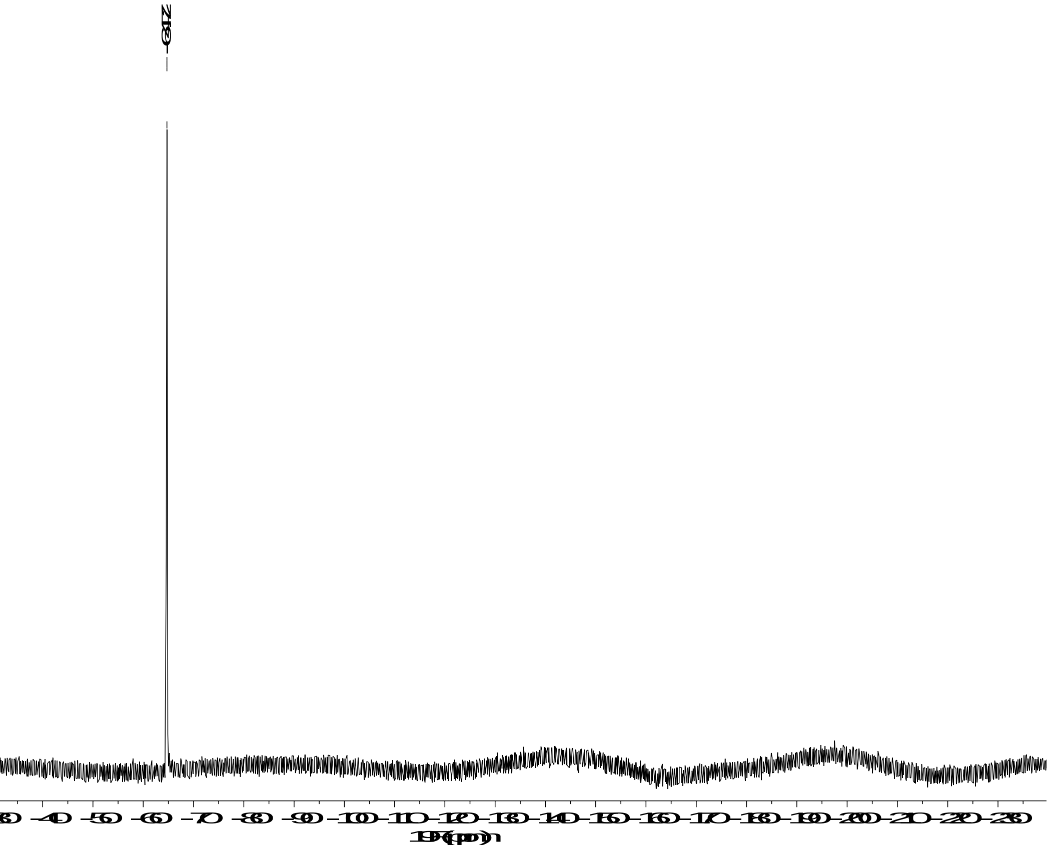

^{1}H NMR (600 MHz, $CDCl_3$, 298K) of *N*-ethyl-6-fluoro-*N*-(4-formylbenzyl)nicotinamide **4b**

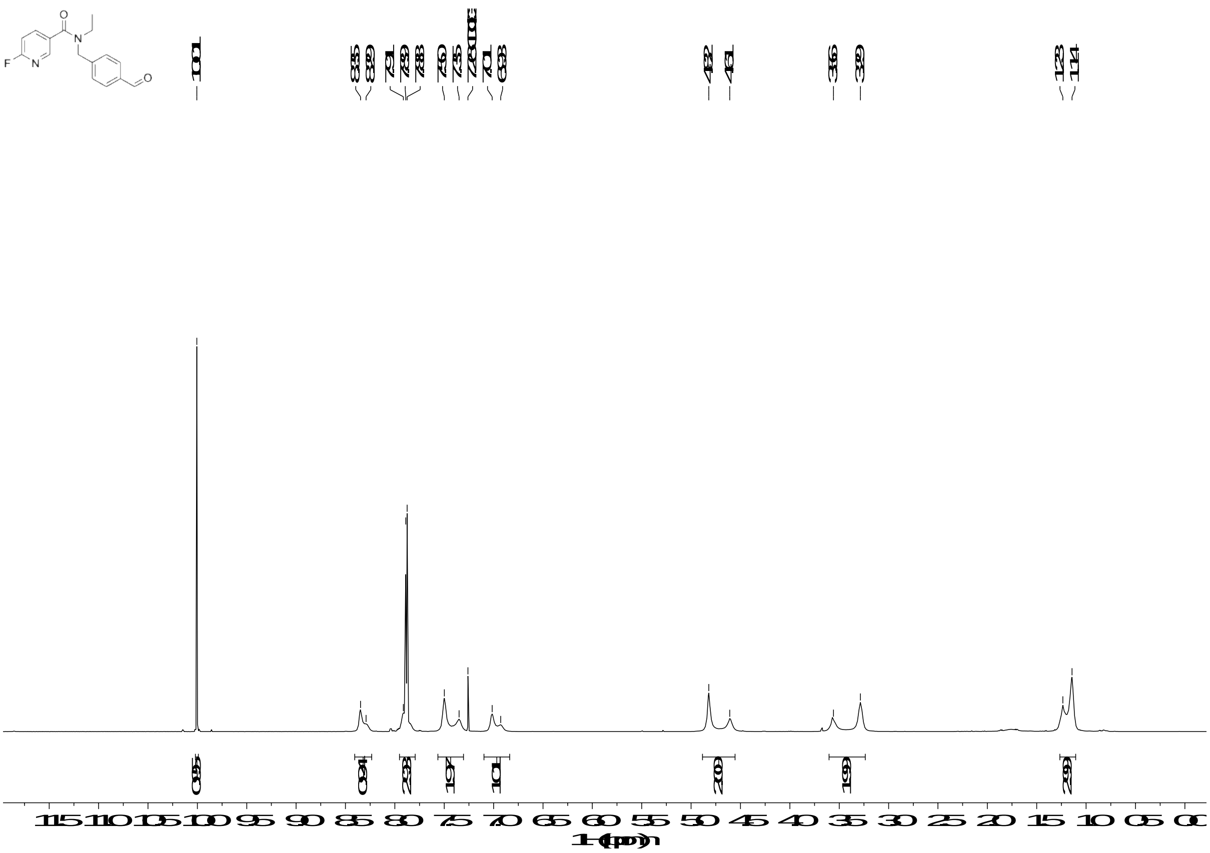

^{13}C NMR (150 MHz, $CDCl_3$, 298K) of *N*-ethyl-6-fluoro-*N*-(4-formylbenzyl)nicotinamide **4b**

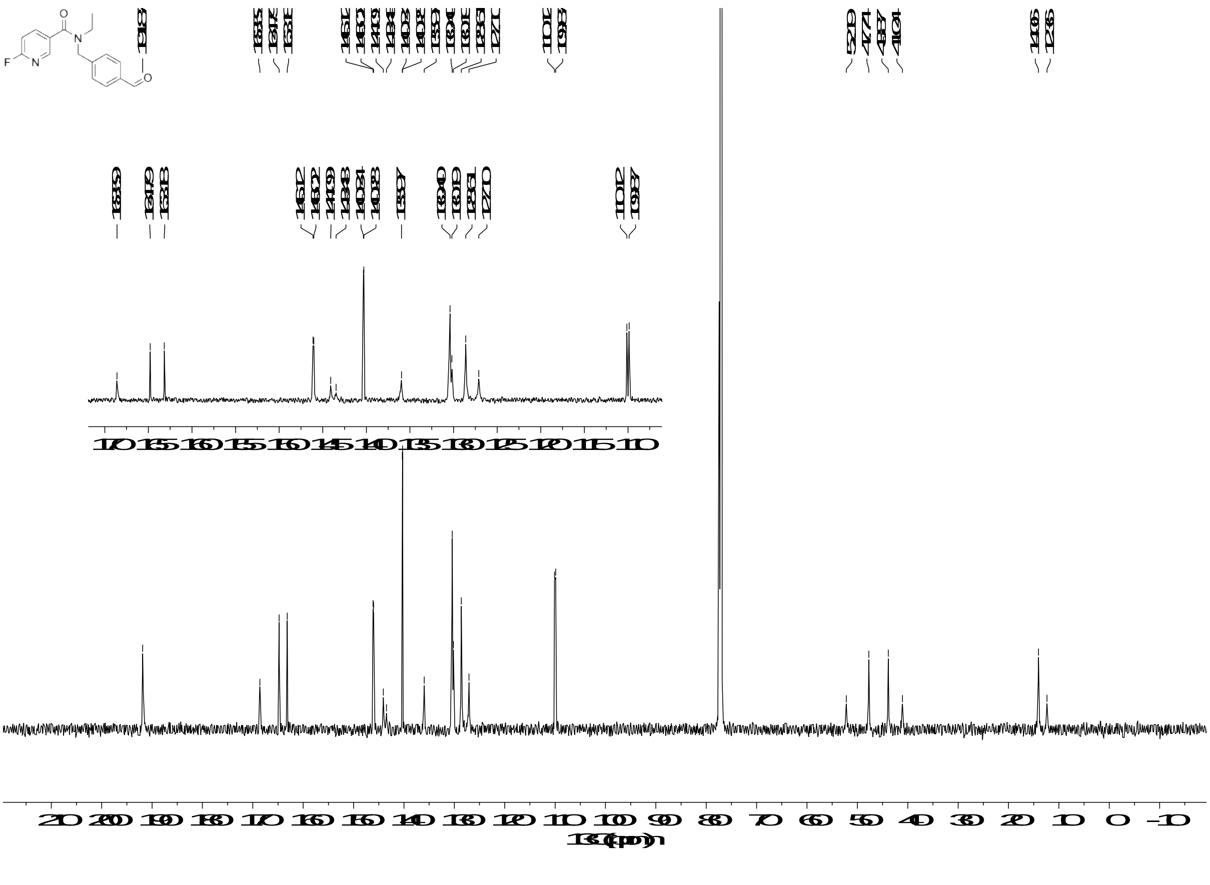

^{19}F NMR (282 MHz, $CDCl_3$, 298K) of 4-((ethyl((5-fluoropyridin-3-yl) methyl)amino)methyl) benzaldehyde **5**

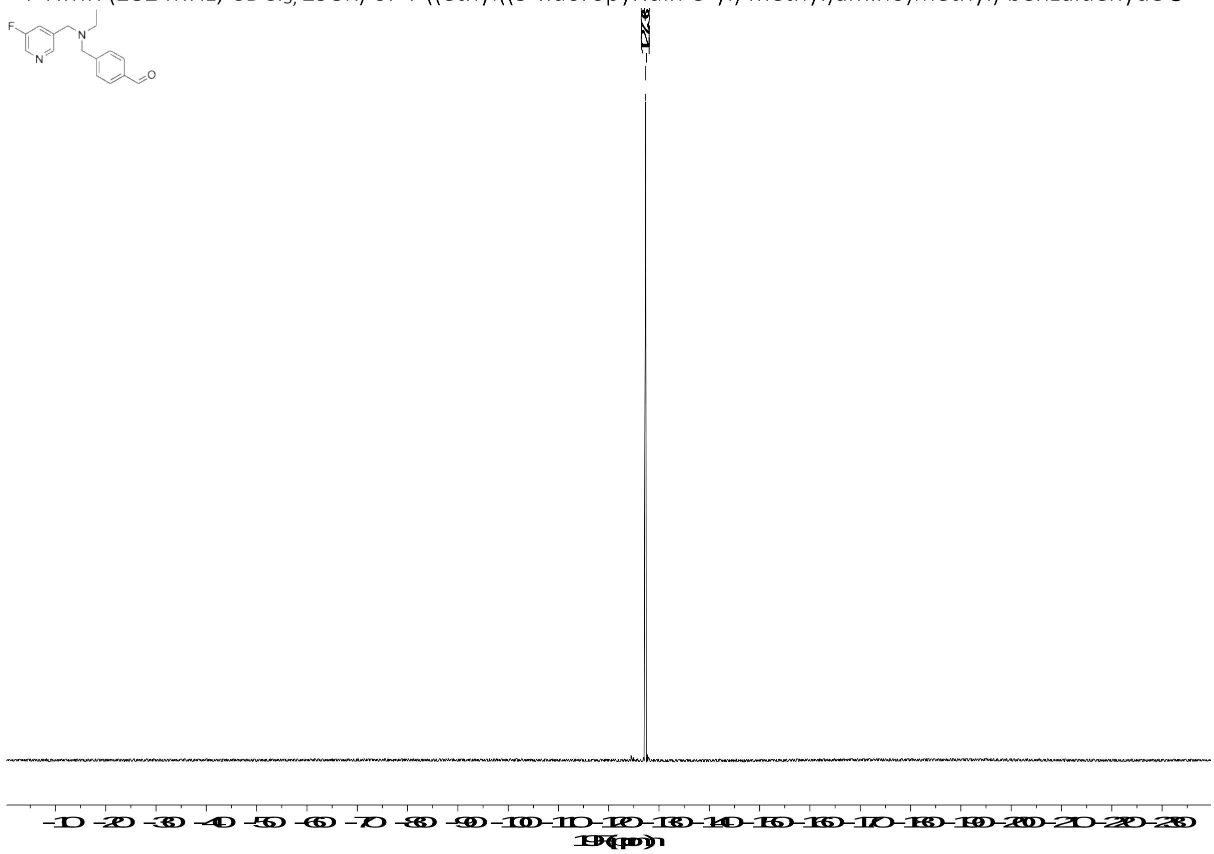

^{1}H NMR (500 MHz, $CDCl_3$, 298K) of 4-((ethyl((5-fluoropyridin-3-yl)methyl)amino)methyl)benzaldehyde **5**

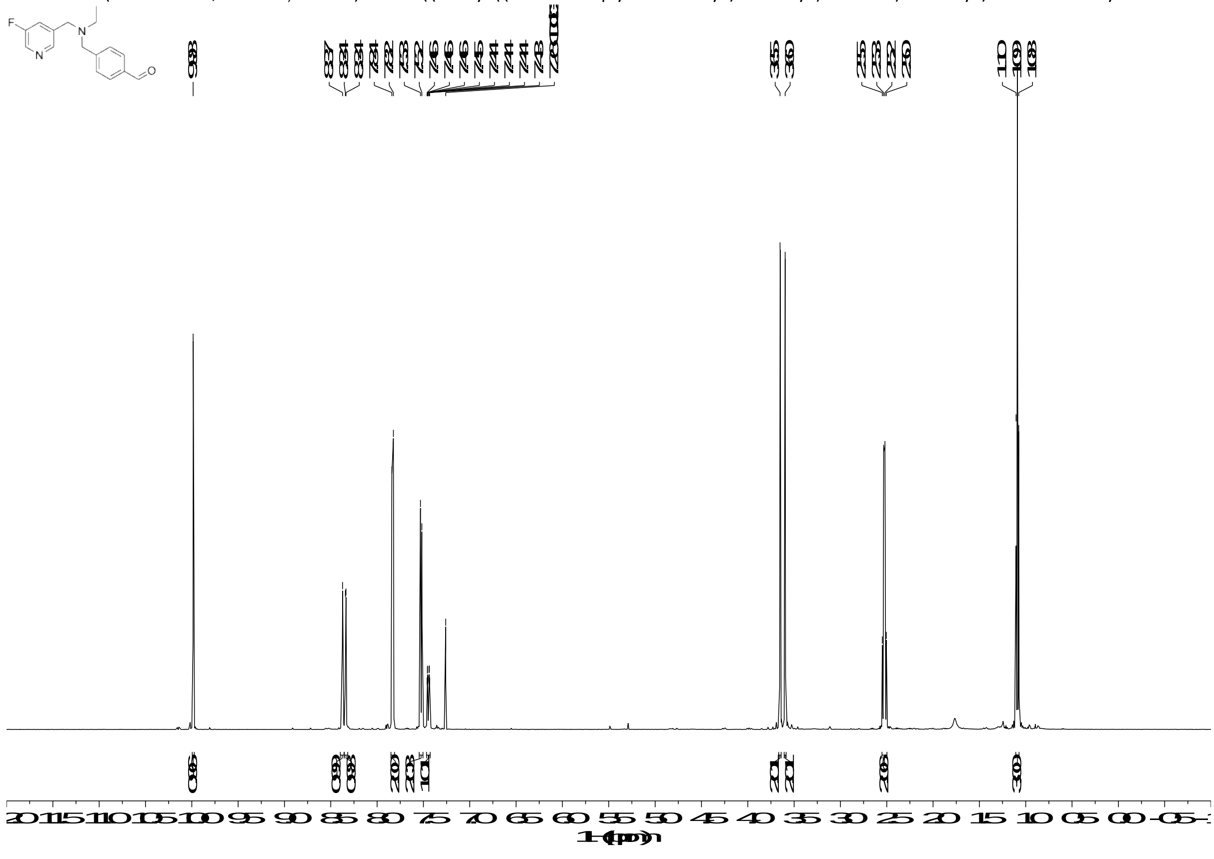

^{13}C NMR (125 MHz, $CDCl_3$, 298K) of 4-((ethyl((5-fluoropyridin-3-yl)methyl)amino)methyl)benzaldehyde **5**

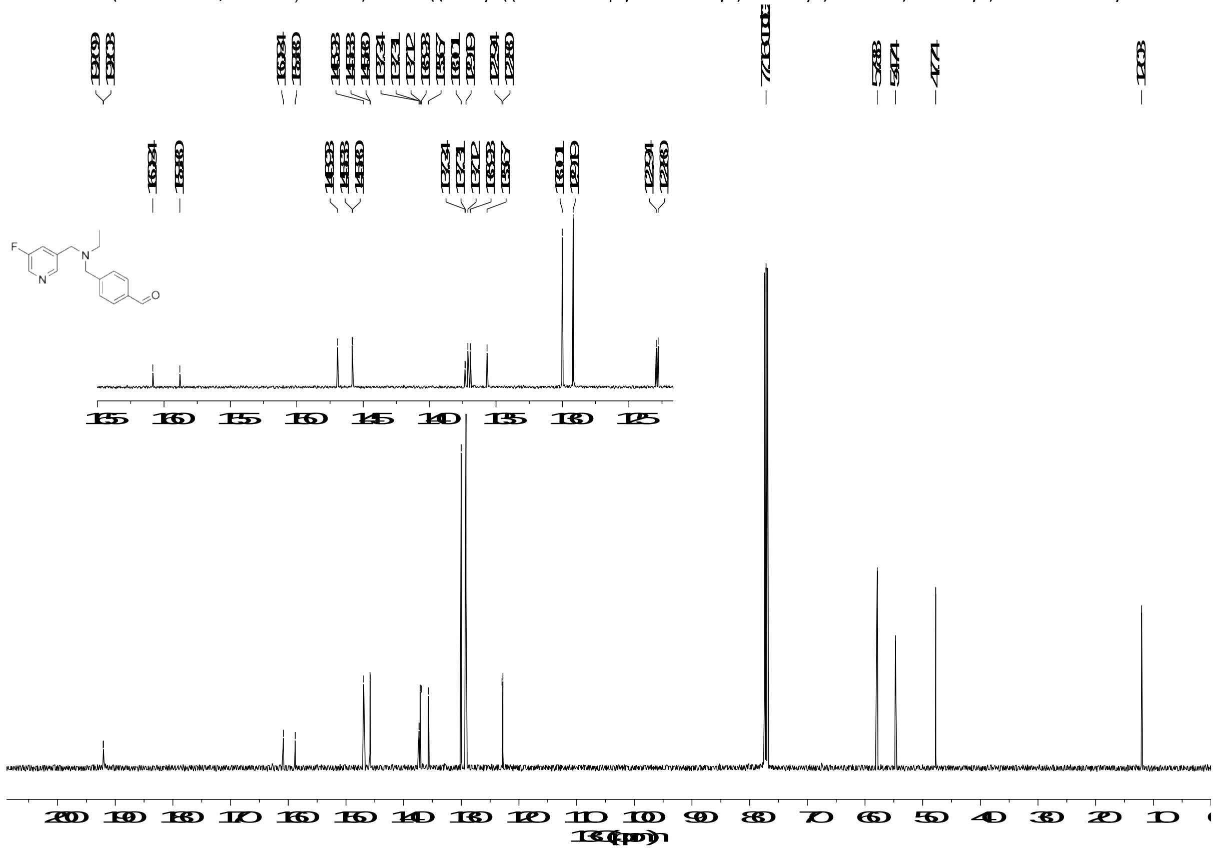

^{19}F NMR (282 MHz, $CDCl_3$, 298K) of N-ethyl-5-fluoro-N-(4-formylbenzyl)pyridine-3-sulfonamide **6**

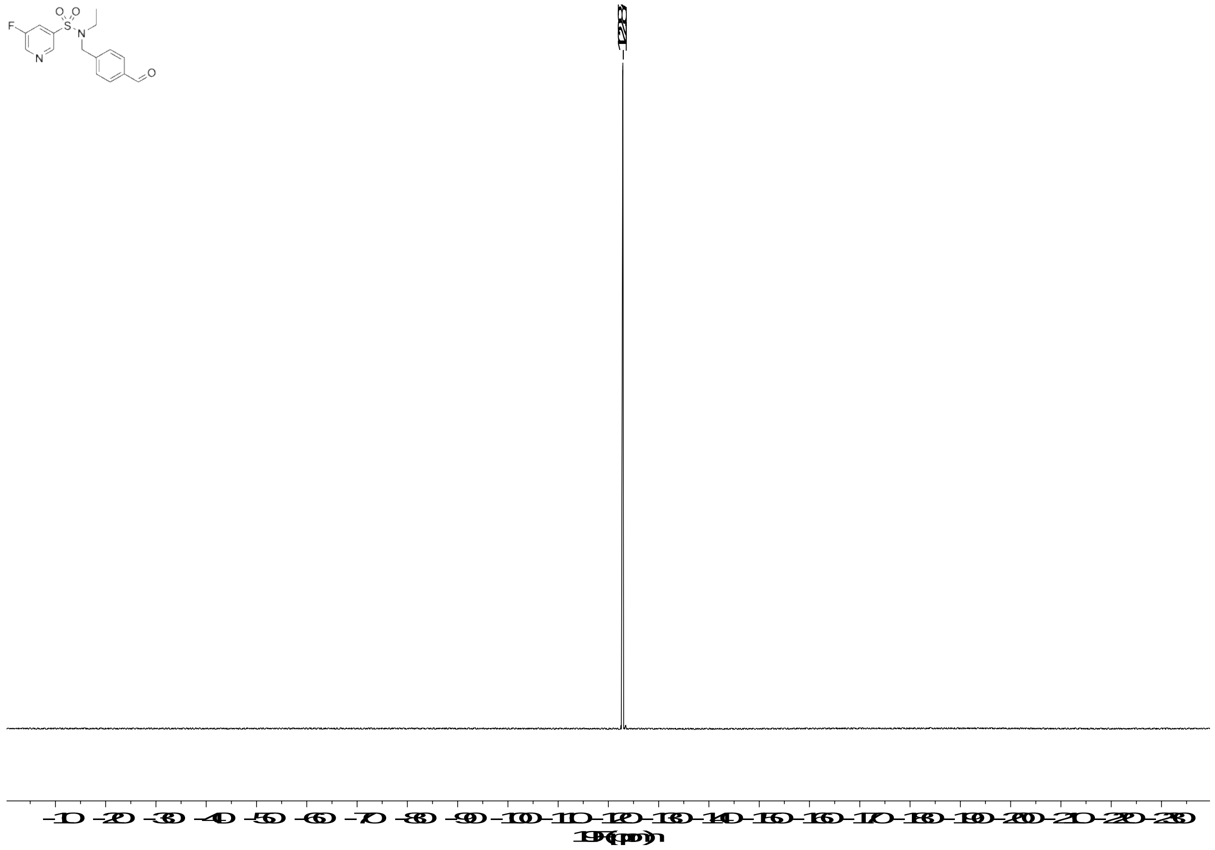

^{1}H NMR (300 MHz, $CDCl_3$, 298K) of N-ethyl-5-fluoro-N-(4-formylbenzyl)pyridine-3-sulfonamide **6**

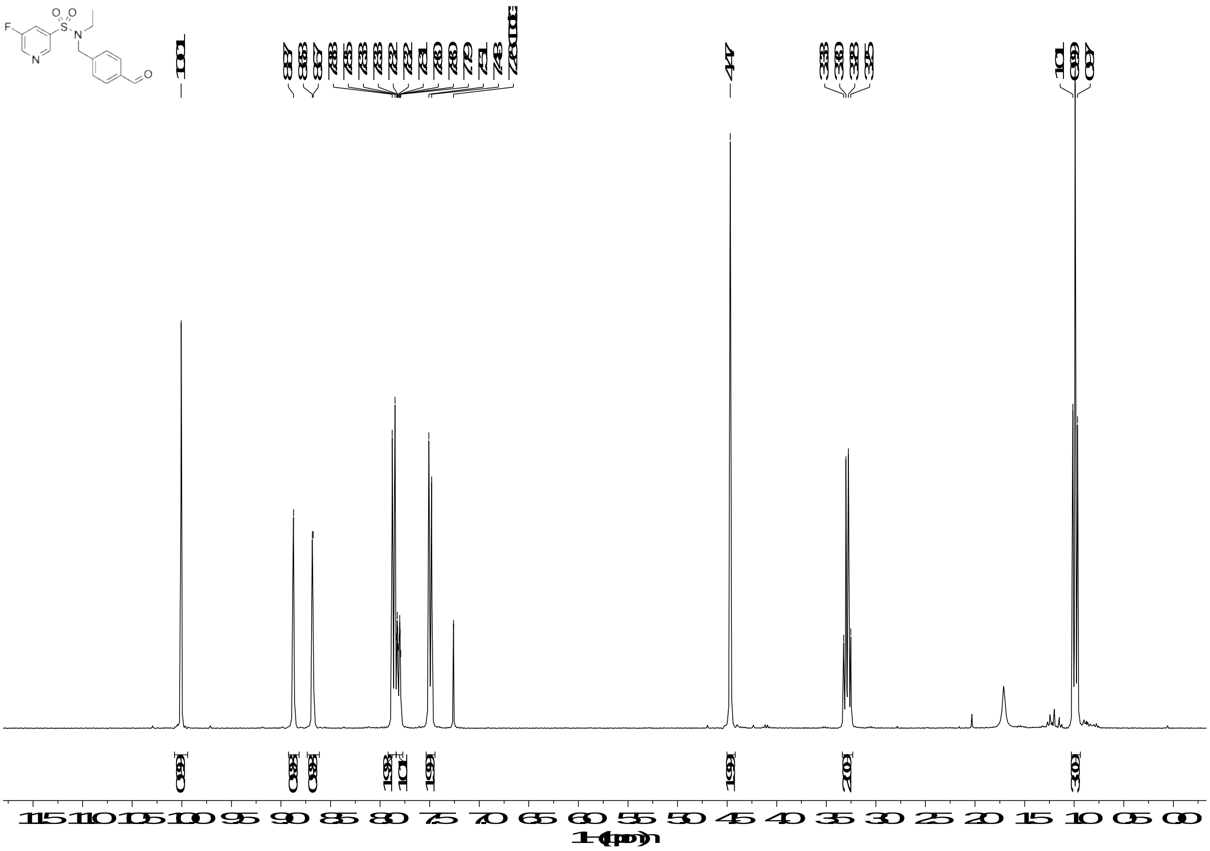

^{13}C NMR (75 MHz, $CDCl_3$, 298K) of N-ethyl-5-fluoro-N-(4-formylbenzyl)pyridine-3-sulfonamide **6**

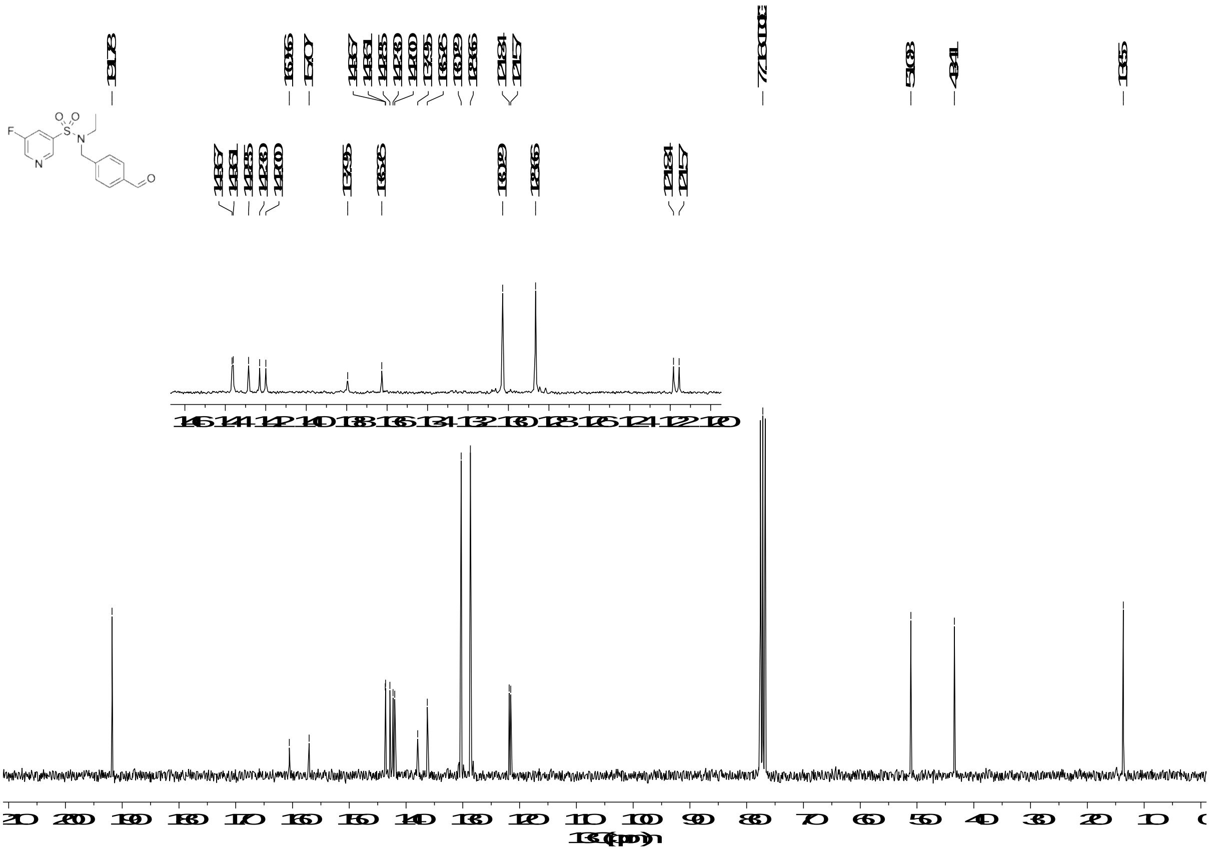

^{19}F NMR (282 MHz, DMSO-d_6, 298K) of 4-((N-ethyl-6-fluoronicotinamido)methyl)benzoic acid **8**

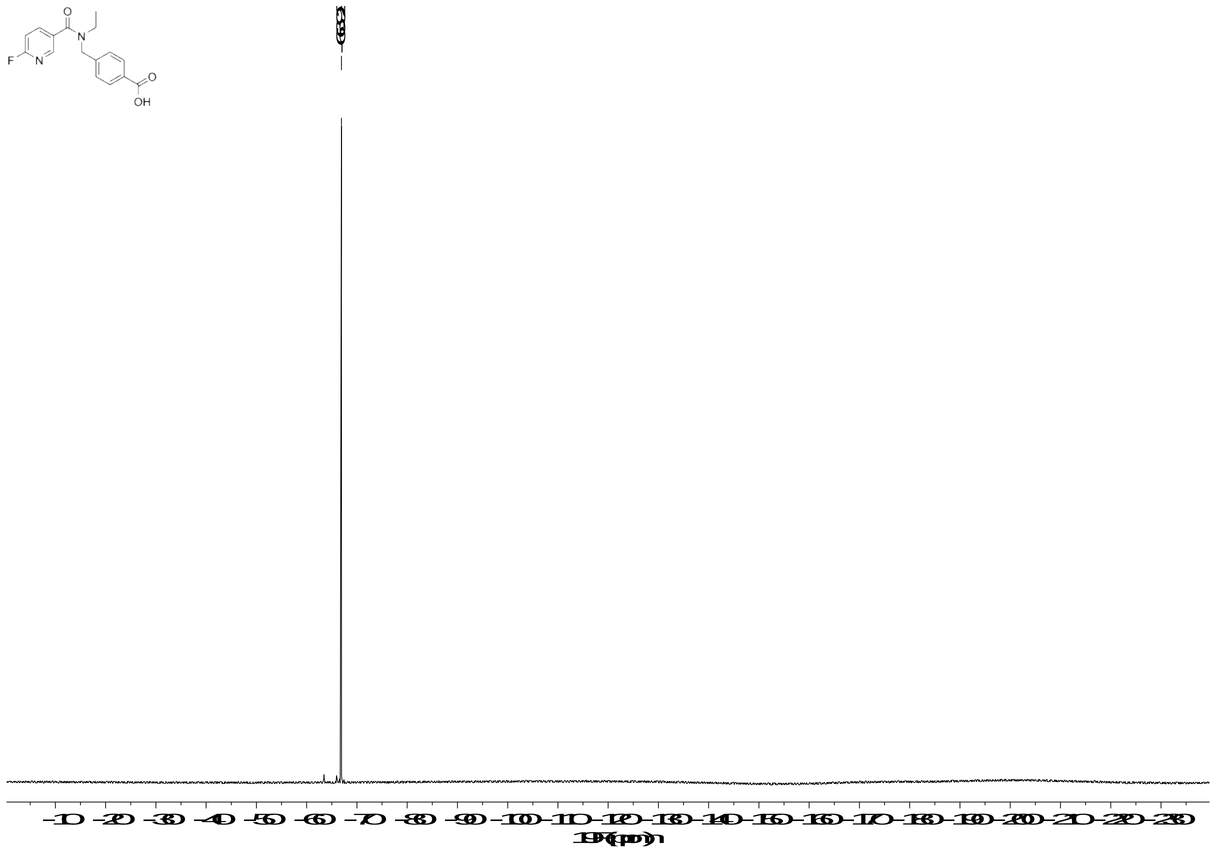

^{1}H NMR (600 MHz, DMSO-d_6, 298K) of 4-((N-ethyl-6-fluoronicotinamido)methyl)benzoic acid **8**

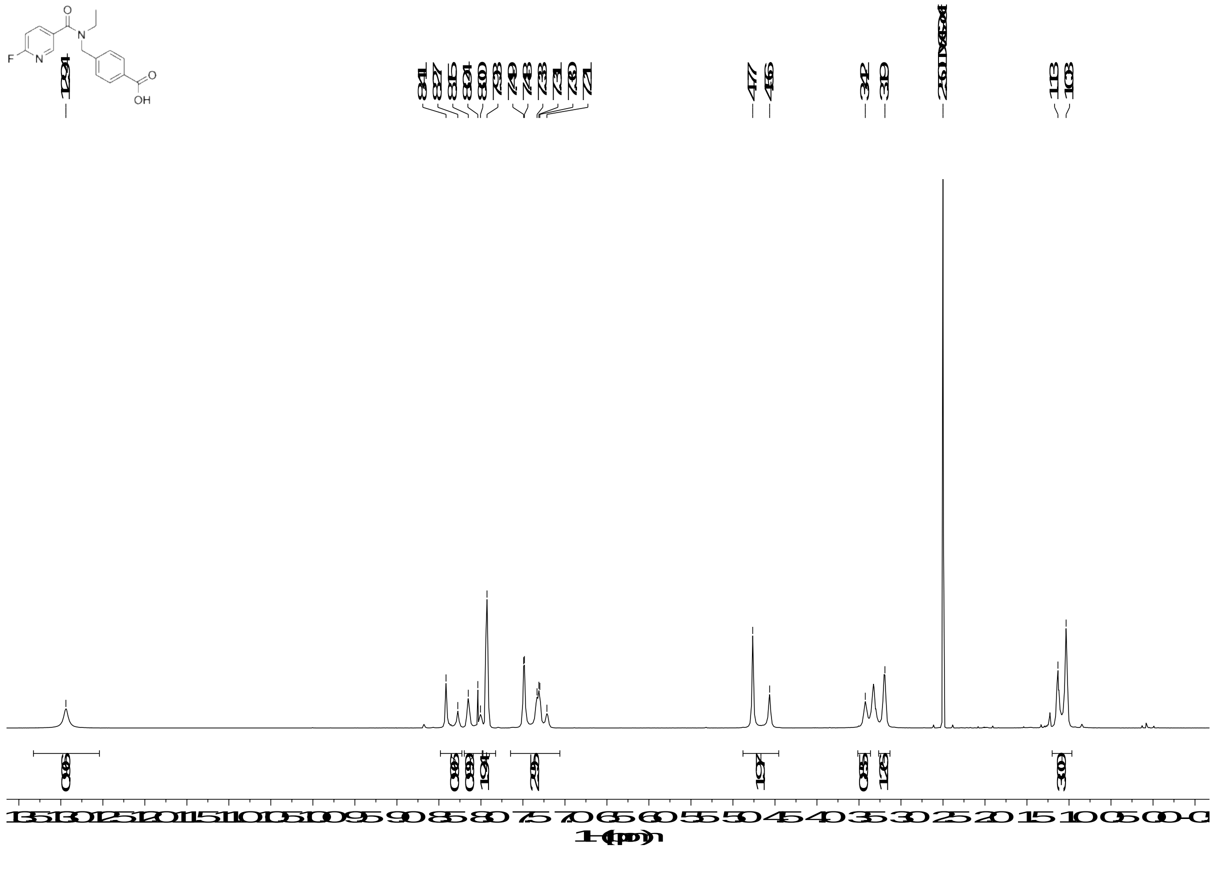

^{13}C NMR (150 MHz, DMSO-d_6, 298K) of 4-((N-ethyl-6-fluoronicotinamido)methyl)benzoic acid **8**

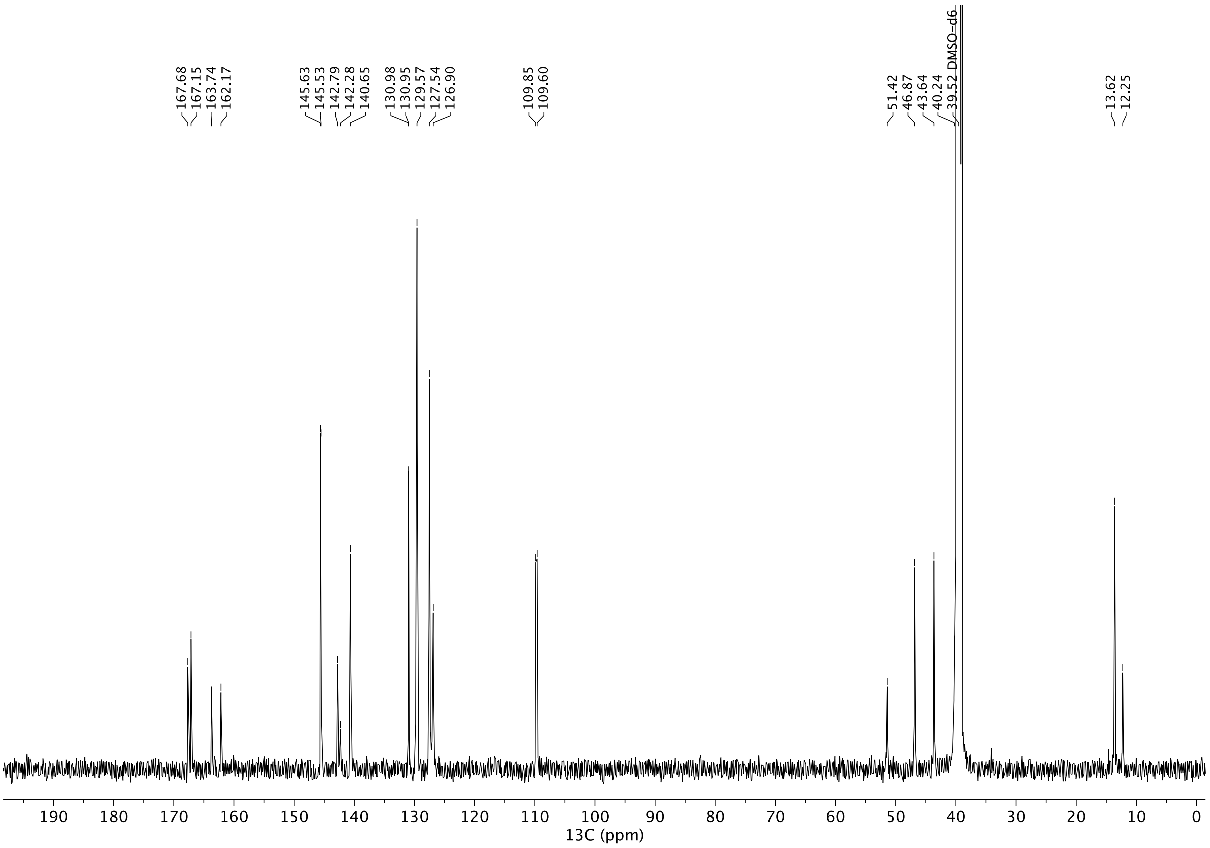

^{19}F NMR (282 MHz, $CDCl_3$, 298K) of N-ethyl-6-fluoro-N-(4-(hydroxymethyl)benzyl)nicotinamide **9**

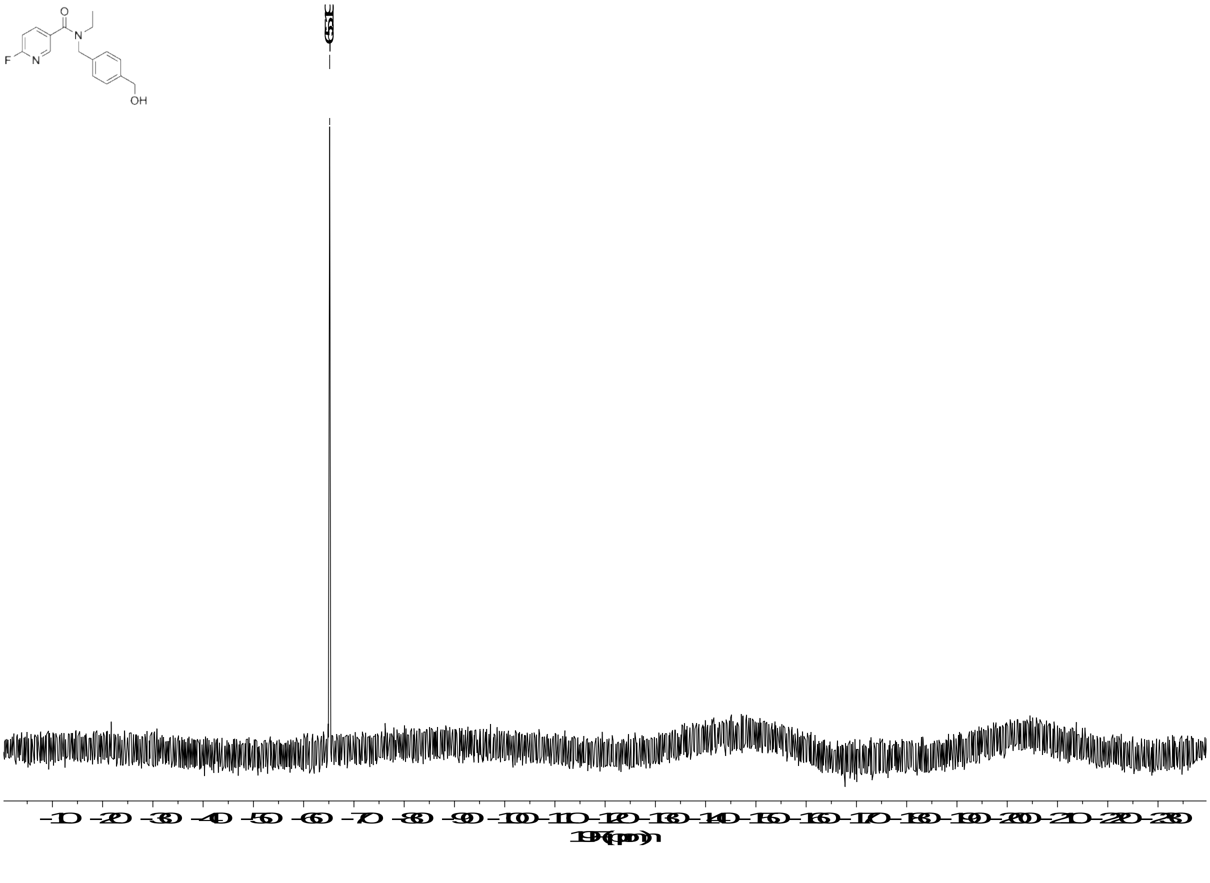

^{1}H NMR (600 MHz, $CDCl_3$, 298K) of N-ethyl-6-fluoro-N-(4-(hydroxymethyl)benzyl)nicotinamide **9**

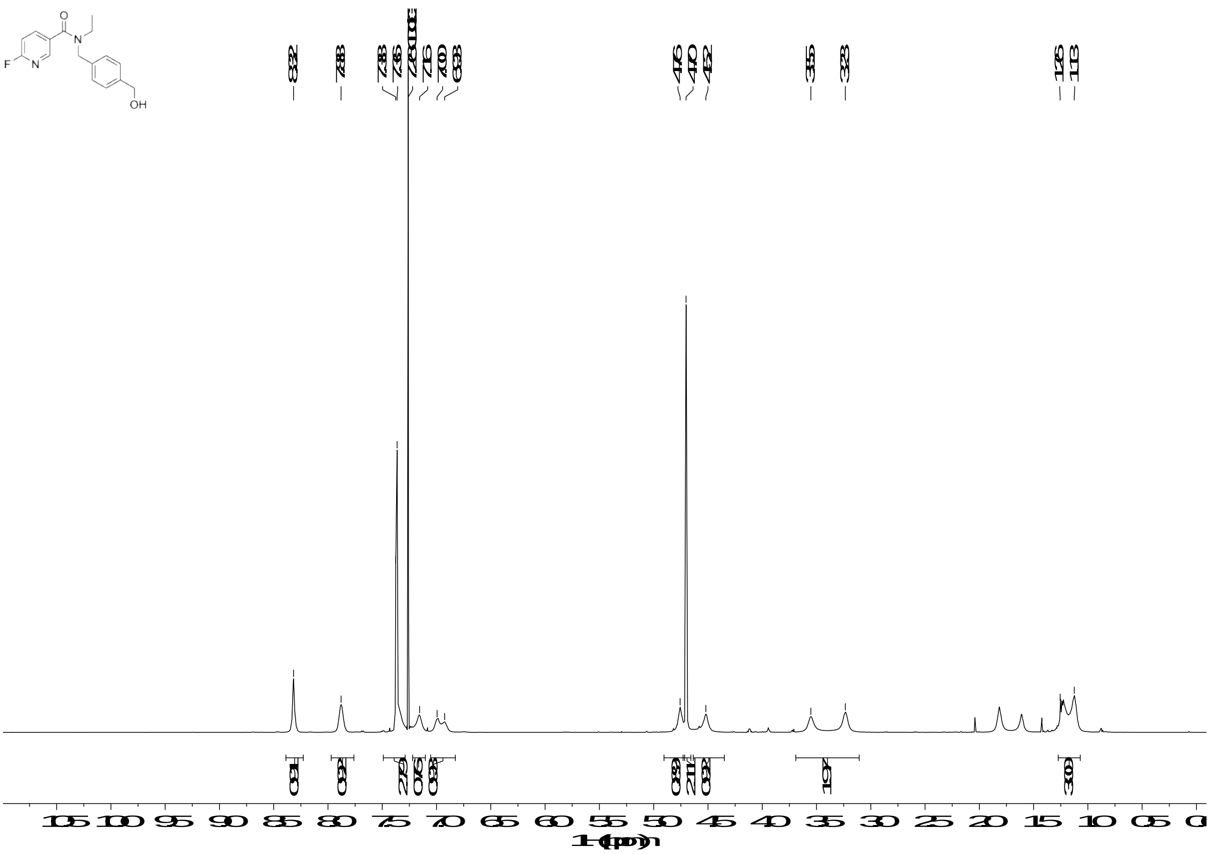

^{13}C NMR (150 MHz, $CDCl_3$, 298K) of N-ethyl-6-fluoro-N-(4-(hydroxymethyl)benzyl)nicotinamide **9**

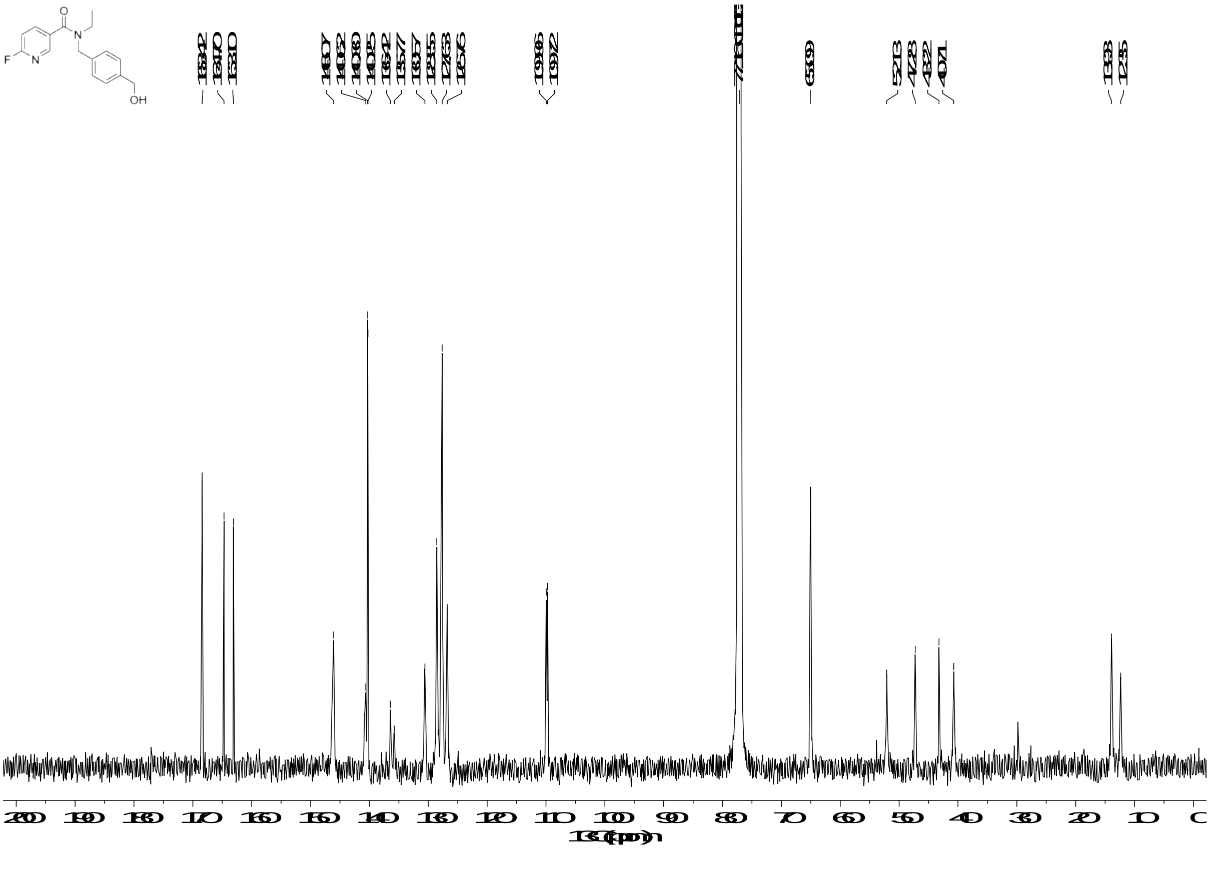

^{1}H NMR (600 MHz, $CDCl_3$, 298K) of *N*-(4-(1,3-dioxolan-2-yl)benzyl)-6-chloro-*N*-ethylnicotinamide **7**

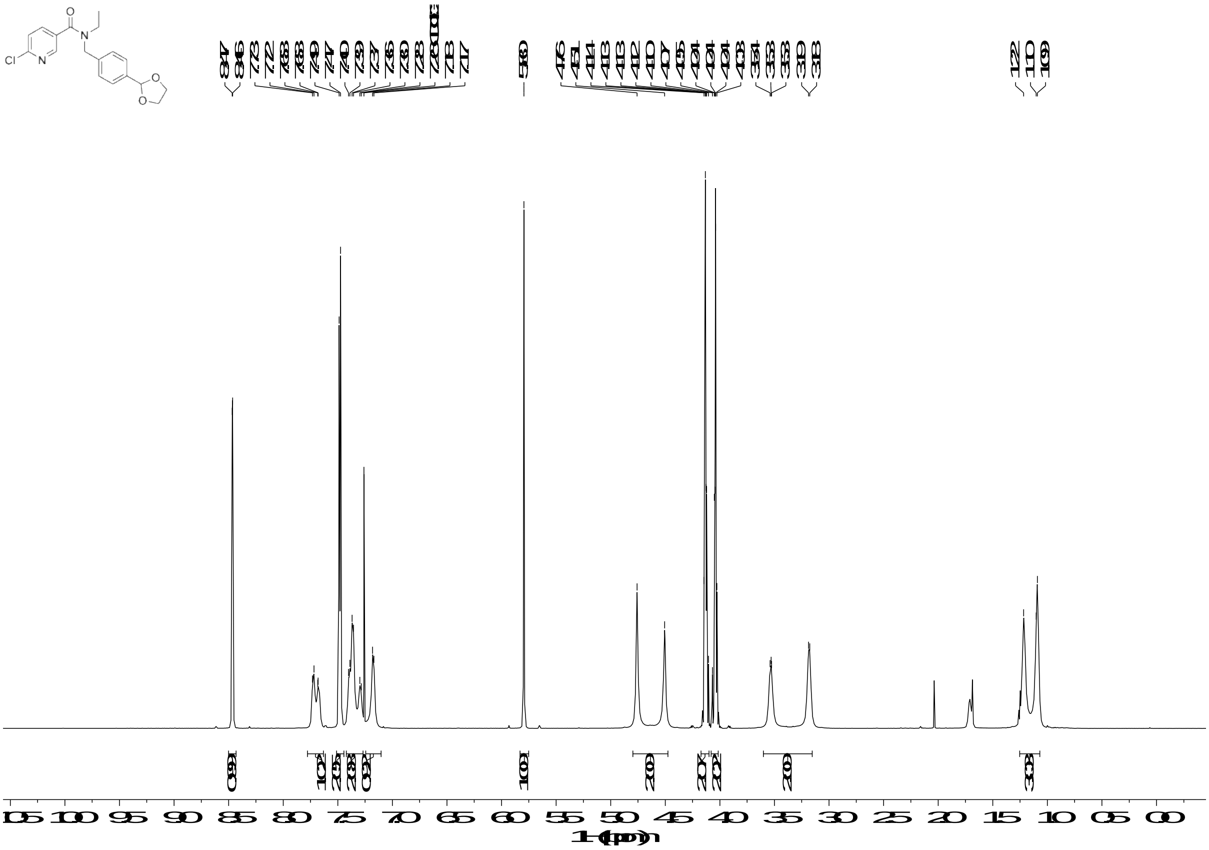

^{13}C NMR (150 MHz, $CDCl_3$, 298K) of *N*-(4-(1,3-dioxolan-2-yl)benzyl)-6-chloro-*N*-ethylnicotinamide **7**

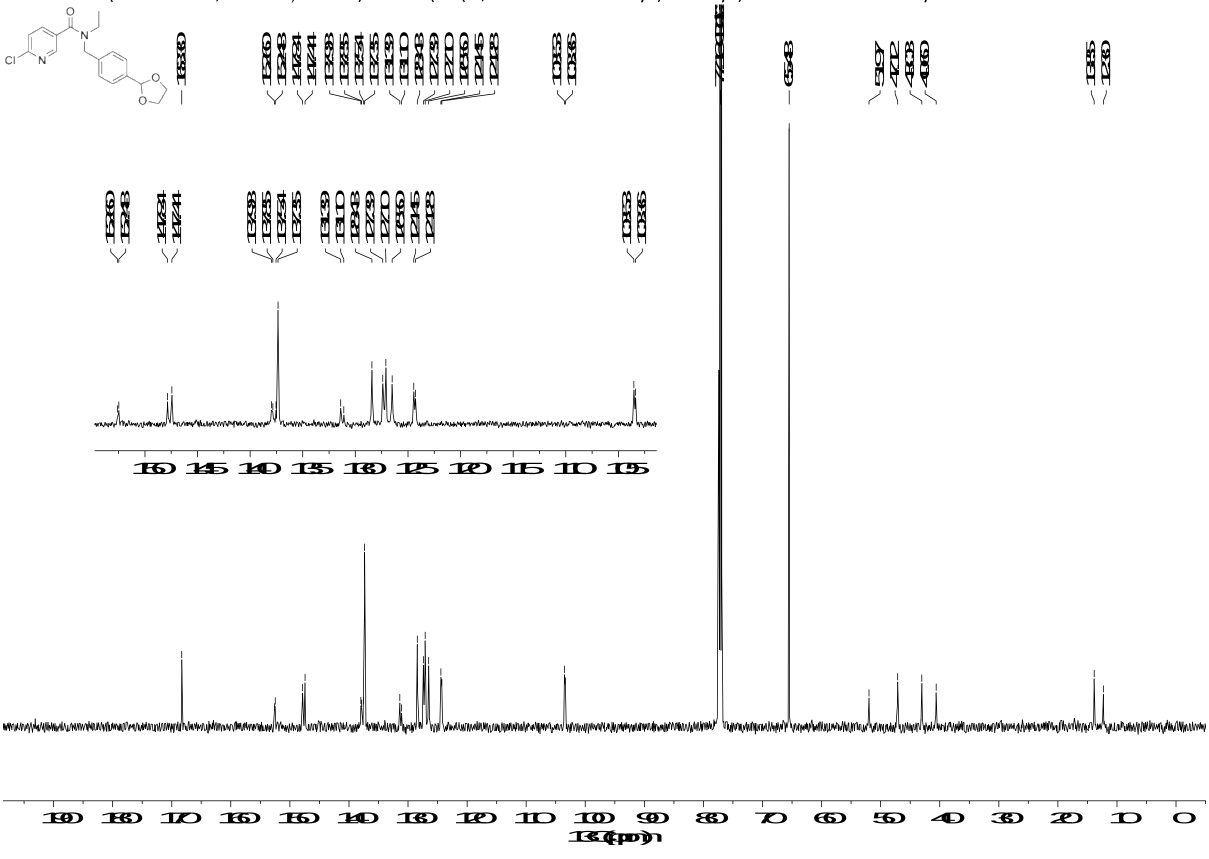

^{1}H NMR (600 MHz, $CDCl_3$, 298K) of 6-chloro-*N*-ethyl-*N*-(4-formylbenzyl)nicotinamide **10**

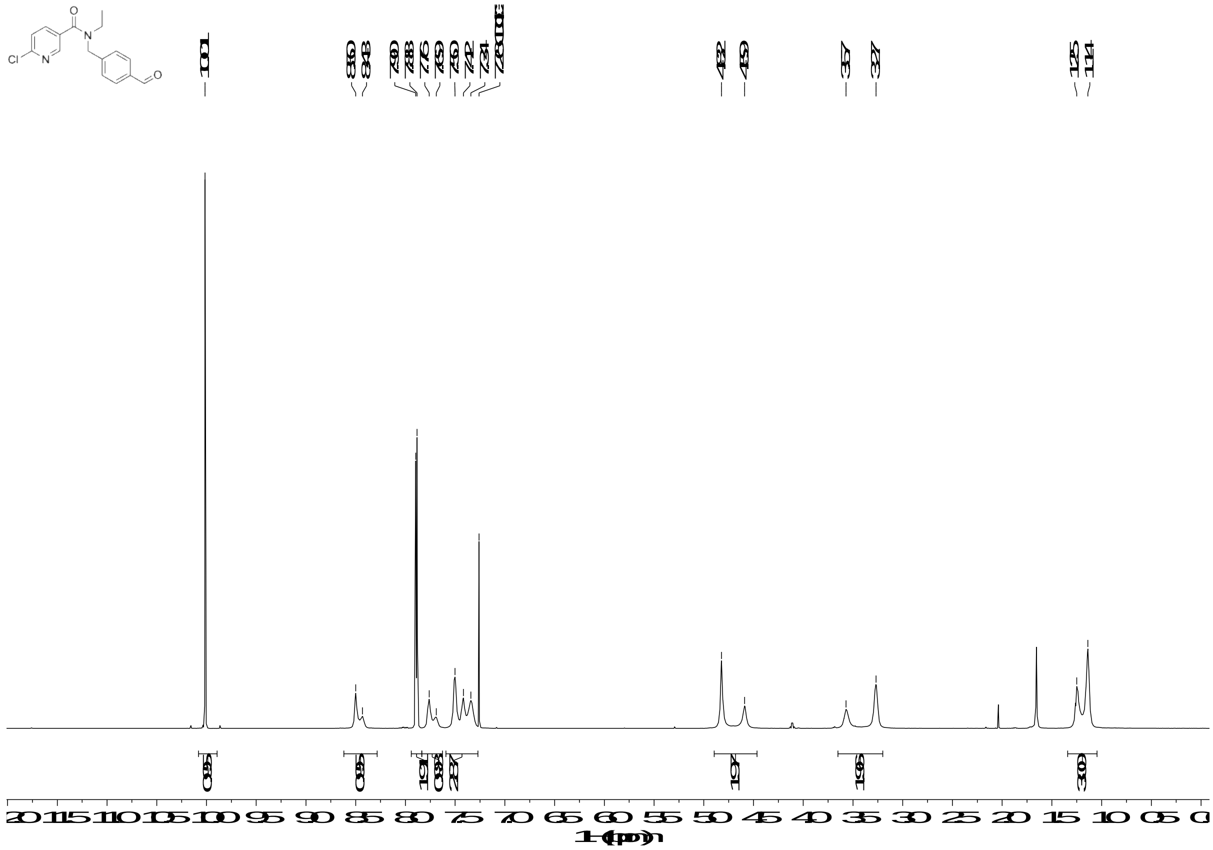

^{13}C NMR (150 MHz, $CDCl_3$, 298K) of 6-chloro-*N*-ethyl-*N*-(4-formylbenzyl)nicotinamide **10**

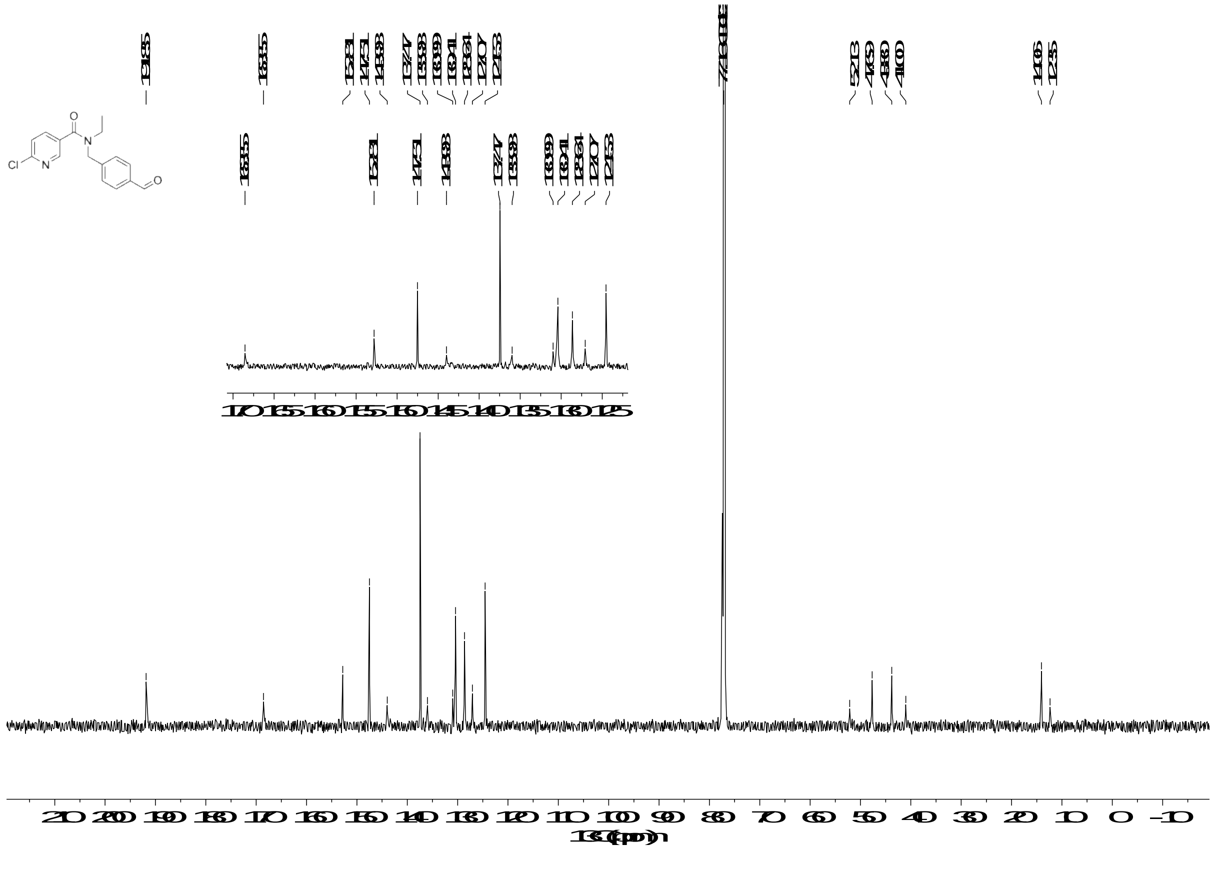

REFERENCES

[1] F. Prati, A. De Simone, A. Armirotti, M. Summa, D. Pizzirani, R. Scarpelli, S. M. Bertozzi, D. I. Perez, V. Andrisano, A. Perez-Castillo, B. Monti, F. Massenzio, L. Polito, M. Racchi, P. Sabatino, G. Bottegoni, A. Martinez, A. Cavalli, M. L. Bolognesi, *ACS Chem. Neurosci*. **2015**, 6, 1665-1682.

[2] H.C. Li, Y.P. Lin, P.T. Chou, Y.M. Chen, R.S. Liu, *Adv. Funct. Mater.* **2007**, 17, 520-530.

[3] C. J. Dinsmore, M. J. Bogusky, J. C. Culberson, J. M. Bergman, C. F. Homnick, C. B. Zartman, S. D. Mosser, M. D. Schaber, R. G. Robinson, K. S. Koblan, H. E. Huber, S. L. Graham, G. D. Hartman, J. R. Huff, T. M. Williams, *J. Am. Chem. Soc*. **2001**, 123, 2107-2108.

[4] S. Imai, H. Togo, *Tetrahedron* **2016**, 72, 6948-6954.

[5] Z.-L. Shen, K. Sommer, P. Knochel, *Synthesis* **2015**, 47, 2617-2630.

[6] The UniProt Consortium, *Nucleic Acids Res*. **2017**, 45, D158-D169.

[7] R. C. Edgar, *Nucleic Acids Res*. **2004**, 32, 1792-1797.

[8] E. F. Pettersen, T. D. Goddard, C. C. Huang, G. S. Couch, D. M. Greenblatt, E. C. Meng, T. E. Ferrin, *J. Comput. Chem*. **2004**, 25, 1605-1612.

[9] V. Le Guilloux, P. Schmidtke, P. Tuffery, *BMC Bioinformatics* **2009**, 10, 168.

[10] O. S. Smart, J. G. Neduvelil, X. Wang, B. A. Wallace, M. S. P. Sansom, *J Mol Graph* **1996**, 14, 354-360.
